# Intranasal Naloxone Repeat Dosing Strategies and Fentanyl Overdose: A Simulation-Based Randomized Clinical Trial

**DOI:** 10.1001/jamanetworkopen.2023.51839

**Published:** 2024-01-23

**Authors:** David G. Strauss, Zhihua Li, Anik Chaturbedi, Shilpa Chakravartula, Mohammadreza Samieegohar, John Mann, Srikanth C. Nallani, Kristin Prentice, Aanchal Shah, Keith Burkhart, Jennifer Boston, Yu-Hui Ann Fu, Albert Dahan, Issam Zineh, Jeffry A. Florian

**Affiliations:** 1 Division of Applied Regulatory Science, Office of Clinical Pharmacology, Office of Translational Sciences, Center for Drug Evaluation and Research, US Food and Drug Administration, Silver Spring, Maryland; 2 Division of Neuropsychiatric Pharmacology, Office of Clinical Pharmacology, Office of Translational Sciences, Center for Drug Evaluation and Research, US Food and Drug Administration. Silver Spring, Maryland; 3 Booz Allen Hamilton, McLean, Virginia; 4 Spaulding Clinical Research, West Bend, Wisconsin; 5 KCAS Bioanalytical Services, Olathe, Kansas; 6 Department of Anesthesiology, Leiden University Medical Center, Leiden, the Netherlands; 7 Office of Clinical Pharmacology, Office of Translational Sciences, Center for Drug Evaluation and Research, US Food and Drug Administration, Silver Spring, Maryland

## Abstract

**Importance:**

Questions have emerged as to whether standard intranasal naloxone dosing recommendations (ie, 1 dose with readministration every 2-3 minutes if needed) are adequate in the era of illicitly manufactured fentanyl and its derivatives (hereinafter, fentanyl).

**Objective:**

To compare naloxone plasma concentrations between different intranasal naloxone repeat dosing strategies and to estimate their effect on fentanyl overdose.

**Design, Setting, and Participants:**

This unblinded crossover randomized clinical trial was conducted with healthy participants in a clinical pharmacology unit (Spaulding Clinical Research, West Bend, Wisconsin) in March 2021. Inclusion criteria included age 18 to 55 years, nonsmoking status, and negative test results for the presence of alcohol or drugs of abuse. Data analysis was performed from October 2021 to May 2023.

**Intervention:**

Naloxone administered as 1 dose (4 mg/0.1 mL) at 0, 2.5, 5, and 7.5 minutes (test), 2 doses at 0 and 2.5 minutes (test), and 1 dose at 0 and 2.5 minutes (reference).

**Main Outcomes and Measures:**

The primary outcome was the first prespecified time with higher naloxone plasma concentration. The secondary outcome was estimated brain hypoxia time following simulated fentanyl overdoses using a physiologic pharmacokinetic-pharmacodynamic model. Naloxone concentrations were compared using paired tests at 3 prespecified times across the 3 groups, and simulation results were summarized using descriptive statistics.

**Results:**

This study included 21 participants, and 18 (86%) completed the trial. The median participant age was 34 years (IQR, 27-50 years), and slightly more than half of participants were men (11 [52%]). Compared with 1 naloxone dose at 0 and 2.5 minutes, 1 dose at 0, 2.5, 5, and 7.5 minutes significantly increased naloxone plasma concentration at 10 minutes (7.95 vs 4.42 ng/mL; geometric mean ratio, 1.95 [1-sided 97.8% CI, 1.28-∞]), whereas 2 doses at 0 and 2.5 minutes significantly increased the plasma concentration at 4.5 minutes (2.24 vs 1.23 ng/mL; geometric mean ratio, 1.98 [1-sided 97.8% CI, 1.03-∞]). No drug-related serious adverse events were reported. The median brain hypoxia time after a simulated fentanyl 2.97-mg intravenous bolus was 4.5 minutes (IQR, 2.1-∞ minutes) with 1 naloxone dose at 0 and 2.5 minutes, 4.5 minutes (IQR, 2.1-∞ minutes) with 1 naloxone dose at 0, 2.5, 5, and 7.5 minutes, and 3.7 minutes (IQR, 1.5-∞ minutes) with 2 naloxone doses at 0 and 2.5 minutes.

**Conclusions and Relevance:**

In this clinical trial with healthy participants, compared with 1 intranasal naloxone dose administered at 0 and 2.5 minutes, 1 dose at 0, 2.5, 5, and 7.5 minutes significantly increased naloxone plasma concentration at 10 minutes, whereas 2 doses at 0 and 2.5 minutes significantly increased naloxone plasma concentration at 4.5 minutes. Additional research is needed to determine optimal naloxone dosing in the community setting.

**Trial Registration:**

ClinicalTrials.gov Identifier: NCT04764630

## Key Points

**Question:**

How long does it take for different repeat dosing strategies of intranasal naloxone to increase naloxone plasma concentration after fentanyl overdose?

**Findings:**

This crossover randomized clinical trial included 21 healthy participants. Compared with 1 intranasal dose of 4 mg of naloxone administered at 0 and 2.5 minutes, 1 dose at 0, 2.5, 5, and 7.5 minutes significantly increased naloxone plasma concentration at 10 minutes, whereas 2 doses at 0 and 2.5 minutes significantly increased naloxone plasma concentration at 4.5 minutes.

**Meaning:**

These findings suggest that further evaluation of community naloxone dosing strategies is warranted.

## Teaser

This randomized clinical trial examines naloxone plasma concentrations between different intranasal naloxone repeat dosing strategies to estimate their effect on fentanyl overdose.

## Introduction

Naloxone is a mu-opioid receptor antagonist approved by the US Food and Drug Administration (FDA) to reverse opioid-induced respiratory depression.^1,2,3^ Following a rise in opioid overdoses and deaths, including from fentanyl and its derivatives (hereinafter, fentanyl),^4,5^ the FDA approved specific naloxone products for use by laypersons as single-use autoinjectors and intranasal sprays.^1,6,7^ Due to challenges in conducting clinical efficacy trials in the community setting, these approvals were based on demonstrating that naloxone plasma concentrations are comparable to or greater than those achieved by approved, labeled routes of administration.^8^

Intranasal naloxone products are sold in packages with 2 single-use nasal sprays and are approved for administration as a single dose with repeat doses every 2 to 3 minutes if the patient does not respond. Questions have emerged as to whether current naloxone dosing is adequate in the era of illicitly manufactured fentanyl, because of fentanyl’s potential to induce rapid respiratory depression and death and the observation that higher naloxone doses have been required.^9,10,11^ In addition, there are limited clinical data on repeat intranasal dosing, which can result in less-than-dose-proportional increases in plasma concentration with intranasal drugs.^12,13^

To address these data gaps, the FDA conducted a randomized clinical trial in healthy participants to compare naloxone plasma concentrations between different naloxone repeat dosing strategies and to estimate the effect of naloxone dosing strategies on rescuing patients from simulated fentanyl or carfentanil^14,15,16^ overdoses with a previously validated physiologic pharmacokinetic-pharmacodynamic model.^17^

## Methods

### Study Design and Setting

This unblinded, 3-period, crossover randomized clinical trial was conducted in healthy participants at a clinical pharmacology unit (Spaulding Clinical Research, West Bend, Wisconsin) in March 2021. The trial compared the pharmacokinetics of intranasal naloxone between different repeat dosing strategies and incorporated the data into a previously validated physiologic pharmacokinetic-pharmacodynamic model^17^ to estimate the effect of naloxone dosing strategies on fentanyl and carfentanil overdoses. This trial was approved by the Advarra Institutional Review Board. All participants provided written informed consent. The study followed the Consolidated Standards of Reporting Trials (CONSORT) reporting guideline. The trial protocol, statistical analysis plan, and model analysis plan are available in Supplement 1.

### Participants and Randomization

Participants were recruited with standard approaches for healthy participant clinical pharmacology trials (ie, online advertising and emails or texts sent to individuals in the Spaulding Clinical Research database). Key inclusion criteria included age 18 to 55 years, nonsmoking status, and negative test results for the presence of alcohol or drugs of abuse. Key exclusion criteria included nasal abnormalities or upper respiratory infection in the past month.

**Figure 1.**
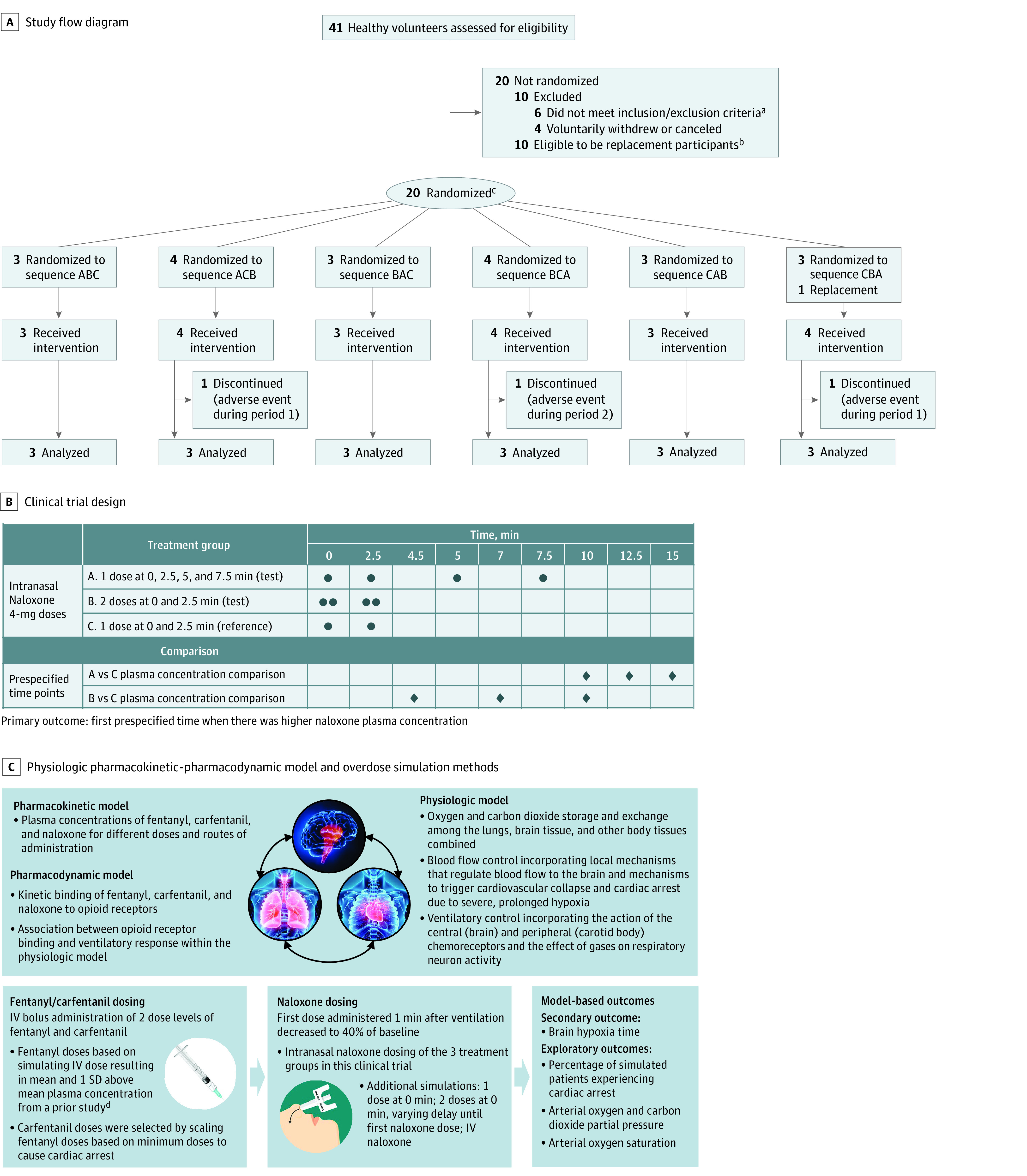
Participant Flow Diagram, Study Design, and Physiologic Pharmacokinetic-Pharmacodynamic Model A, Study flow diagram. B, Clinical trial design. C, Physiologic pharmacokinetic-pharmacodynamic model and overdose simulation methods. IV indicates intravenous. ^a^Six participants did not meet the inclusion or exclusion criteria due to abnormal medical history, laboratory results, or physical examination findings. ^b^Participants were not needed as replacements. ^c^One participant replaced a participant who dropped out on the first day in the first cohort. ^d^Sorg et al.^18^

Participants were randomly assigned to 1 of 6 treatment sequences (Figure 1) using a random number generator in R, version 4.0.2 (R Project for Statistical Computing). Randomization was conducted in block sizes of 6 for the first 18 participants, and the remaining 2 participants were randomly assigned in 2 of the 6 treatment sequences. Replacement participants were assigned to the treatment sequence of the participant they replaced.

Self-identified race (based on US Office of Management and Budget standards) and ethnicity (Hispanic or Latino) were collected in an open-ended format by clinical staff. For reference, race was reported as American Indian or Alaska Native, Asian, Black or African American (hereinafter Black), Native Hawaiian or Pacific Islander, White, or not reported.

### Trial Procedures and Interventions

Participants checked in 1 day before dosing and received the following intranasal naloxone doses (4 mg/0.1 mL of Narcan; Emergent BioSolutions)^6^ in a randomized order on days 1, 4, and 7: 1 dose administered at 0, 2.5, 5, and 7.5 minutes; 2 doses administered at 0 and 2.5 minutes; and 1 dose administered at 0 and 2.5 minutes. Sequential doses were administered to alternating nostrils and participants remained supine for approximately 1 hour after dosing. Each dosing day included 16 plasma samples (0 [predose], 2, 4.5, 7, 10, 12.5, 15, 20, 30, 45, 60, 120, 180, 240, 360, and 720 minutes). Naloxone concentrations were measured with validated liquid chromatography and tandem mass spectrometry (eMethods 1 in Supplement 2). Deidentified participant data are available in Supplement 3 (a data dictionary is provided in the eAppendix in Supplement 2).

### Simulated Patient Outcomes With a Physiologic Pharmacokinetic-Pharmacodynamic Model

The validated model used to estimate patient outcomes was recently described.^17^ This model contains multiple mechanistic submodels (Figure 1 and eMethods 2 in Supplement 2) as follows: (1) a physiologic model describing oxygen and carbon dioxide storage and exchange, ventilatory control, and blood flow control based on the model developed by Ursino and colleagues^19,20,21^; (2) pharmacokinetic and mu-opioid receptor binding models for fentanyl, carfentanil, and naloxone; and (3) a pharmacodynamic model describing the association between opioid-agonist binding to the mu-receptor and ventilatory response within the physiologic model (based on clinical data from chronic opioid users^22^).

Two fentanyl doses (1.63 and 2.97 mg) were selected based on simulation of the intravenous bolus doses that would result in the mean and 1 SD above the mean plasma concentration from a study of approximately 500 unintentional fentanyl overdoses with postmortem data.^18^ Carfentanil doses (0.012 and 0.022 mg) were selected by scaling the fentanyl doses based on the ratio of the minimum carfentanil-to-fentanyl dose estimated to result in cardiac arrest. The first naloxone dose was administered 1 minute after ventilation decreased to 40% of baseline. Codes for generating simulated overdose scenarios can be found in the repeat dosing branch of the team’s GitHub page.

### Prespecified Outcomes

The primary outcome was the first prespecified time point (Figure 1) when there was higher naloxone plasma concentration in the 4-dose groups compared with the 2-dose group. Secondary outcomes included a similar comparison between the 4-dose groups, dose proportionality of the 4-dose groups compared with the 2-dose group (based on area under the plasma concentration-time curve and maximum plasma concentration), and the estimated brain hypoxia time (ie, time brain oxygen partial pressure was <20 mm Hg) following simulated fentanyl and carfentanil intravenous bolus overdoses with the physiologic model. Exploratory outcomes included additional pharmacokinetic parameters and model-based outcomes from the physiologic model including the percentage of simulated patients experiencing cardiac arrest (Figure 1 and eTable 1 in Supplement 2).

Additional naloxone simulations were performed to better understand opioid reversal (Model Analysis Plan in Supplement 1). These simulations included administering 1 intranasal naloxone dose at 0 minutes and 2 intranasal naloxone doses at 0 minutes, varying the delay until administering 1 intranasal naloxone dose, and administering intravenous naloxone following the repeat dosing protocol described by Boyer.^2^

### Statistical Analysis

A sample size of 20 participants was determined to have greater than 90% power to detect an increase in naloxone plasma concentration between the 4-dose groups and the 2-dose group based on prior pharmacokinetic data.^6^ Each of the 4-dose to 2-dose group comparisons were considered separate experiments. Adjustment for multiplicity in comparing multiple time points was done using Pocock boundaries,^23^ corresponding to assessments at a .022 significance level at 3 prespecified times to maintain an overall .05 significance level.

Naloxone concentrations were log transformed and compared using a paired *t* test at 3 prespecified times (Figure 1), starting with the first time after all doses in the 4-dose group were administered. Testing was conducted sequentially until a comparison passed at a 1-sided *P* = .022 (reported as the earliest time where a difference in concentration was observed) or all prespecified times failed. The dose-adjusted maximum plasma concentration and the area under the curve were calculated based on noncompartmental pharmacokinetic parameter results. Naloxone concentration values below the lower limit of quantification were not used in paired comparisons.

Demographics are reported with standard descriptive statistics. The first time point with higher plasma concentration is reported with the geometric mean ratio and 1-sided 97.8% CI at that time point. Dose proportionality assessments are reported as the geometric mean ratio with a 2-sided 90% CI. Simulated patient data are reported as the median and IQR based on 200 randomly selected simulated patients from a population of 2000 with different pharmacokinetic and binding parameters and repeating this 2500 times with replacement. All analyses except for primary outcomes should be interpreted as exploratory because of the potential for type I error due to multiple comparisons. Statistical analyses were performed in R, version 4.0.2 (R Project for Statistical Computing). Data analysis was performed from October 2021 to May 2023.

## Results

### Healthy Clinical Trial Participants and Samples

This trial enrolled 21 participants (20 were originally randomized and 1 was a replacement). Their median age was 34 years (IQR, 27-50 years), and 10 were women (48%) and 11 were men (52%) (eTable 2 in Supplement 2). In terms of race, 9 participants (43%) were Black, 11 (52%) were White, and 1 (5%) was of unknown race. Additionally, 4 participants (19%) reported their ethnicity as Hispanic or Latino. Two participants discontinued the study during period 1 and 1 participant discontinued during period 2; 18 participants (86%) completed the trial (Figure 1). Of the 864 plasma samples in this study, 3 (0.4%) were below the lower limit of quantification and 30 (4%) were outside of the protocol-specified collection time (eTable 3 in Supplement 2).

#### Primary Outcome: Naloxone Plasma Concentration Comparisons

**Figure 2.**
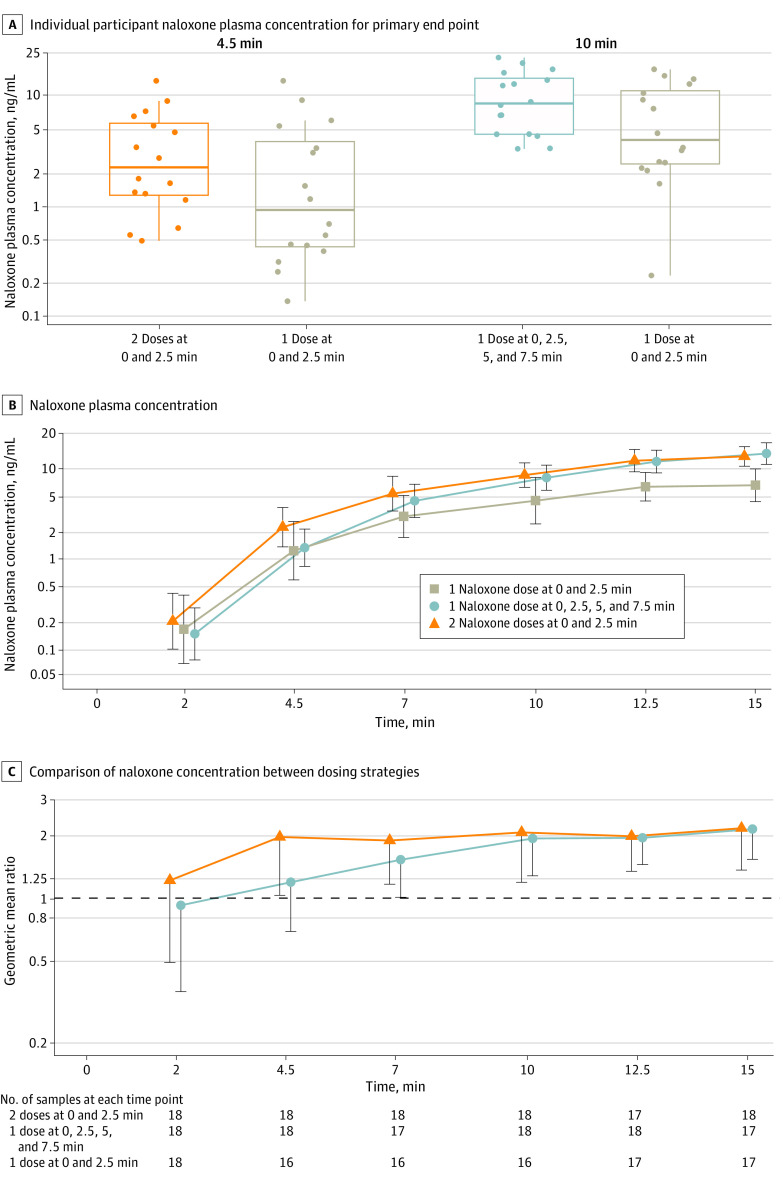
Naloxone Plasma Concentration and Comparisons Between Treatment Groups A, Individual participant observed data and box-and-whisker plot summaries for naloxone plasma concentration. The line through each box represents the median. The lower and upper borders of the box represent the 25th and 75th percentiles, respectively. The whisker extends from the box border to the last observation within 1.5 times the IQR. B, Naloxone plasma concentration. Error bars represent 2-sided 95% CIs. C, Comparison of naloxone plasma concentration between dosing strategies. Error bars represent 1-sided 97.8% CIs. The prespecified times for comparison of 1 dose at 0, 2.5, 5, and 7.5 minutes vs 1 dose at 0 and 2.5 minutes were 10, 12.5, and 15 minutes. The prespecified times for comparison of 2 doses at 0 and 2.5 minutes vs 1 dose at 0 and 2.5 minutes were 4.5, 7, and 10 minutes. eTable 3 in Supplement 2 contains the number of participant samples included at each time for each dosing group.

Figure 2 and eTable 4 in Supplement 2 show naloxone plasma concentration data and comparisons between treatment groups. Administration of 1 naloxone dose at 0, 2.5, 5, and 7.5 minutes, compared with 1 naloxone dose at 0 and 2.5 minutes, significantly increased the geometric mean plasma concentration at 10 minutes (7.95 vs 4.42 ng/mL [coefficient of variation (CV), 72% vs 159%]; geometric mean ratio, 1.95 [1-sided 97.8% CI, 1.28-∞]). Administration of 2 naloxone doses at 0 and 2.5 minutes, compared with 1 naloxone dose at 0 and 2.5 minutes, significantly increased naloxone plasma concentration at 4.5 minutes (2.24 vs 1.23 ng/mL [CV, 134% vs 250%]; geometric mean ratio, 1.98 [1-sided 97.8% CI, 1.03-∞]).

#### Secondary and Exploratory Outcomes: Pharmacokinetics

eTable 5 in Supplement 2 shows the secondary pharmacokinetic outcome comparisons for the two 4-dose groups (geometric mean ratio, 1.69 [1-sided 97.8% CI, 1.06-∞] at 4.5 minutes) and the dose-normalized plasma concentration comparisons. The dose-normalized area under the curve geometric mean ratio for 1 dose at 0, 2.5, 5, and 7.5 minutes compared with 1 dose at 0 and 2.5 minutes was 0.82 (90% CI, 0.75-0.89); the value for 2 doses at 0 and 2.5 minutes compared with 1 dose at 0 and 2.5 minutes was 0.74 (90% CI, 0.69-0.80). eTable 6 in Supplement 2 contains data on the naloxone maximum plasma concentration, time of maximum concentration, and area under the plasma concentration vs time curve.

#### Adverse Events

No serious drug-related adverse events were reported. The most common adverse event was nasal discomfort, which occurred in 13 participants (62%). eTable 7 in Supplement 2 contains a complete list of adverse events.

### Simulated Patients

**Figure 3.**
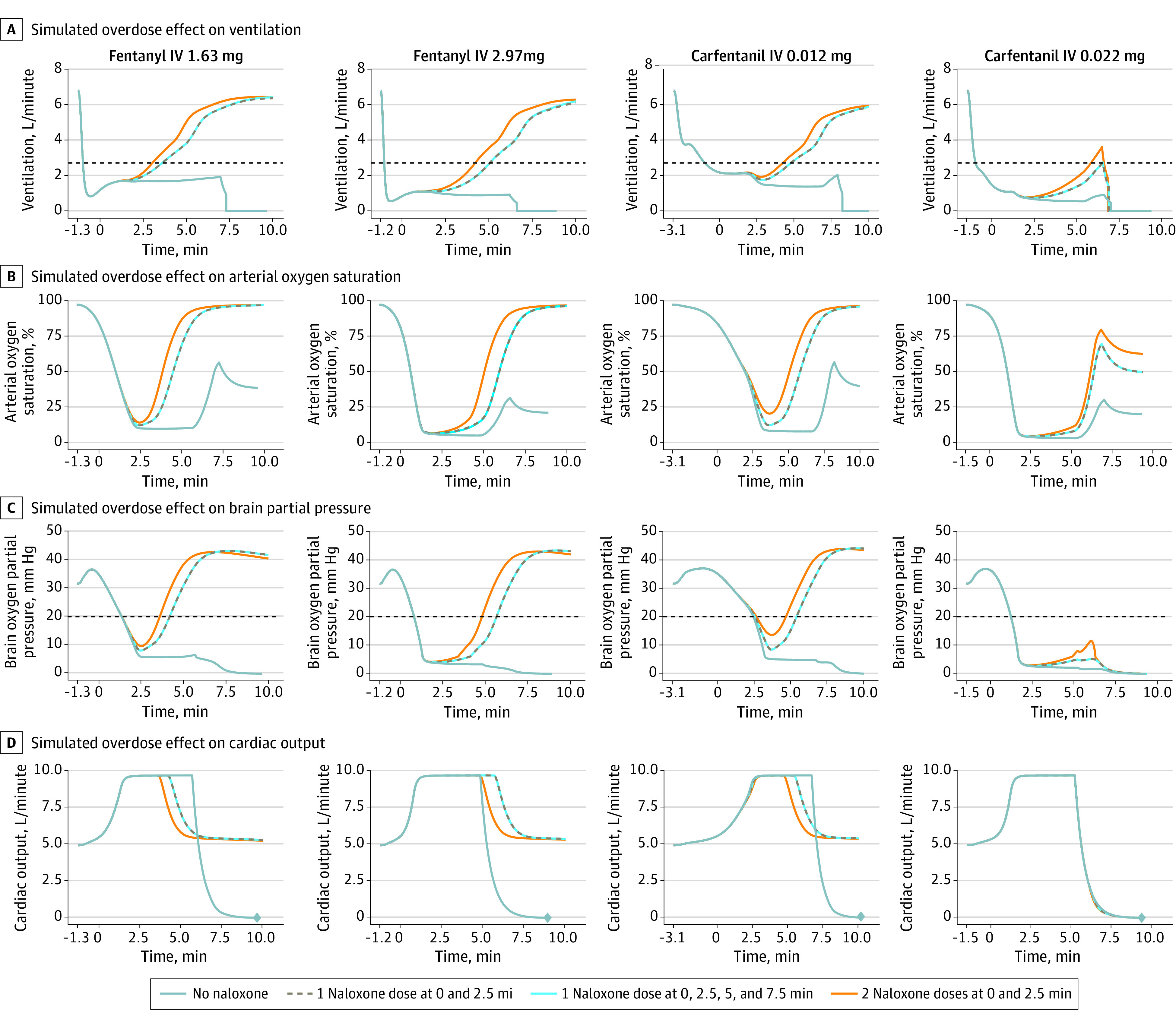
Model-Estimated Effects of Naloxone on Fentanyl and Carfentanil Overdoses A to D, Simulations of the effect of fentanyl and carfentanil overdoses on ventilation (A), arterial oxygen saturation (B), brain oxygen partial pressure (C), and cardiac output (D) for the typical patient. Each graph begins with the time of fentanyl or carfentanil administration. The first dose of intranasal naloxone 4 mg was administered 1 minute after ventilation decreased below 40% of baseline (ie, first naloxone dose at 0 minutes in each graph). With no naloxone, the simulated typical patient experienced cardiac arrest (diamonds in D). In A, the dotted black line is 40% of baseline ventilation. In C, the dotted black line is brain oxygen partial pressure of 20 mm Hg, which was used as an end point in this study. eFigure 1 in Supplement 2 contains similar graphs for the other physiologic outcomes. IV indicates intravenous.

Figure 3 shows simulations of the effect of fentanyl and carfentanil overdoses on ventilation, arterial oxygen saturation, brain oxygen partial pressure, and cardiac output for the typical patient. eFigure 1 in Supplement 2 provides simulations of arterial oxygen, arterial carbon dioxide, and brain blood flow.

#### Secondary Outcome: Brain Hypoxia Time in Simulated Patients

After administration of 2.97 mg of fentanyl, the median brain hypoxia time was infinite minutes (IQR, ∞-∞ minutes) with no naloxone, 4.5 minutes (IQR, 2.1-∞ minutes) with 1 naloxone dose at 0 and 2.5 minutes, 4.5 minutes (IQR, 2.1-∞ minutes) with 1 naloxone dose at 0, 2.5, 5, and 7.5 minutes, and 3.7 minutes (IQR, 1.5-∞ minutes) with 2 naloxone doses at 0 and 2.5 minutes. After administration of 0.022 mg of carfentanil, the median brain hypoxia time was infinite minutes (IQR, ∞-∞ minutes) with no naloxone, infinite minutes (IQR, 4.1-∞ minutes) with 1 naloxone dose at 0 and 2.5 minutes, infinite minutes (IQR, 4.1-∞ minutes) with 1 naloxone dose at 0, 2.5, 5, and 7.5 minutes, and infinite minutes (IQR, 3.3-∞ minutes) with 2 naloxone doses at 0 and 2.5 minutes. The Table contains data for the lower doses of fentanyl and carfentanil.

**Table.** Physiologic Model–Estimated Overdose Outcomes With Different Naloxone Dosing Strategies^a^

Opioid and intranasal naloxone (4 mg/0.1 mL) dosing^b^	Time brain tissue oxygen partial pressure <20 mm Hg, min	Cardiac arrest, %
**Fentanyl, 1.63 mg IV bolus**		
Naloxone doses administered		
0	∞ (0-∞)	52 (50-54)
1 at 0 min	2.2 (0-4.7)	21 (19-23)
1 at 0 and 2.5 min	2.2 (0-4.5)	20 (19-22)
1 at 0, 2.5, 5, and 7.5 min	2.2 (0-4.5)	20 (19-22)
2 at 0 min	1.6 (0-3.8)	14 (12-16)
2 at 0 and 2.5 min	1.6 (0-3.7)	13 (12-15)
**Fentanyl, 2.97 mg IV bolus**		
Naloxone doses administered		
0	∞ (∞-∞)	78 (76-80)
1 at 0 min	4.7 (2.1-∞)	46 (44-49)
1 at 0 and 2.5 min	4.5 (2.1-∞)	46 (44-48)
1 at 0, 2.5, 5, and 7.5 min	4.5 (2.1-∞)	46 (44-48)
2 at 0 min	3.7 (1.5-∞)	35 (32-37)
2 at 0 and 2.5 min	3.7 (1.5-∞)	34 (32-36)
**Carfentanil, 0.012 mg IV bolus**		
Naloxone doses administered		
0	∞ (0-∞)	59 (57-62)
1 at 0 min	2.4 (0-∞)	28 (26-30)
1 at 0 and 2.5 min	2.3 (0-∞)	27 (24-29)
1 at 0, 2.5, 5, and 7.5 min	2.3 (0-∞)	27 (24-29)
2 at 0 min	1 (0-4.5)	20 (19-22)
2 at 0 and 2.5 min	1 (0-4.4)	20 (18-22)
**Carfentanil, 0.022 mg IV bolus**		
Naloxone doses administered		
0	∞ (∞-∞)	90 (89-92)
1 at 0 min	∞ (4.2-∞)	67 (65-70)
1 at 0 and 2.5 min	∞ (4.1-∞)	66 (64-68)
1 at 0, 2.5, 5, and 7.5 min	∞ (4.1-∞)	66 (64-68)
2 at 0 min	∞ (3.3-∞)	55 (53-58)
2 at 0 and 2.5 min	∞ (3.3-∞)	54 (52-57)

Abbreviation: IV, intravenous.

^a^ Values are presented as the median (IQR).

^b^ First naloxone dose administered 1 minute after ventilation decreased below 40% of baseline (Figure 3).

#### Exploratory Outcomes

**Figure 4.**
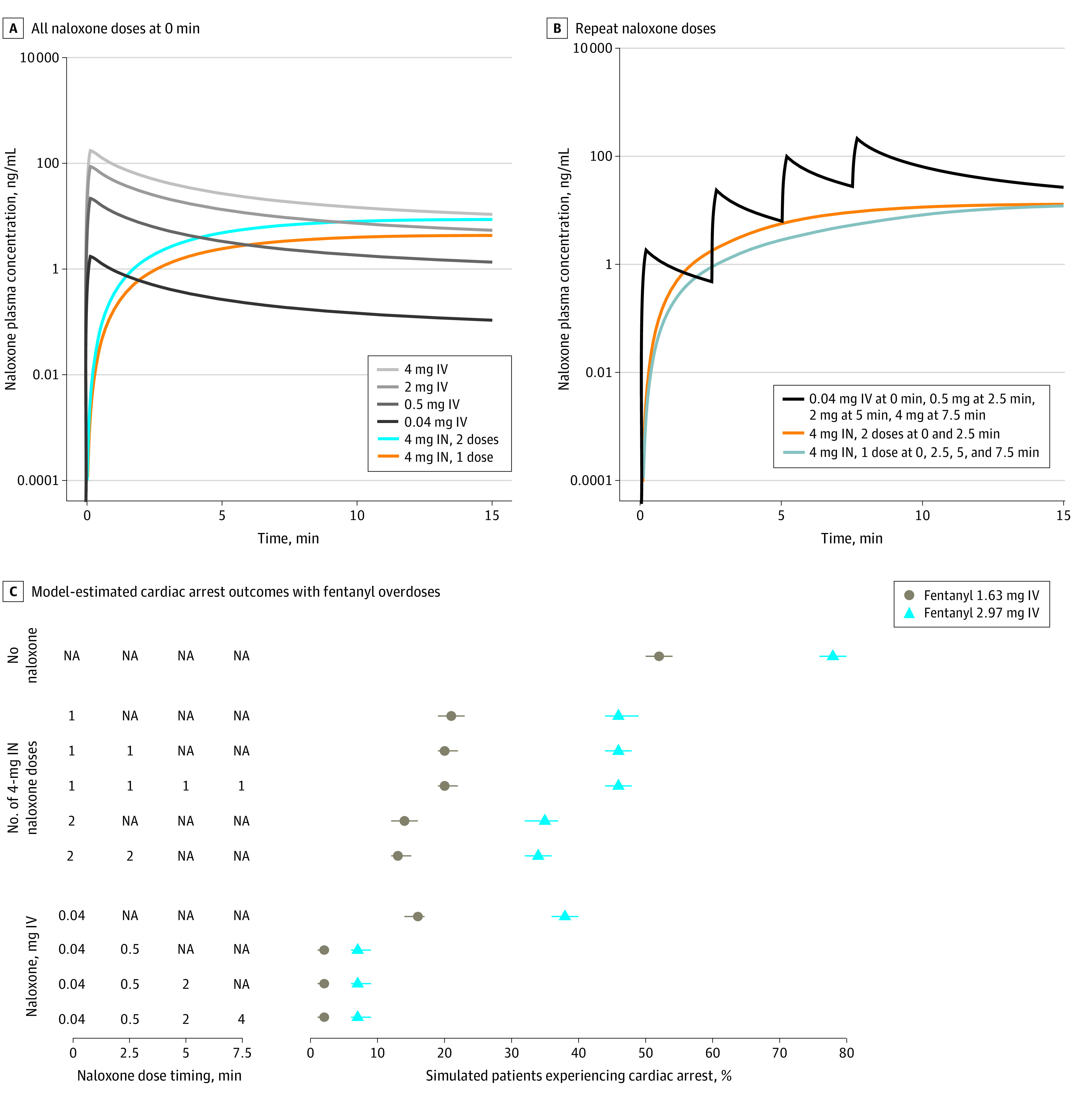
Simulated Intranasal vs Intravenous Naloxone and Model-Estimated Fentanyl Overdose Outcomes A and B, Simulated naloxone plasma concentrations after intranasal (IN) or intravenous (IV) administration where each dose was administered at 0 minutes (A) or with repeat dosing every 2.5 minutes (B). C, Model-estimated percentage of simulated patients experiencing cardiac arrest following fentanyl overdoses with different naloxone dosing. The first naloxone dose was administered 1 minute after ventilation decreased below 40% of baseline. The intravenous naloxone escalating dosing protocol was as described by Boyer^2^ (intravenous naloxone 0.04 mg at 0 minutes, 0.5 mg at 2.5 minutes, 2 mg at 5 minutes, and 4 mg at 7.5 minutes) and is provided for comparative purposes. The intravenous simulations use the model from Papathanasiou et al.^24^ The points and error bars represent the median and IQR of cardiac arrest percentage. eFigure 2 in Supplement 2 contains a similar graph for carfentanil overdoses. NA indicates not applicable.

Patient outcomes for cardiac arrest, arterial oxygen and carbon dioxide, varied delay until naloxone administration, and intravenous vs intranasal naloxone administration were simulated as follows. After administration of 2.97 mg of fentanyl, the percentage of simulated patients experiencing cardiac arrest was 78% (IQR, 76%-80%) with no naloxone, 46% (IQR, 44%-49%) with 1 naloxone dose at 0 minutes, 46% (IQR, 44%-48%) with 1 naloxone dose at 0 and 2.5 minutes, 46% (IQR, 44%-48%) with 1 naloxone dose at 0, 2.5, 5, and 7.5 minutes, 35% (IQR, 32%-37%) with 2 naloxone doses at 0 minutes, and 34% (IQR, 32%-36%) with 2 naloxone doses at 0 and 2.5 minutes. After administration of 0.022 mg of carfentanil, the percentage of simulated patients experiencing cardiac arrest was 90% (IQR, 89%-92%) with no naloxone, 67% (IQR, 65%-70%) with 1 naloxone dose at 0 minutes, 66% (IQR, 64%-68%) with 1 naloxone dose at 0 and 2.5 minutes, 66% (IQR, 64%-68%) with 1 naloxone dose at 0, 2.5, 5, and 7.5 minutes, 55% (IQR, 53%-58%) with 2 naloxone doses at 0 minutes, and 54% (IQR, 52%-57%) with 2 naloxone doses at 0 and 2.5 minutes. The Table, Figure 4, and eFigure 2 in Supplement 2 contain cardiac arrest data for other overdose scenarios. Data on simulations for arterial carbon dioxide and oxygen are presented in eTable 8 in Supplement 2.

After administration of 2.97 mg of fentanyl, when varying the time between ventilation decreasing to 40% of baseline and administering 1 naloxone dose at 0 minutes, the percentage of patients experiencing cardiac arrest was 42% (IQR, 40%-44%) with a 0.5-minute delay, 66% (IQR, 63%-68%) with a 3-minute delay, and 78% (IQR, 76%-80%) with a 10-minute delay. eFigure 3 in Supplement 2 contains data for other overdose scenarios.

Figure 4 shows simulated plasma concentrations after single and repeat dosing protocols for intravenous and intranasal naloxone and cardiac arrest outcomes after fentanyl overdose. (eFigure 2 in Supplement 2 provides details for carfentanil overdose.) After administration of 2.97 mg of fentanyl, the percentage of simulated patients experiencing cardiac arrest was 38% (IQR, 36%-40%) with 0.04 mg of naloxone intravenously at 0 minutes, 7% (IQR, 6%-9%) with 0.04 mg of naloxone at 0 minutes and 0.5 mg at 2.5 minutes, and 7% (IQR, 6%-9%) with 0.04 mg of naloxone at 0 minutes, 0.5 mg at 2.5 minutes, and 2 mg at 5 minutes.

## Discussion

In this randomized crossover trial in healthy participants, compared with administration of 1 intranasal dose of 4 mg of naloxone at 0 and 2.5 minutes, 1 dose at 0, 2.5, 5, and 7.5 minutes significantly increased naloxone plasma concentration at 10 minutes, whereas 2 doses at 0 and 2.5 minutes significantly increased naloxone plasma concentration at 4.5 minutes. Simulations of fentanyl and carfentanil overdoses in a physiologic pharmacokinetic-pharmacodynamic model provided insights into how these different naloxone dosing strategies may affect brain hypoxia time and cardiac arrest in a community setting.

In health care settings with adequate ventilatory support, naloxone can be titrated to reverse an opioid overdose and minimize the risk for precipitating acute withdrawal in opioid-tolerant individuals.^2^ However, in a community setting without ventilatory support, there is a limited window before hypoxic injury is irreversible and cardiac arrest occurs.^11^ Simulations of fentanyl and carfentanil overdoses in this study revealed a pattern of decreasing cardiac arrest percentage with increasing number of intranasal naloxone doses at 0 minutes but not with repeat naloxone dosing every 2.5 minutes. For example, after administration of 2.97 mg of fentanyl, the percentage of simulated patients experiencing cardiac arrest was 46% with 1 dose and 35% with 2 doses at 0 minutes; however, the percentage was 46% regardless of whether 1, 2, or 4 doses were administered when repeating doses every 2.5 minutes (Figure 4 and Table). The mechanism behind this in the simulations is that naloxone must reach sufficient concentration to displace fentanyl from the mu-opioid receptor to increase ventilation and reverse hypoxia prior to cardiovascular decompensation leading to cardiac arrest (Figure 2). Whereas intranasal naloxone reaches maximal plasma concentration after approximately 15 minutes, maximal plasma concentration occurs almost immediately with intravenous naloxone; thus, waiting 2.5 minutes to administer an additional naloxone dose can still allow for sufficient concentration to be reached in time to decrease the cardiac arrest percentage (Figure 4).

At a 2016 FDA advisory committee meeting on community use of naloxone, there was general agreement that the risk of underdosing naloxone far outweighs the potential risk of precipitating opioid withdrawal; however, a consensus could not be reached on certain aspects related to dosing recommendations, due to a lack of evidence.^8^ There are conflicting data in the literature on whether higher or more doses of naloxone are needed in the current era of illicitly manufactured fentanyl.^9,10,25,26,27^ Most studies are single-center retrospective analyses, with limitations such as only including patients who survived an overdose or combining naloxone data from different routes of administration without considering the differences in the time profile of naloxone plasma concentration. Model-based approaches, like those used in this study, have been applied in drug development^28,29,30^ and can help fill information gaps where it is challenging to conduct clinical trials.

Results of this study highlight the importance of early naloxone administration for fentanyl overdose. The FDA is committed to increasing the accessibility of naloxone^31^ and recently approved the first intranasal naloxone product for over-the-counter use,^32^ which was unanimously supported by an FDA advisory committee.^33^ In 2021, the FDA also approved an 8-mg intranasal spray and a 5-mg intramuscular autoinjector as prescription products.^34,35^ The potential benefits of additional or higher naloxone doses should be balanced by potential risks. Naloxone generally has a good safety profile, but it can precipitate withdrawal in patients with opioid dependence,^1,2,36,37^ which is uncomfortable but rarely life-threatening. At the 2016 FDA advisory committee meeting, many committee members stated that the risk of acute withdrawal is acceptable for the benefit of saving a patient.^8^ However, others have separately proposed that higher naloxone doses could decrease the willingness of individuals who use opioids to carry naloxone because of the potential for more severe withdrawal symptoms.^27^ Consideration of multistakeholder feedback is critical, which was the focus of a recent FDA public meeting on understanding fatal overdoses.^38^

### Limitations

This study has several limitations. First, the trial was conducted in a controlled setting with naloxone administered by health care providers; however, the crossover design and standardized procedures allow for comparisons between treatment groups. Second, the trial included healthy participants, not patients with opioid overdose; however, the modeling simulated critical aspects of patients with opioid overdose.^17^ Third, these findings are specific to the naloxone product studied, and the pharmacokinetics of the same dose of naloxone can vary between products based on the route of administration, naloxone concentration, solution volume, and inactive ingredients.^25^ Fourth, the simulations only included 2 overdose scenarios for fentanyl and carfentanil, did not include rescue breathing or chest compressions, and focused on acute recovery (up to 1 hour) without consideration for subsequent potential renarcotization. In addition, simulated fentanyl doses were based on postmortem plasma samples from a study of fatal fentanyl overdoses. The model could be adapted to simulate other doses and scenarios in future work.

## Conclusions

In this randomized clinical trial with healthy participants, compared with 1 intranasal naloxone dose administered at 0 and 2.5 minutes, 1 dose at 0, 2.5, 5, and 7.5 minutes significantly increased naloxone plasma concentration at 10 minutes, whereas 2 doses administered at 0 and 2.5 minutes significantly increased naloxone plasma concentration at 4.5 minutes. Additional research is needed to determine optimal naloxone dosing in the community setting.

## Notes

Supplement 1.
Trial Protocol, Statistical Analysis Plan, and Model Analysis Plan

Supplement 2. **eMethods 1.** Bioanalytical Method Conditions for Naloxone in Human Plasma **eMethods 2.** Modeling **eFigure 1.** Model-Predicted Outcomes of Fentanyl and Carfentanil Overdoses With and Without Naloxone **eFigure 2.** Model-Predicted Cardiac Arrest Outcomes With Carfentanil Overdoses and Intranasal or Intravenous Naloxone **eFigure 3.** Effect of Changing the Delay Between Ventilatory Depression and the First Naloxone Dose **eTable 1.** Exploratory Outcomes **eTable 2.** Study Participant Demographics **eTable 3.** Number of Participant Samples Included in Analyses **eTable 4.** Primary Pharmacokinetic Outcomes **eTable 5.** Secondary Pharmacokinetic Outcomes **eTable 6.** Exploratory Pharmacokinetic Measures by Naloxone Treatment Group **eTable 7.** Incidence and Number of Adverse Events by Treatment Group **eTable 8.** Model-Predicted Rescue Times Based on Different Dosing Scenarios and Respiratory Measures **eAppendix.** Data Dictionary for Naloxone Pharmacokinetic Data Set
eReferences

Supplement 3.
Deidentified Participant Data

Supplement 4.
Data Sharing Statement
