# Supplementary material for: Intranasal Naloxone Repeat Dosing Strategies and Fentanyl Overdose: A Simulation-Based Randomized Clinical Trial
Source: JAMA Netw Open. 2024 Jan 23;7(1):e2351839. doi: 10.1001/jamanetworkopen.2023.51839 (PMC10807299; doi:10.1001/jamanetworkopen.2023.51839)
Supplement: Supplement 1. — Trial Protocol, Statistical Analysis Plan, and Model Analysis Plan [file jamanetwopen-e2351839-s001.pdf]

This supplement contains the following items:

1. Original Protocol (15th February 2021)……………………………………………………1
2. Statistical Analysis Plan (10th March 2021)……………………………………………55
3. Model Analysis Plan (25th October 2021)………………………………………………...73

Please note there was only one version of the Study Protocol, Statistical Analysis Plan, and Model Analysis Plan.

CLINICAL STUDY PROTOCOL

Clinical study to investigate the pharmacokinetics of multiple repeated doses of intranasal naloxone

PROTOCOL NO. SCR-011

Sponsor: U.S. Food and Drug Administration
White Oak Building #64, Room 2072
10903 New Hampshire Avenue
Silver Spring, MD 20993

Sponsor Study Lead: David Strauss, MD, PhD
Director, Division of Applied Regulatory Science
U.S. Food and Drug Administration
Telephone: 301-796-6323
Email: david.strauss@fda.hhs.gov

Sponsor Medical Monitor: Keith Burkhart, MD
U.S. Food and Drug Administration
Telephone: 301-796-2226
Email: keith.burkhart@fda.hhs.gov

Project Manager: Jeffry Florian, PhD
U.S. Food and Drug Administration
Telephone: 301-796-4847
Email: jeffry.florian @fda.hhs.gov

Version of Protocol: 1.0

Date of Protocol: 15 February 2021

CONFIDENTIAL

The concepts and information contained in this document or generated during the study are considered proprietary and may not be disclosed in whole or in part without the expressed written consent of the U.S. Food and Drug Administration.

SPONSOR SIGNATURE PAGE

This study will be conducted with the highest respect for the individual participants in accordance with the requirements of this clinical study protocol and also in accordance with the following:

- The ethical principles that have their origin in the Declaration of Helsinki;
- International Council for Harmonisation (ICH) harmonised tripartite guideline E6 (R2): Good Clinical Practice; and
- All applicable laws and regulations, including without limitation, data privacy laws and compliance with appropriate regulations, including human subject research requirements set forth by the Institutional Review Board (IRB).

Digitally signed by David Strauss -S
DN: c=US, o=U.S. Government, ou=HHS, ou=FDA, ou=People, cn=David Strauss -S, 0.9.2342.19200300.100.1.1=2000507494
Date: 2021.03.03 12:44:51 -05'00'

David Strauss, MD, PhD<br>Director, Division of Applied Regulatory Science<br>U.S. Food and Drug Administration	Date

INVESTIGATOR SIGNATURE PAGE

I confirm that I have read and that I understand this protocol, the investigator brochure, and other product information provided by the sponsor. I agree to conduct this study in accordance with the requirements of this protocol and also protect the rights, safety, privacy, and well-being of study subjects in accordance with the following:

- The ethical principles that have their origin in the Declaration of Helsinki;
- ICH harmonised tripartite guideline E6 (R2): Good Clinical Practice;
- All applicable laws and regulations, including without limitation data privacy laws and regulations;
- Human subject research requirements set forth by the IRB;
- Regulatory requirements for reporting of serious adverse events (SAEs) defined in Section 4.7.2.1 of this protocol; and
- Terms outlined in the Clinical Study Site Agreement.

I further authorize that my personal information may be processed and transferred in accordance with the uses contemplated in Section 6 of this protocol.

Jennifer Deering, MSN, APNP
Principal Investigator

25Feb2021
Date

TABLE OF CONTENTS

PROTOCOL SYNOPSIS **7**

1. INTRODUCTION **18**

2. STUDY OBJECTIVES **19**

3. STUDY ENDPOINTS **19**

3.1 PRIMARY ENDPOINT 19
3.2 SECONDARY ENDPOINTS 19
3.3 EXPLORATORY ENDPOINT 19

4. INVESTIGATIONAL PLAN **19**

4.1 STUDY DESIGN 19
4.1.1 Risk/Benefit *20*
4.2 SELECTION OF STUDY POPULATION 22
4.2.1 Inclusion Criteria *22*
4.2.2 Exclusion Criteria *23*
4.3 SCREENING FAILURES 24
4.4 TERMINATION OF STUDY OR INVESTIGATIONAL SITE 25
4.4.1 Criteria for Termination of the Study *25*
4.4.2 Criteria for Termination of the Investigational Site *25*
4.5 CRITERIA FOR SUBJECT WITHDRAWAL 25
4.5.1 Handling of Withdrawals *26*
4.5.2 Replacement of Subjects *27*
4.6 STUDY VISITS 27
4.6.1 Recruitment *27*
4.6.2 Compensation *27*
4.6.3 Screening *28*
4.6.4 Study Periods *28*
4.6.5 Washout *30*
4.6.6 Discharge (or Early Termination) *30*
4.7 STUDY PROCEDURES 30
4.7.1 Pharmacokinetic Assessments *30*
4.7.2 Safety Assessments *32*
4.7.3 Demographics and Medical History *36*
4.8 STUDY TREATMENTS 36
4.8.1 Treatments Administered and Schedule *36*
4.8.2 Dose Selection *37*
4.8.3 Method of Assigning Subjects to Treatment Sequence *37*
4.8.4 Identity of Study Drug *38*
4.8.5 Management of Clinical Supplies *39*
4.8.6 Blinding *39*
4.8.7 Treatment Compliance *39*
4.8.8 Prior and Concomitant Medications *40*
4.8.9 Subject Restrictions *40*
4.9 STATISTICAL METHODS 41
4.9.1 Sample Size *41*
4.9.1 Analysis Populations *41*
4.9.2 General Statistical Considerations *42*
4.9.3 Subject Disposition *42*

4.9.4 Demographics and Baseline Characteristics *42*
4.9.5 Pharmacokinetic Analysis *42*
4.9.6 PK/PD Modeling *43*
4.9.7 Safety Analyses *43*
4.9.8 Missing Data *44*
4.10 DATA QUALITY ASSURANCE 44
4.11 DATA SHARING 45

5. ETHICAL CONSIDERATIONS 45

5.1 ETHICAL CONDUCT OF THE STUDY 45
5.2 INSTITUTIONAL REVIEW BOARD (IRB) 45

6. ADMINISTRATIVE PROCEDURES 46

6.1 RESPONSIBILITIES OF THE INVESTIGATOR 46
6.1.1 Form FDA 1572 .... *46*
6.1.2 Adherence to Protocol *46*
6.1.3 Reporting Requirements *46*
6.1.4 Source Documentation *46*
6.1.5 Retention of Records *46*
6.1.6 Financial Disclosure and Obligations *47*
6.2 CONFIDENTIALITY AND DISCLOSURE OF DATA 47
6.3 SUBJECT CONSENT 48
6.4 DATA COLLECTION 48
6.5 PUBLICATIONS 48

7. STUDY MANAGEMENT 49

7.1 MEDICAL MONITOR 49
7.2 STUDY MONITORING 49
7.3 MANAGEMENT OF PROTOCOL AMENDMENTS AND DEVIATIONS 49
7.3.1 Modification of the Protocol *50*
7.3.2 Protocol Violations and Deviations *50*

8. REFERENCE LIST 51

9. APPENDICES 52

9.1 APPENDIX A – SCHEDULE OF EVENTS 52
9.2 APPENDIX B – LIST OF ABBREVIATIONS 53
9.3 APPENDIX C – PROTOCOL REVISION HISTORY 54

LIST OF TABLES

TABLE 4-1: STUDY SCHEDULE **20**
TABLE 4-2: STUDY TREATMENTS **20**
TABLE 4-3: CLINICAL LABORATORY TESTS & DIAGNOSTIC SCREENING TESTS **35**
TABLE 4-4: STUDY TREATMENTS & DOSE SCHEDULE **37**
TABLE 9-1: SCHEDULE OF EVENTS **52**

PROTOCOL SYNOPSIS

**Protocol Number:**	SCR-011
**Title:**	Clinical study to investigate the pharmacokinetics of multiple repeated doses of intranasal naloxone
**Investigators:**	Principal Investigator: Jennifer Deering, MSN, APNP<br><br>Study Physician: Carlos Sanabria, MD
**Study Phase:**	1
**Study Period:**	The duration of study participation will be approximately 9 days (excluding the screening period).
**Study Site:**	Spaulding Clinical Research Unit, West Bend, Wisconsin
**Background and Motivation:**	Naloxone, a fast-acting mu-opioid antagonist, is a treatment commonly used in reversing opioid overdose. Naloxone is available in multiple formulations, including for injection intravenously, intramuscularly or subcutaneously, and more recently as a spray administered intranasally (IN). The IN naloxone formulation, which was approved in 2015, is of particular interest as there is a need for naloxone formulations for community use by caregivers and first responders/law enforcement who do not have medical training. It is critical to administer naloxone as quickly as possible to prevent irreversible brain damage and death.<br><br>The U.S. Food and Drug Administration (FDA), Center for Drug Evaluation and Research (CDER), Division of Applied Regulatory Science (DARS) has conducted modeling and simulation to evaluate how many doses of IN naloxone may be needed to reverse opioid-induced respiratory depression from fentanyl and fentanyl derivates under a range of overdose scenarios. These analyses have suggested that more than two doses of IN naloxone would be required to reverse the effects of highly potent opioids (e.g., carfentanil). Experience with intranasal formulations for other products has shown that repeat administration of doses given in close proximity or to the same nostril can influence drug exposure due to run-off from the application site, limited absorption, or other factors. While the pharmacokinetics of IN naloxone have been determined following administration of a 4 mg dose in each nostril concurrently, the pharmacokinetics have not been determined following multiple doses according to the FDA product label:

	• Administer a single spray of NARCAN Nasal Spray to adults or pediatric patients intranasally into one nostril.<br>• Administer additional doses of NARCAN Nasal Spray, using a new nasal spray with each dose, if the patient does not respond or responds and then relapses into respiratory depression, additional doses of NARCAN Nasal Spray may be given every 2 to 3 minutes until emergency medical assistance arrives.<br><br>This involves a 2-3 minute delay between each dose and re-administering to a previously dosed nostril starting with the 3rd dose, which may result in a less than dose-proportional increase in naloxone plasma concentration and/or delayed increase in naloxone exposure compared to the first 2 doses. Obtaining data with repeat dosing will inform if and how fast naloxone plasma concentrations can be reached to be able to reverse highly-potent opioid overdoses.<br><br>This study will be a randomized, unblinded, three-way crossover study to determine naloxone plasma concentration after administration of multiple doses of IN naloxone:<br>A. Four 4 mg IN naloxone doses (1 dose every 2.5 minutes)<br>B. Four 4 mg IN naloxone doses (2 doses every 2.5 minutes)<br>C. Two 4 mg IN naloxone doses (1 dose every 2.5 minutes)<br><br>**IN naloxone (NARCAN Nasal Spray)**<br>Intranasal naloxone is available as a 2 mg or 4 mg dose of naloxone hydrochloride in 0.1 mL with the indication to re-administer additional doses every 2 to 3 minutes (using alternating nostrils) if needed until emergency medical assistance arrives. The 4 mg drug is distributed in packages of two nasal sprays (1 dose per nasal spray), but additional doses can be administered if needed and available.
**Objectives and Endpoints:**	The objectives of this study are:<br>**Objectives:**<br>1. To determine and compare the pharmacokinetics of IN naloxone between the 4 naloxone dose arms and the 2 naloxone dose arm<br>2. To use the pharmacokinetic data from each of the 3 naloxone dosing schedules/doses to predict the time to reverse opioid-induced respiratory depression following different overdose

<table>
<tr><td></td><td>

scenarios based on pharmacokinetic/pharmacodynamic (PK/PD) models

The endpoints for this study are:

Primary Endpoint

1. First timepoint when there is a higher naloxone plasma concentration in the 4 naloxone dose arms compared to the 2 naloxone dose arm

Secondary Endpoints

1. First timepoint when there is a higher naloxone plasma concentration in the 4 naloxone dose arm B (2 doses every 2.5 minutes) compared to the 4 naloxone dose arm A (1 dose every 2.5 minutes)
2. Dose-proportionality of the 4 naloxone dose arms in reference to the 2 naloxone dose arm based on Cmax, $AUC_{0\text{-}inf}$, and $AUC_{0\text{-}t}$
3. Predicted time to rescue a patient from simulated opioid-induced respiratory depression from fentanyl and carfentanil following medium and high overdose scenarios

Exploratory Endpoint

1. Naloxone C_{max}, $AUC_{0\text{-}inf}$, $AUC_{0\text{-}t}$, t_{max}, and partial AUC [pAUC] within the first 30 minutes of dosing

</td></tr>
<tr><td>**Study Design:**</td><td>

This study will be a randomized, unblinded, three-way crossover study to determine naloxone plasma concentration after multiple IN naloxone doses.

Study Schedule:

<table>
<tr><td>Day -1</td><td>Day 1</td><td>Days 2-3</td><td>Day 4</td><td>Days 5-6</td><td>Day 7</td><td>Day 8</td></tr>
<tr><td>Check-in</td><td>Treatment Period 1</td><td>Washout</td><td>Treatment Period 2</td><td>Washout</td><td>Treatment Period 3</td><td>Check-out</td></tr>
</table>

</td></tr>
</table>

	The following 3 treatments will be evaluated in a randomized order over the 3 treatment periods.
	**Study Treatments:**

Treatment	Description
A	Four 4 mg IN naloxone doses (1 dose every 2.5 min; L at 0 min, R at 2.5 min, L at 5 min, R at 7.5 min)
B	Four 4 mg IN naloxone doses (2 doses every 2.5 min; L and R at 0* min, L and R at 2.5* min)
C	Two 4 mg IN naloxone doses (L at 0 min, R at 2.5 min)

*L = Left Nostril, R = Right Nostril, * doses will be ~5 seconds apart (one after the other)*

Healthy subjects will be randomized to one of six treatment sequences (i.e., ABC, ACB, BAC, BCA, CAB, CBA). FDA will prepare the randomization schedule.

Subjects will report to the study site for screening from Days -28 to -2 and then will return to the site on Day -1 for baseline assessments and check-in. After check-in (Day -1), subjects will receive dosing for the 3 respective treatment periods on Days 1, 4 and 7. There will be two days of washout between each treatment period. Participants will be confined in the study clinic from Day -1 until the morning of Day 8. On dosing days, dosing will occur as per the treatment description and PK assessments will occur at the following timepoints:

- PK assessment: 0 (pre-dose), 2, 4.5, 7, 10, 12.5, 15, 20, 30, 45, 60, 120, 180, 240, 360, and 720 minutes

A summary of all assessments prior to and following study drug administration on Day 1, is described in the Schedule of Events (Table 9-1).

Subjects will be discharged from the study after completion of all study procedures. If a subject discontinues from the study prematurely, all procedures scheduled for the end of the study will be performed.

**Subject Population:**	Approximately 20 healthy subjects are planned for enrollment. Every effort will be made to maintain an approximate 50:50 male-to-female sex distribution. Up to 4 subjects may be qualified as replacements. Thus, a maximum of 24 subjects may be exposed to study drug and procedures during the study.

<table>
<tr>
<td>Study and Reference Drugs, Dosage, and Route of Administration:</td>
<td>NARCAN (naloxone hydrochloride) intranasal sprays (4 mg/0.1 mL) will be obtained. On Days 1, 4, and 7 starting at 0 hr subjects will receive 2 or 4 doses of IN naloxone according to the following schedules:
<table>
<tr><th>IN Naloxone Doses</th><th>Schedule</th></tr>
<tr><td>4 doses of 4 mg IN</td><td>L at 0 min, R at 2.5 min, L at 5 min, R at 7.5 min</td></tr>
<tr><td>4 doses of 4 mg IN</td><td>L and R at 0* min, L and R at 2.5* min</td></tr>
<tr><td>2 doses of 4 mg IN</td><td>L at 0 min, R at 2.5 min</td></tr>
</table>
L = Left Nostril, R = Right Nostril, * doses will be ~5 seconds apart (one after the other)</td>
</tr>
<tr>
<td>Inclusion Criteria:</td>
<td>Subjects who meet all the following inclusion criteria will be eligible to participate in the study:

Subject signs an IRB-approved written informed consent and privacy language as per national regulations (e.g., Health Insurance Portability and Accountability Act authorization [HIPAA]) before any study-related procedures are performed.
Subject is a healthy, non-smoking man or woman, 18 to 55 years of age, inclusive, who has a body mass index of 18.5 to 32 kg/m^2, inclusive, at screening.
Subject has normal medical history findings, clinical laboratory results, vital sign measurements, 12 lead ECG (Electrocardiogram) results, and physical examination findings at screening or, if abnormal, the abnormality is not considered clinically significant (as determined and documented by the investigator or designee).
Subject must have a negative test result for alcohol and drugs of abuse at screening and check-in (Day -1).
Subject must test negative for severe acute respiratory syndrome coronavirus (SARS-CoV-2) by a molecular diagnostic test at check-in (Day -1). If a subject’s test comes back inconclusive, it can be repeated.
Female subjects must be of non-childbearing potential or, if they are of childbearing potential, they must: 1) have been strictly abstinent for 1 month before check-in (Day -1) and agree to remain strictly abstinent for the duration of the study and for at least 1 month after the last application of study drug; OR 2) be practicing 2 highly effective methods of birth control (as determined by the investigator or designee; one of the methods must be a barrier technique) from at
</td>
</tr>
</table>

	least 1 month before check-in (Day -1) until at least 1 month after the end of the study.<br>7. Male subjects must agree to practice 2 highly effective methods of birth control (as determined by the investigator or designee) from at least 1 month before check-in (Day -1) until at least 1 month after the last application of study drug.<br>8. Subject is highly likely (as determined by the investigator) to comply with the protocol defined procedures and to complete the study.
**Exclusion Criteria:**	Subjects who meet any of the following exclusion criteria will not be eligible to participate in the study:<br>1. Subject has a deviated septum or previous nasal surgeries or need to use another nasal spray product during study that would impact administration of the intranasal study drug.<br>2. Subject has had an episode of epistaxis or an upper respiratory infection in the previous month.<br>3. Subject has used any prescription or nonprescription drugs (including aspirin or NSAIDs and excluding oral contraceptives and acetaminophen) within 14 days or 5 half-lives (whichever is longer) or complementary and alternative medicines within 28 days before the first dose of study drug.<br>4. Subject is currently participating in another clinical study of an investigational drug or has been dosed with any investigational drug within 30 days or 5 half-lives (whichever is longer) of dosing for this study.<br>5. Subject has used nicotine-containing products (e.g., cigarettes, cigars, chewing tobacco, snuff) within 6 weeks of screening.<br>6. Subject has consumed alcohol, xanthine-containing products (e.g., tea, coffee, cola), caffeine, grapefruit, or grapefruit juice within 24 h of check-in. Subjects must refrain from ingesting these throughout the study.<br>7. Subject has any underlying disease or surgical or medical condition (e.g., cancer, human immunodeficiency virus [HIV], severe hepatic or renal impairment) that could put the subject at risk or would normally prevent participation in a clinical study. This includes subjects with

	any underlying medical conditions that the Investigator believes would put subjects at increased risk of severe illness from COVID-19 based on the Centers for Disease Control and Prevention (CDC) guidelines. The CDC lists cancer, chronic kidney disease, chronic obstructive pulmonary disease, immunocompromised state from solid organ transplant, severe obesity, serious heart conditions, sickle cell disease, pregnancy, smoking and type 2 diabetes mellitus as conditions that put subjects at increased risk. Additionally, the CDC lists asthma (moderate-to-severe), cerebrovascular disease, cystic fibrosis, hypertension, immunocompromised state or immune deficiencies, neurologic conditions such as dementia, liver disease, pulmonary fibrosis, thalassemia, overweight, type 1 diabetes mellitus as conditions that might put subjects at increased risk.<br>8. Subject has any signs or symptoms that are consistent with COVID-19 per CDC recommendations. These include subjects with fever or chills, cough, shortness of breath or difficulty breathing, fatigue, muscle or body aches, headache, new loss of taste or smell, sore throat, congestion or runny nose, nausea or vomiting, or diarrhea may have COVID-19. In addition, the subject has any other findings suggestive of COVID-19 risk in the opinion of the investigator.<br>9. Subject has known or suspected allergies or sensitivities to the study drug.<br>10. Subject has clinical laboratory test results (hematology, serum chemistry and urinalysis) at screening or check-in that are outside the reference ranges provided by the clinical laboratory and considered clinically significant by the investigator.<br>11. Subject has a positive test result at Screening for HIV 1 or 2 antibody, hepatitis C virus antibodies, or hepatitis B surface antigen.<br>12. Subject is unable or unwilling to undergo multiple venipunctures for blood sample collection because of poor tolerability or is unlikely to complete the trial due to poor venous access.<br>13. Female subject is pregnant or lactating before enrollment in the study.

**Sample Collection**	The following samples should be collected and processed as follows. Number of samples collected throughout the study can be found in the Schedule of Events (Table 9-1):<br><br>The **pharmacokinetic blood samples (8 mL each)** will be collected into 10 mL tubes containing K2EDTA, inverted several times to mix the blood with the anticoagulant, and placed in an ice bath. Within 30 minutes of collection, the samples will be centrifuged for 10 minutes, at 3000 revolutions per minute, at 4°C, by a study team member.<br><br>The plasma will be separated using a disposable plastic pipette and approximately half of the plasma will be transferred into duplicate cryotube vials (2 mL polypropylene tubes) labeled as Aliquot A (primary) and Aliquot B (backup). The plasma samples will be appropriately labeled and stored frozen at –70°C or below until shipment. Temperature monitoring logs should be maintained and accessible for review by the study monitor.
**Pharmacokinetic Assessments:**	Samples will be collected as described above and in the Schedule of Events (Table 9-1) for determination of naloxone in blood. The following naloxone PK parameters will be determined for each subject:<br><br>Plasma<br><br>• Concentrations at specified times after the first dose<br>• Maximum concentration (observed peak drug concentration) (Cmax) on the specified day<br>• Partial AUCs (pAUC) over time intervals of 0 to 7 ($pAUC_{0-7}$), 0-10 ($pAUC_{0-10}$), 0-12.5 ($pAUC_{0-12.5}$), 0-15 ($pAUC_{0-15}$), 0-20 ($pAUC_{0-20}$), and 0-30 ($pAUC_{0-30}$) minutes.<br>• AUC from time 0 extrapolated to infinity (AUC_{0-inf})<br>• AUC from time 0 to the sampling time corresponding to the last quantifiable concentration (Clast) (AUC_{0-t})<br>• Time at which C_{max} occurs (t_{max})<br>• Elimination rate constant (K_{el})<br>• Terminal half-life ($t_{1/2}$)<br>• Apparent clearance (CL/F)

**Safety Assessments:**	Safety will be evaluated in terms of adverse events (AEs), clinical laboratory results (hematology, serum chemistry, and urinalysis), vital sign measurements (blood pressure, heart rate, respiratory rate, and oral body temperature), safety 12-lead ECG, and physical examination findings.<br><br>An examination of the nasal passage will be conducted by a trained observer to evaluate evidence of irritation to the nasal mucosa. This nasal irritation assessment will be taken at the following timepoints during study drug administration:<br><br>• 0 (pre-dose), 4.5, 10, 30, 60, 240 minutes
**Sample Size and Threshold Determination:**	Approximately 20 healthy participants are planned for enrollment. The study is intended to obtain information regarding the pharmacokinetics and dose-proportionality of IN naloxone following different dosing schedules. The number of subjects was determined to have greater than 80% power for comparisons between treatment arms across prespecified consecutive timepoints (different timepoints for each comparison). These power calculations are based on 18 subjects with each comparison, allowing for potential dropouts. These analyses adjust for multiplicity using Pocock boundaries for assessing the same endpoint at multiple timepoints. Coefficient of variations and effects sizes for different treatment scenarios were determined using modeling and simulation from previous naloxone studies. Up to 4 subjects may be qualified as replacements, if needed.
**Statistical Methods:**	All data will be presented in data listings. Data from subjects excluded from the PK population will be presented in the data listings but not included in the calculation of summary statistics. The number of subjects who enroll in the study and the number and percentage of subjects who complete each assessment will be presented. The frequency and percentage of subjects who withdraw or discontinue from the study and the reason for withdrawal or discontinuation will be summarized. Demographic and baseline characteristics will be summarized overall and by treatment for all subjects.<br><br>Descriptive statistics will be used to summarize demographic and baseline subject characteristics. For continuous variables, the mean, median, standard deviation (SD), minimum, and maximum values will be reported.

	For categorical (nominal) variables, the number and percentage of subjects (or observations) will be reported.<br><br>The PK population will include all subjects who receive study drug and have at least one on-study sample. The analysis population will include all subjects in the PK population with calculated PK parameters Only subjects with results from two or more periods will be included in comparisons.<br><br>**Pharmacokinetics**: Concentrations will be determined at specified timepoints. The primary endpoint is the first timepoint when there is a higher naloxone plasma concentration in the 4 naloxone dose arms compared to the 2 naloxone dose arm. Secondary endpoints will include determination of the first timepoint when there is a higher naloxone plasma concentration in the 4 naloxone dose arm B (2 doses every 2.5 minutes) compared to the 4 naloxone dose arm A (1 dose every 2.5 minutes). Additional PK measures will be summarized and compared as exploratory endpoints. These include, but are not limited to measures listed under Pharmacokinetic Assessments. Details of the PK analysis will be included in the Statistical Analysis Plan (SAP).<br><br>**Dose Proportionality**: Dose proportionality based on C_{max}, AUC_{0-t}, and AUC_{0-inf} will be compared between each of the three naloxone treatments as secondary endpoints. The 4 naloxone doses arms (separate comparisons for 1 dose every 2.5 minutes and 2 doses every 2.5 minutes) will be compared with the 2 naloxone dose arm (1 dose every 2.5 minutes). An additional comparison will be performed between the 4 naloxone arm B (2 doses every 2.5 minutes) as test and 4 naloxone dose arm A (1 dose every 2.5 minutes) as reference.<br><br>**PK/PD Modeling**: PK data from this study will be used to update the naloxone PK model in a previously developed PK/PD model for opioid-induced respiratory depression. The updated model will be used to predict the mean time to rescue a patient from simulated opioid induced respiratory depression from fentanyl and carfentanil following a range of overdose scenarios (e.g. low, medium and high: to be described in the modeling analysis plan).<br><br>**Safety:** The safety population will include all subjects who receive at least 1 dose of the study drug. All AEs will be coded using the Medical Dictionary for Regulatory Activities (MedDRA). The incidence of AEs,

	organized by system organ class and preferred term, will be summarized with a focus on treatment-emergent AEs. Vital sign measurements will be summarized using descriptive statistics by time point. All values will be evaluated for clinically notable results. Data for additional safety parameters (e.g., physical examination findings) will be listed.
**Date of Protocol:**	15 February 2021

1. INTRODUCTION

Naloxone, a fast-acting mu-opioid antagonist, is a treatment commonly used in reversing opioid overdose. Naloxone is available in multiple formulations, including for injection intravenously, intramuscularly or subcutaneously, and more recently as a spray administered intranasally (IN). The IN naloxone formulation, which was approved in 2015, is of particular interest as there is a need for naloxone formulations for community use by caregivers and first responders/law enforcement who do not have medical training. It is critical to administer naloxone as quickly as possible to prevent irreversible brain damage and death.

The U.S. Food and Drug Administration (FDA), Center for Drug Evaluation and Research (CDER), Division of Applied Regulatory Science (DARS) has conducted modeling and simulation to evaluate how many doses of IN naloxone may be needed to reverse opioid-induced respiratory depression from fentanyl and fentanyl derivates under a range of overdose scenarios. These analyses have suggested that more than two doses of IN naloxone would be required to reverse the effects of highly potent opioids (e.g., carfentanil). Experience with intranasal formulations for other products has shown that repeat administration of doses given in close proximity or to the same nostril can influence drug exposure due to run-off from the application site, limited absorption, or other factors. While the pharmacokinetics of IN naloxone have been determined following administration of a 4 mg dose in each nostril concurrently, the pharmacokinetics have not been determined following multiple doses according to the FDA product label:

- Administer a single spray of NARCAN Nasal Spray to adults or pediatric patients intranasally into one nostril.
- Administer additional doses of NARCAN Nasal Spray, using a new nasal spray with each dose, if the patient does not respond or responds and then relapses into respiratory depression, additional doses of NARCAN Nasal Spray may be given every 2 to 3 minutes until emergency medical assistance arrives.

This involves a 2-3 minute delay between each dose and re-administering to a previously dosed nostril starting with the 3rd dose, which may result in a less than dose-proportional increase in naloxone plasma concentration and/or delayed increase in naloxone exposure compared to the first 2 doses. Obtaining data with repeat dosing will inform if and how fast naloxone plasma concentrations can be reached to be able to reverse highly-potent opioid overdoses.

This study will be a randomized, unblinded, three-way crossover study to determine naloxone plasma concentration after administration of multiple doses of IN naloxone:

A. Four 4 mg IN naloxone doses (1 dose every 2.5 minutes)
B. Four 4 mg IN naloxone doses (2 doses every 2.5 minutes)
C. Two 4 mg IN naloxone doses (1 dose every 2.5 minutes)

IN naloxone (NARCAN Nasal Spray)

Intranasal naloxone is available as a 2 mg or 4 mg dose of naloxone hydrochloride in 0.1 mL with the indication to re-administer additional doses every 2 to 3 minutes (using alternating nostrils) if needed until emergency medical assistance arrives. The 4 mg drug is distributed in packages of two nasal sprays (1 dose per nasal spray), but additional doses can be administered if needed and available.

2. STUDY OBJECTIVES

1. To determine and compare the pharmacokinetics of IN naloxone between the 4 naloxone dose arms and the 2 naloxone dose arm.
2. To use the pharmacokinetic data from each of the 3 naloxone dosing schedules/doses to predict the time to reverse opioid-induced respiratory depression following different overdose scenarios based on pharmacokinetic/pharmacodynamic (PK/PD) models

3. STUDY ENDPOINTS

3.1 Primary Endpoint

1. First timepoint when there is a higher naloxone plasma concentration in the 4 naloxone dose arms compared to the 2 naloxone dose arm.

3.2 Secondary Endpoints

1. First timepoint when there is a higher naloxone plasma concentration in the 4 naloxone dose arm B (2 doses every 2.5 minutes) compared to the 4 naloxone dose arm A (1 dose every 2.5 minutes)
2. Dose-proportionality of the 4 naloxone dose arms in reference to the 2 naloxone dose arm based on C_{max}, $AUC_{0\text{-}inf}$, and $AUC_{0\text{-}t}$
3. Predicted time to rescue a patient from simulated opioid-induced respiratory depression from fentanyl and carfentanil following medium and high overdose scenarios

3.3 Exploratory Endpoint

1. Naloxone C_{max}, $AUC_{0\text{-}inf}$, $AUC_{0\text{-}t}$, t_{max}, and partial AUC [pAUC] within the first 30 minutes of dosing

4. INVESTIGATIONAL PLAN

4.1 Study Design

This study will be a randomized, unblinded, three-way crossover study to determine naloxone plasma concentration after administration of multiple doses of IN naloxone.

Table 4-1: Study Schedule

Day -1	Day 1	Days 2-3	Day 4	Days 5-6	Day 7	Day 8
Check-in	Treatment Period 1	Washout	Treatment Period 2	Washout	Treatment Period 3	Check-out

The following 3 treatments will be evaluated in a randomized order over the 3 treatment periods.

Table 4-2: Study Treatments

Treatment	Description
A	Four 4 mg IN naloxone doses (1 dose every 2.5 min; L at 0 min, R at 2.5 min, L at 5 min, R at 7.5 min)
B	Four 4 mg IN naloxone doses (2 doses every 2.5 min; L and R at 0* min, L and R at 2.5* min)
C	Two 4 mg IN naloxone doses (L at 0 min, R at 2.5 min)

*L = Left Nostril, R = Right Nostril, * doses will be ~5 seconds apart (one after the other)*

Healthy subjects will be randomized to one of six treatment sequences (i.e., ABC, ACB, BAC, BCA, CAB, CBA). FDA will prepare the randomization schedule. Subjects will report to the study site for screening from Days -28 to -2 and then will return to the site on Day -1 for baseline assessments and check-in. After check-in (Day -1), subjects will receive dosing for the 3 respective treatment periods on Days 1, 4 and 7. There will be two days of washout between each treatment period. Participants will be confined in the study clinic from Day -1 until the morning of Day 8. On dosing days, dosing will occur as per the treatment description and PK assessments will occur at the following timepoints:

- PK assessment: 0 (pre-dose), 2, 4.5, 7, 10, 12.5, 15, 20, 30, 45, 60, 120, 180, 240, 360, and 720 minutes

The subject should remain fully supine for approximately one hour post-dose. Subjects should be instructed not to breathe through the nose during administration of the nasal spray into the nose. Subjects will be instructed to leave their masks off for twenty minutes after drug administration.

A summary of all assessments prior to and following study drug administration on Day 1, is described in the Schedule of Events (Table 9-1).

Subjects will be discharged from the study after completion of all study procedures. If a subject discontinues from the study prematurely, all procedures scheduled for the end of the study will be performed.

4.1.1 Risk/Benefit

Subjects will be informed that participation in a human clinical pharmacology study like the present one cannot be of benefit to healthy volunteers. Nevertheless, the information from the physical examination, vital sign measurements, and ECG results may be shared with the subject's personal physician if this is the subject's choice. Subjects will be informed that it is also their choice to inform their personal physician that they are participating in this research study.

Subjects will be informed that their contribution to the study is of major importance to agencies like the FDA to understand how the body processes multiple repeated doses of IN naloxone. However, since this is a study involving healthy volunteers, subjects will be informed that they have the option not to participate.

Subjects will be informed that they may be exposed to risks associated with the pharmacological properties of the study drug and the study procedures. The following summary of potential AEs for the study drug will be provided to and discussed with the subjects:

IN naloxone (NARCAN Nasal Spray Label, dated 08/06/2020)

The following adverse reactions were observed in a NARCAN Nasal Spray clinical study: increased blood pressure, constipation, toothache, muscle spasms, musculoskeletal pain, headache, nasal dryness, nasal edema, nasal congestion, nasal inflammation, rhinalgia, and xeroderma.

Additionally, abrupt reversal of opioid effects in persons who were physically dependent on opioids has precipitated an acute withdrawal syndrome. Signs and symptoms have included: body aches, fever, sweating, runny nose, sneezing, piloerection, yawning, weakness, shivering or trembling, nervousness, restlessness or irritability, diarrhea, nausea or vomiting, abdominal cramps, increased blood pressure, tachycardia. In some patients, there may be aggressive behavior upon abrupt reversal of an opioid overdose. However, opioid-dependent individuals are excluded from this study as subjects cannot use prescription or nonprescription drugs within at least 14 days of study drug administration and a urine drug screen will check for opioids at screening and check-in.

The study drug will not be administered to anyone who is pregnant. All women must take a pregnancy test before receiving any study drug in this study. All women of childbearing potential enrolled on this study will be informed that they must use effective birth control methods (abstinence, intrauterine device, and contraceptive foam and a condom [i.e., double-barrier method]) during treatment. Subjects will be informed that they must notify the investigator if they or their female partners become pregnant during the course of the study. If a subject becomes pregnant, she will be informed that neither Spaulding Clinical Research nor the sponsor will be responsible for the cost of any obstetric or related care, or for the child's care.

Subjects will be informed that insertion of an IV catheter may be required for blood sample collection and, during insertion of the catheter, soreness, bruising, or infection at the insertion

site are possible but unlikely. Subjects will also be informed that dizziness and lightheadedness may occur during direct venipuncture, insertion of the IV catheter, or during blood collection.

Subjects will be informed that the confidentiality of their data will be respected at all times according to state law, and the study personnel handling their study data are bound by confidentiality agreements.

Subjects will be informed that extra precautions will be put in place, including required screening tests, that will limit the risk of COVID-19. All precautions related to COVID-19 will be documented in a COVID-19 Business Continiuity Plan as well as a study-specific COVID-19 Procedure Plan. Subjects will be informed that despite the extra precautions there is still a risk of them contracting COVID-19. Any changes to the COVID-19 precautions (e.g., due to updated CDC recommendations or new testing becoming available) will be documented in the COVID-19 Business Continiuity Plan and study specific COVID-19 Procedure Plan.

Subjects will be informed that the study drug and all tests, procedures, and visits required by the study are provided at no cost to them. If subjects become ill or physically injured because of participation in this study, they will be informed that costs of treatment will not be covered by the sponsor.

4.2 Selection of Study Population

Subjects will be screened, and the data collected will be reviewed by the principal investigator. Only those subjects who meet all the eligibility criteria will be enrolled. Approximately 20 healthy subjects are planned for enrollment. Up to 4 subjects may be qualified as replacements. Thus, a maximum of 24 subjects may be exposed to study drug and procedures during the study. Every effort will be made to maintain an approximate 50:50 male-to-female sex distribution.

4.2.1 Inclusion Criteria

Subjects who meet all of the following inclusion criteria will be eligible to participate in the study:

1. Subject signs an IRB-approved written informed consent and privacy language as per national regulations (e.g., Health Insurance Portability and Accountability Act authorization) before any study-related procedures are performed.
2. Subject is a healthy, non-smoking man or woman, 18 to 55 years of age, inclusive, who has a body mass index of 18.5 to 32 kg/m^2, inclusive, at screening.
3. Subject has normal medical history findings, clinical laboratory results, vital sign measurements, 12 lead ECG results, and physical examination findings at screening or, if abnormal, the abnormality is not considered clinically significant (as determined and documented by the investigator or designee).

4. Subject must have a negative test result for alcohol and drugs of abuse at screening and check-in (Day -1).
5. Subject must test negative for severe acute respiratory syndrome coronavirus (SARS-CoV-2) by a molecular diagnostic test at check-in (Day -1). If a subject's test comes back inconclusive, it can be repeated.
6. Female subjects must be of non-childbearing potential or, if they are of childbearing potential, they must: 1) have been strictly abstinent for 1 month before check-in (Day -1) and agree to remain strictly abstinent for the duration of the study and for at least 1month after the last application of study drug; OR 2) be practicing 2 highly effective methods of birth control (as determined by the investigator or designee; one of the methods must be a barrier technique) from at least 1 month before check-in (Day -1) until at least 1 month after the end of the study.
7. Male subjects must agree to practice 2 highly effective method of birth control (as determined by the investigator or designee) from at least 1 month before check-in (Day -1) until at least 1 month after the last application of study drug.
8. Subject is highly likely (as determined by the investigator) to comply with the protocol defined procedures and to complete the study.

4.2.2 Exclusion Criteria

Subjects who meet any of the following exclusion criteria will not be eligible to participate in the study:

1. Subject has a deviated septum or previous nasal surgeries or need to use another nasal spray product during study that would impact administration of the intranasal study drug.
2. Subject has had an episode of epistaxis or an upper respiratory infection in the previous month.
3. Subject has used any prescription or nonprescription drugs (including aspirin or NSAIDs and excluding oral contraceptives and acetaminophen) within 14 days or 5 half-lives (whichever is longer) or complementary and alternative medicines within 28 days before the first dose of study drug.
4. Subject is currently participating in another clinical study of an investigational drug or has been dosed with any investigational drug within 30 days or 5 half-lives (whichever is longer) of dosing for this study.
5. Subject has used nicotine-containing products (e.g., cigarettes, cigars, chewing tobacco, snuff) within 6 weeks of screening.

6. Subject has consumed alcohol, xanthine-containing products (e.g., tea, coffee, cola), caffeine, grapefruit, or grapefruit juice within 24 h of check-in. Subjects must refrain from ingesting these throughout the study.

7. Subject has any underlying disease or surgical or medical condition (e.g., cancer, human immunodeficiency virus [HIV], severe hepatic or renal impairment) that could put the subject at risk or would normally prevent participation in a clinical study. This includes subjects with any underlying medical conditions that the Investigator believes would put subjects at increased risk of severe illness from COVID-19 based on the Centers for Disease Control and Prevention (CDC) guidelines. The CDC lists cancer, chronic kidney disease, chronic obstructive pulmonary disease, immunocompromised state from solid organ transplant, severe obesity, serious heart conditions, sickle cell disease, pregnancy, smoking and type 2 diabetes mellitus as conditions that put subjects at increased risk. Additionally, the CDC lists asthma (moderate-to-severe), cerebrovascular disease, cystic fibrosis, hypertension, immunocompromised state or immune deficiencies, neurologic conditions such as dementia, liver disease, pulmonary fibrosis, thalassemia, overweight, type 1 diabetes mellitus as conditions that might put subjects at increased risk.

8. Subject has any signs or symptoms that are consistent with COVID-19 per CDC recommendations. These include subjects with fever or chills, cough, shortness of breath or difficulty breathing, fatigue, muscle or body aches, headache, new loss of taste or smell, sore throat, congestion or runny nose, nausea or vomiting, or diarrhea may have COVID-19. In addition, the subject has any other findings suggestive of COVID-19 risk in the opinion of the investigator.

9. Subject has known or suspected allergies or sensitivities to the study drug.

10. Subject has clinical laboratory test results (hematology, serum chemistry and urinalysis) at screening or check-in that are outside the reference ranges provided by the clinical laboratory and considered clinically significant by the investigator.

11. Subject has a positive test result at Screening for HIV 1 or 2 antibody, hepatitis C virus antibodies, or hepatitis B surface antigen.

12. Subject is unable or unwilling to undergo multiple venipunctures for blood sample collection because of poor tolerability or is unlikely to complete the trial due to poor venous access.

13. Female subject is pregnant or lactating before enrollment in the study.

4.3 Screening Failures

Subjects who sign and date the informed consent form but who fail to meet the inclusion and exclusion criteria are defined as screening failures. A screening log, which documents the subject initials and reason(s) for screening failure, will be maintained by the investigator for all screening failures. A copy of the log should be retained in the investigator's study files.

If a subject fails the screening process because of an abnormal laboratory result, they can receive a copy of the results upon request. The investigator will determine if follow-up for the abnormal laboratory result is needed and will encourage the subject to follow-up with his or her personal physician as appropriate. All subjects will be informed as to the reason(s) they are excluded from study participation, even if follow-up is not required. If a subject fails the screening process because of a positive test result for human immunodeficiency virus or hepatitis, the positive result will be reported to local health authorities as required by law.

4.4 Termination of Study or Investigational Site

4.4.1 Criteria for Termination of the Study

The study will be completed as planned unless one of the following criteria is satisfied that requires early termination of the study.

- New information regarding the safety or efficacy of the study drug that indicates a change in the known risk profile for the study drug, such that the risk is no longer acceptable for subjects participating in the study.
- Significant violation of Good Clinical Practice (GCP) that compromises the ability to achieve the primary study objective or compromises subject safety.

4.4.2 Criteria for Termination of the Investigational Site

The study site may be terminated if the site (including the investigator) is found in significant violation of GCP, the protocol, the contractual agreement, or is unable to ensure adequate performance of the study.

In the event that the sponsor elects to terminate the study or the investigational site, a study-specific procedure for early termination will be provided by the sponsor; the procedure will be followed by the applicable investigational site during the course of termination.

4.5 Criteria for Subject Withdrawal

Subjects may withdraw from the study at any time at their own request, or they may be withdrawn by the investigator without the approval of the subject based on the investigator's clinical judgment. A subject is not required to provide a written request to withdraw from the study; however, a written request is required if a subject withdraws consent for his or her personal data to be used for study-related purposes.

A subject may be discontinued for any of the following reasons:

- AE: The subject has experienced an AE that, in the opinion of the investigator, requires early termination. The appropriate electronic case report form (eCRF) must be completed for each AE. If a subject is discontinued from the study due to an AE, the investigator is required to follow-up with the subject until the event resolves or becomes stable. If a subject dies during the study, the cause of death must be reported as a serious AE (SAE), with an outcome of death noted in the eCRF.
- Protocol Violation: The subject failed to meet protocol entry criteria or did not adhere to protocol requirements, and continued participation poses an unnecessary risk to the subject's health.
- Withdrawal by Subject: The subject (or other responsible individual [e.g., caregiver]) wishes to withdraw from the study in the absence of a medical need.

  NOTE: Withdrawal due to an AE should not be recorded in the "voluntary withdrawal" category.
- Study Terminated by Sponsor: The sponsor, IRB, FDA, or other regulatory agency terminates the study.
- Pregnancy: The subject is found to be pregnant.

  NOTE: If the subject is found to be pregnant, the subject must be withdrawn immediately. The pregnancy will be followed-up to term, and the outcome, including any premature termination will be recorded. All live births must be followed for a minimum of 30 days or until the first well-baby visit.
- Other.

  NOTE: This category records withdrawals caused by an accidental or a medical emergency, unblinding, and other rare cases. The specific reason should be recorded in the comment space of the eCRF.

4.5.1 Handling of Withdrawals

The investigator may terminate a subject's study participation at any time during the study when the subject meets the criteria described in Section 4.5. In addition, a subject may discontinue his or her participation without giving a reason at any time during the study. Subjects will be informed that their participation in the study is voluntary, that refusal to participate will involve no penalty or loss of benefits to which the subject is otherwise entitled, and that the subject may discontinue participation at any time without penalty or loss of benefits to which the subject is otherwise entitled.

Should a subject's participation be discontinued, the primary reason for termination must be recorded. In addition, efforts should be made to perform all procedures scheduled for the early termination visit. Any data and samples collected before subject withdrawal will become the property of the sponsor.

If a subject sneezes after dosing, a blood sample will be collected and, if the subject sneezes immediately after dosing, the sponsor will be consulted regarding a decision to continue the subject in the study. Under no circumstances will a dose of the study drug be repeated.

4.5.2 Replacement of Subjects

Approximately 20 healthy subjects are planned for enrollment and will be randomized to one of the six treatment sequences. Up to 4 subjects may be qualified as replacements and replacement subjects (if needed) must complete the treatment period. A replacement subject will receive the same treatment as the subject being replaced. Thus, a maximum of 24 subjects will be exposed to study drug and procedures during the study.

4.6 Study Visits

4.6.1 Recruitment

Recruitment materials (e.g., internet, radio, and print advertisements, social media posts) will be approved by the local IRB before telephone screening. The sponsor is responsible for registration of the study on clinicaltrials.gov. Recruitment (including telephone screening) may not occur until the study is fully registered on clinicaltrials.gov.

4.6.2 Compensation

Subjects will be offered payment for Screening; however, if the results of their alcohol and drug screening tests are positive, they will not be compensated. Subjects who complete the entire study will receive payment according to the schedule provided in the informed consent form. No special incentives are offered. Final payment will not be released until all follow-up procedures have been completed and accepted by the investigator.

If a subject chooses to withdraw from the study prematurely, he or she will only be compensated for completed days. If subjects are withdrawn for medical reasons or if the study is halted temporarily or permanently, the subjects will receive compensation proportional to the time spent in the study. No compensation will be provided if a subject is dismissed from the study for noncompliance (e.g., improper conduct, ingesting alcohol and/or drugs [including recreational drugs], tampering with the study drug, consuming any prohibited foods or beverages).

If subjects are required to stay in the clinic for a longer period for safety reasons, they will be compensated at a rate proportional to the entire compensation for the study. If a subject becomes

ill or physically injured because of participation in this study, the subject will be referred for treatment.

4.6.3 Screening

The following procedures and assessments will be performed at Screening (Days -28 to -2):

- Obtain informed consent/HIPAA authorization. The informed consent process will be performed by a clinical research nurse in a private room. The subject will be given unlimited time to ask questions regarding study participation, and each subject will be questioned to ensure their understanding.

After informed consent is obtained:

- Review inclusion/exclusion criteria to confirm subject eligibility
- Record demographic information
- Measure height, weight, and calculate body mass index
- Perform serology screening (HIV antigen/antibody [Ag/Ab] Combo 1/2, HepC antibody, HBsAg)
- Record medical history
- Perform alcohol and drug screening (amphetamines, barbiturates, benzodiazepines, cannabinoids, cocaine, alcohol, opiates, phencyclidine, propoxyphene, and methadone)
- Perform a serum pregnancy test (female subjects only)
- Perform FSH measuring (postmenopausal [i.e., without menses for two years] female subjects only)
- Record prior medications
- Monitor for AEs
- Perform clinical laboratory tests (hematology, serum chemistry, and urinalysis)
- Measure vital signs (blood pressure, heart rate, respiratory rate, and oral body temperature)
- Perform a safety 12-lead ECG
- Perform a complete physical examination

4.6.4 Study Periods

This is a crossover study with three treatment periods. Subjects will be kept in the study clinic between these three periods. Subjects will check-in on Day -1 and will check-out on the morning of Day 8.

4.6.4.1 Check-In

The following procedures and assessments will be performed at Check-in (Day -1):

- Perform/review results from SARS-CoV-2 molecular test (may be performed ~2 days before check-in to allow time for results)
- Review inclusion/exclusion criteria to confirm subject eligibility
- Measure weight
- Review medical history
- Perform alcohol and drug screening (amphetamines, barbiturates, benzodiazepines, cannabinoids, cocaine, alcohol, opiates, phencyclidine, propoxyphene, and methadone)
- Perform a serum pregnancy test (female subjects only)
- Admit subject to the study clinic
- Randomization (after completion of check-in procedures on Day -1 or just before dosing on Day 1)
- Record concomitant medications
- Monitor for AEs
- Perform clinical laboratory tests (hematology, serum chemistry, and urinalysis)
- Measure vital signs (blood pressure, heart rate, respiratory rate, and oral body temperature)
- Perform a safety 12-lead ECG
- Perform a comprehensive physical examination

4.6.4.2 Treatment

The following procedures and assessments will be performed during the treatment period according to the Schedule of Events (Table 9-1):

- Monitor for AEs
- Record concomitant medications
- Perform a safety 12-lead ECG at 0 (pre-dose), 1h, and 6h timepoints. If scheduled for the same time, safety 12-lead ECGs will always be performed before vital sign measurement and blood sample collection
- Measure vital signs at 0 (pre-dose), 0.5h, 1h, and 6h timepoints (blood pressure, heart rate, respiratory rate, and oral body temperature). If vital signs are scheduled at the same time as another event, vital signs will be measured after the ECG, but before blood sample collection

- Administer study drug according to the randomization schedule following all other pre-dose examinations and specimen collection
- Collect PK blood samples (8 mL) at the timepoints specified in Section 4.1
- Perform nasal irritation assessment at 0 (pre-dose), 4.5, 10, 30, 60 and 240 minutes as described in Section 4.7.2.6

4.6.5 Washout

There will be two washout days (72h from dose to dose) between the treatment periods. The following procedures and assessments will be performed during washout period:

- Monitor for AEs
- Measure vital signs (blood pressure, heart rate, respiratory rate, and oral body temperature)

4.6.6 Discharge (or Early Termination)

The following procedures and assessments will be performed before the subject is discharged from the study or at early termination:

- Perform a serum pregnancy test (female subjects only)
- Record concomitant medications
- Monitor for AEs
- Perform alcohol and drug screening (amphetamines, barbiturates, benzodiazepines, cannabinoids, cocaine, alcohol, opiates, phencyclidine, propoxyphene, and methadone)
- Perform clinical laboratory tests (hematology, serum chemistry, and urinalysis)
- Measure vital signs (blood pressure, heart rate, respiratory rate, and oral body temperature)
- Perform a safety 12-lead ECG
- Perform a complete physical examination
- Measure weight
- Discharge subject from the study clinic after completion of all study procedures

4.7 Study Procedures

4.7.1 Pharmacokinetic Assessments

4.7.1.1 Pharmacokinetic Sample Collection

For this study, PK blood samples will be collected on Day 1 of each treatment period (i.e., dosing days). Blood samples will be collected by direct venipuncture or by inserting an IV catheter into the subject's forearm region. A single PK blood sample (8 mL) will be collected at the following timepoints within each period: 0 (i.e., immediately before dosing), 2, 4.5, 7, 10, 12.5, 15, 20, 30, 45, 60, 120, 180, 240, 360, and 720 minutes after dosing on Days 1, 4 and 7 (Table 9-1). The target time of the PK blood collection is considered the most critical. If the collection time is more than ± 0.5 minute from the scheduled time for the first 15 minutes of collections or more than ± 2 minutes from scheduled timepoints, thereafter, this will be considered a protocol deviation.

Each blood sample will be labeled with subject number, study number, study day, time point, event, and a barcode that matches that belonging to the subject.

4.7.1.2 Specimen Handling

The PK blood samples (8 mL each) will be collected into 10 mL tubes containing K2EDTA, inverted several times to mix the blood with the anticoagulant, and placed in an ice bath. Within 30 minutes of collection, the samples will be centrifuged for 10 minutes, at 3000 revolutions per minute, at 4°C, by a study team member.

The plasma will be separated using a disposable plastic pipette and approximately half of the plasma will be transferred into duplicate cryotube vials (2 mL polypropylene tubes) labeled as Aliquot A (primary) and Aliquot B (backup). The plasma samples will be appropriately labeled and stored frozen at –70°C or below until shipment. Temperature monitoring logs should be maintained and accessible for review by the study monitor.

The Aliquot A samples (primary) will be shipped first, on dry ice, to the bioanalytical laboratory specified by the sponsor for processing when requested by the sponsor. The Aliquot B samples (backup) will be held for a second shipment at a time after the completion of all Aliquot A sample shipment(s) and the timing of the Aliquot B shipment communicated by the sponsor. None of the PK blood samples will be stored at clinical facility for future use. De-identified samples may be stored by the sponsor for additional exploratory analyses.

Plasma concentrations of naloxone will be assayed using a validated liquid chromatography with tandem mass spectrometry method.

4.7.1.3 Pharmacokinetic Parameters

The following PK parameters will be determined for each subject:

Plasma

- Concentrations at specified times after the first dose
- Maximum concentration (observed peak drug concentration) (C_{max}) on the specified day

- AUC from time 0 extrapolated to infinity ($AUC0_{-inf}$)
- AUC from time 0 to the sampling time corresponding to the last quantifiable concentration (Clast) (AUC_{0-t})
- Partial AUCs (pAUC) over time intervals of 0 to 7 ($pAUC_{0-7}$), 0-10 ($pAUC_{0-10}$), 0-12.5 ($pAUC_{0-12.5}$), 0-15 ($pAUC_{0-15}$), 0-20 ($pAUC_{0-20}$), and 0-30 ($pAUC_{0-30}$) minutes.
- Time at which C_{max} occurs (t_{max})
- Elimination rate constant (K_{el})
- Terminal half-life ($t_{1/2}$)
- Apparent clearance (CL/F)

4.7.2 Safety Assessments

Safety will be evaluated in terms of AEs, clinical laboratory results (hematology, serum chemistry, and urinalysis), vital sign measurements (blood pressure, heart rate, respiratory rate, and oral body temperature), safety 12-lead ECG results, and physical examination findings.

An examination of the nasal passage will be conducted by a trained observer to evaluate evidence of irritation to the nasal mucosa. This nasal irritation assessment will be taken at the following timepoints during study drug administration 0 (pre-dose), 4.5, 10, 30, 60, 240 minutes.

4.7.2.1 Adverse Events

4.7.2.1.1 Adverse Event Definitions

An AE is defined as any untoward and/or unintended sign, including an abnormal clinical laboratory finding, symptom, or disease temporally associated with the use of a study drug, whether or not considered related to the study drug. Events or conditions that increase in frequency or severity during or as a consequence of use of a drug in human clinical trials will also be considered AEs.

A treatment-emergent adverse event (TEAE) is defined as an AE that begins after study drug administration.

An unexpected AE is any AE having a specificity or severity not consistent with the current investigator's brochure for the study drug(s).

A serious adverse event (SAE) is defined as any AE occurring at any dose that meets the following criteria:

- Results in death,
- Is life threatening,

- Requires hospitalization or prolongation of existing hospitalization,
- Results in persistent or significant disability or incapacity,
- Results in a congenital anomaly/birth defect due to exposure prior to conception or during pregnancy, or
- Is an important medical event that may not meet the previous criteria but, based upon appropriate medical judgment, jeopardizes the subject or requires medical or surgical intervention to prevent one of the outcomes listed previously.

4.7.2.1.2 Adverse Event Reporting

The recording of AEs will begin after the subject signs the informed consent form and will continue until discharge (or early termination). All AEs, whether serious or nonserious and whether or not related to the study drug, must be recorded in the eCRF. Study subjects will be instructed to warn study staff if he or she has any unexpected symptoms. In addition, all subjects will receive a reminder telephone call approximately 24 h before check-in.

Any SAE (whether expected or unexpected) must be entered into the eCRF system and reported by email to the sponsor or designee using the SAE Reporting Form within 24 h of the investigator or study clinic staff becoming aware of the event. It is the responsibility of the investigator to report all SAEs to the sponsor, to provide the most complete report possible, and to assess each SAE for its relationship to the study drug. The investigator is responsible for obtaining follow-up information on all SAEs and submitting follow-up SAE data. Any unexpected SAEs must be reported promptly to the investigator's IRB as per the IRB's requirements.

In the event of a fatal or life-threatening SAE, the sponsor will notify the appropriate FDA authorities within 7 calendar days of receipt of the report. The sponsor will follow all 7-day alert reports with a written report within 10 working days of receipt of the case. Serious AE cases that concern nonfatal, nonlife-threatening events that are unexpected and at least possibly related to the study drug will be submitted in writing to the FDA within 10 working days of receipt.

Furthermore, any AEs that are not expected, occur at a higher frequency, or would require modification of the study protocol and/or informed consent must be reported to the FDA within 10 working days.

AEs that are assessed by the investigator as possibly or probably related to the study drug will be followed until they resolve or stabilize. All SAEs will be followed until resolution.

4.7.2.1.3 Assessment of Severity

The investigator will assess the severity of each AE using the following scale:

- Mild: The subject is aware of the AE but is still able to perform all activities; minimal or no medical intervention or therapy is required.
- Moderate: The subject has to discontinue some activities due to the AE; minimal or no medical intervention or therapy is required.
- Severe: The subject is incapacitated by the AE and is unable to perform normal activities; significant medical intervention or therapy is required, and hospitalization is possible.

4.7.2.1.4 Assessment of Causality

The investigator will assess the causal relationship/relatedness of each AE to the study drug using the following scale:

- Not Related: Onset of the AE has no reasonable temporal relationship to administration of the study drug, a causal relationship to administration of the study drug is biologically implausible, or the event is attributed to an alternative etiology.
- Unlikely Related: Onset of the AE has a reasonable temporal relationship to study drug administration and although a causal relationship is unlikely, it is biologically plausible.
- Possibly Related: Onset of the AE has a strong temporal relationship to administration of the study drug, cannot be explained by the subject's clinical state or other factors, and a causal relationship is biologically plausible.
- Probably Related: Onset of the AE shows a distinct temporal relationship to administration of the study drug that cannot be explained by the subject's clinical state or other factors, the AE is a known reaction to the product or chemical group, or can be predicted by the product's pharmacology.

4.7.2.1.5 Pregnancy

A serum pregnancy test will be performed for female subjects at the timepoints presented in the Schedule of Events (Table 9-1). If a subject becomes pregnant while on the study, this should be reported immediately to the investigator and sponsor and the subject will be withdrawn from the study. The subject will be instructed to follow-up with his or her personal physician. All pregnancies are to be reported as an AE and followed for outcome.

4.7.2.2 Clinical Laboratory Tests

Clinical laboratory and diagnostic screening tests will be performed at the timepoints presented in the Schedule of Events (Table 9-1) and will be collected in accordance with acceptable laboratory procedures. Clinical laboratory testing will be performed by the clinical study contractor(s). The clinical laboratory tests that will be performed are presented in Table 4-3. Unused clinical laboratory test samples will not be stored for future use.

Table 4-3: Clinical Laboratory Tests & Diagnostic Screening Tests

Hematology	Serum Chemistry	Urinalysis
Hematocrit<br>Hemoglobin<br>Platelet count<br>Red blood cell count<br>White blood cell count (with automated differential)	Alanine aminotransferase<br>Albumin<br>Alkaline phosphatase<br>Aspartate aminotransferase<br>Bicarbonate<br>Bilirubin (total, direct, and indirect)<br>Blood urea nitrogen<br>Calcium<br>Chloride<br>Creatinine (including calculated creatinine clearance – Cockcroft Gault)<br>Glucose<br>Lactate dehydrogenase<br>Magnesium<br>Phosphorus<br>Potassium<br>Sodium<br>Total protein<br>Uric acid	Appearance<br>Bilirubin<br>Blood<br>Color<br>Glucose<br>Ketones<br>Leukocyte esterase<br>Microscopic examination: red blood cells, white blood cells, epithelial cells, bacteria, crystals, and casts (if present)<br>Nitrite<br>pH<br>Protein<br>Specific gravity<br>Urobilinogen
**Diagnostic Screening Tests:**		
**Serum**	**Urine**	**Other**
Serology (human immunodeficiency virus Ag/Ab Combo 1/2, hepatitis C virus antibody, and hepatitis B surface antigen)<br>**Female Subjects Only**<br>Human chorionic gonadotropin (for pregnancy)	Drug screen including: amphetamines, barbiturates, benzodiazepines, cannabinoids, cocaine, alcohol, opiates, phencyclidine, propoxyphene, and methadone	SARS-CoV2 molecular test

4.7.2.3 Vital Sign Measurements

Vital signs (blood pressure, heart rate, respiratory rate, and oral body temperature) will be measured using an automated device on days presented in the Schedule of Events (Table 9-1). The subject should be in a supine position, if possible, for a minimum of 5 minutes before vital signs are measured.

4.7.2.4 Safety 12-lead Electrocardiograms

12-lead ECGs will be obtained with the subjects in the supine position for a minimum of 5 minutes before recording. ECGs will be overread by a physician. If an abnormality is observed, the subject will be instructed to follow-up with his or her personal physician.

4.7.2.5 Physical Examinations

A complete physical examination will be performed on the days presented in the Schedule of Events (Table 9-1).

The complete physical examination will include, but not be limited to, assessments of the head, eyes, ears, nose, throat, skin, thyroid, nervous system, respiratory system, cardiovascular system, abdomen (liver and spleen), lymph nodes, and extremities. Height, weight (without shoes and wearing the lightest possible clothing), and calculation of body mass index will be performed at Screening and check-out.

If a clinically significant abnormality is observed upon physical examination, the subject will be instructed to follow-up with his or her personal physician.

4.7.2.6 Nasal Irritation Assessment

Nasal irritation (erythema, edema, and erosion) will be evaluated by a trained observer during study drug administration at the following timepoints: 0 (pre-dose), 4.5, 10, 30, 60 and 240 minutes. If a PK sample corresponds to the nasal irritation assessment the nasal assessment will be performed after the PK sample is obtained.

Nasal Irritation Scale

1 - Inflamed mucosa, no bleeding

2 - Minor bleeding which stops within 1 minute

3 - Minor bleeding, taking 1-5 minutes to stop

4 - Substantial bleeding for 4-60 minutes, does not require medical intervention

5 - Ulcerated lesions, bleeding which requires medical intervention

4.7.3 Demographics and Medical History

Demographic data (date of birth, gender, race, and ethnicity) will be collected at Screening. Each subject will provide a complete medical history at Screening that will be reviewed at check-in. Specific information relating to any prior or existing medical conditions/surgical procedures will be recorded in the subject’s eCRF.

4.8 Study Treatments

4.8.1 Treatments Administered and Schedule

Subjects will receive each of the following treatments (Table 4-4) according to the randomization schedule. Drug administration will occur on Day 1, 4 and 7 with a 2 day washout period in between.

Table 4-4: Study Treatments and Dose Schedule

Treatment	Description	Dose Schedule
A	4 doses of 4 mg IN naloxone doses	1 dose every 2.5 min; L at 0 min, R at 2.5 min, L at 5 min, R at 7.5 min
B	4 doses of 4 mg IN naloxone doses	2 doses every 2.5 min; L and R at 0* min, L and R at 2.5* min
C	2 doses of 4 mg IN naloxone doses	L at 0 min, R at 2.5 min

*L = Left Nostril, R = Right Nostril, * doses will be ~5 seconds apart (one after the other)*

Subjects will be randomized to one of six treatment sequences (i.e., ABC, ACB, BAC, BCA, CAB, CBA). Study drug will be administered by a clinical research nurse on the study clinic floor at the subject's bedside. The pharmacist and investigator will be available if needed during study drug administration. The subject should remain fully supine for approximately one hour post-dose. Subjects should be instructed not to breathe through the nose during administration of the nasal spray into the nose. Subjects will be instructed to leave their masks off for twenty minutes after drug administration.

IN naloxone doses will be administered as a 4 mg IN spray at the times specified above. No treatments will include placebo.

4.8.2 Dose Selection

IN naloxone is available as a single-dose spray containing 4 mg of naloxone hydrochloride in 0.1 mL. The 4 mg drug is distributed in packages of two nasal sprays (1 dose per spray), but additional doses can be administered if needed and available. IN naloxone labeling includes instructions to readminister additional doses using alternating nostrils, every 2 to 3 minutes (new nasal spray for each dose) if the patient does not respond or responds and then relapses into respiratory depression until medical assistance arrives. This may result in multiple doses of naloxone being required to achieve reversal, however, there is only PK data of IN naloxone following administration of 2 IN naloxone doses (in each nostril) at the same time. In this study, doses of IN naloxone (4 mg) were selected to compare administration of multiple doses of IN naloxone. The model-predicted naloxone exposures from 4 IN naloxone doses as will be administered in this study are Cmax 19 ng/mL and AUC0-inf 31 hr•ng/mL. These naloxone exposures are lower than exposures observed with intravenous administration of naloxone up to 10 mg IV (predicted: AUC0-inf 40-50 hr•ng/mL for 5 x 2 mg IV; predicted Cmax 25-30 ng/mL for 2 mg IV), which are covered by labeling and clinical use (Naloxone Hydrocholride Injection Generic Label, dated 03/18/2020; Tylleskar *et al.,* 2017; McDonald *et al.*, 2018).

4.8.3 Method of Assigning Subjects to Treatment Sequence

4.8.3.1 Randomization Process

The project biostatistician will create the specifications that will be used to generate the randomization schedule. The specifications will be based on the protocol requirements and appropriate statistical programming with consideration for study design, number of treatments, number of subjects planned for enrollment, stratification, and blocking.

Based on these specifications, the project biostatistician (or designee) will generate a dummy randomization schedule. The schedule is generated in R.

The project biostatistician (or designee) distributes the 'dummy' randomization schedule to specified personnel for review. Any change (e.g., change in block size, change in stratification levels) that requires an update to the specifications will reset this process. Minor changes (e.g., display formatting) will not require a change to the specifications.

After the approval of the 'dummy' randomization schedule, the project biostatistician (or designee) will generate the final randomization schedule. The output is sent only to designated recipients, who will maintain a secured digital and printed copy for their use.

Archival of the programs and output is accomplished by the creation of an encrypted, password-protected ZIP file containing the program and output file(s). The ZIP file is copied to a secure storage drive on the sponsor's site.

Randomization will occur after informed consent is obtained either after completion of check-in procedures on Day -1 or just before dosing on Day 1. Approximately 20 healthy subjects are planned for enrollment and up to 4 subjects may be qualified as replacements. Thus, a maximum of 24 subjects will be exposed to study drug and procedures during the study.

Enrolled subjects will be randomly assigned to one of the six different treatment sequences as mentioned in Section 4.8.2.

All randomization information will be secured and housed in a locked storage area.

4.8.4 Identity of Study Drug

NARCAN Nasal Spray (referred to throughout this document as IN naloxone) is indicated for the emergency treatment of known or suspected opioid overdose, as manifested by respiratory and/or central nervous system depression. NARCAN Nasal Spray is intended for immediate administration as emergency therapy in settings where opioids may be present and is available as a 2 mg or 4 mg single dose of naloxone hydrochloride in 0.1 mL with the indication to re-administer additional doses every 2 to 3 minutes (using alternating nostrils) if needed until emergency medical assistance arrives. NARCAN Nasal Spray is a pre-filled, single dose intranasal spray. Chemically, naloxone hydrochloride has a molecular weight of 363.84 and molecular formula $C_{19}H_{21}NO_4 \bullet HCl$. Naloxone hydrochloride, an opioid antagonist, occurs as a

white to slightly off-white powder, and is soluble in water, in dilute acids, and in strong alkali; slightly soluble in alcohol; practically insoluble in ether and in chloroform. (NARCAN Nasal Spray label, dated 08/06/2020)

4.8.5 Management of Clinical Supplies

4.8.5.1 Study Drug Packaging and Storage

The active study drug will be obtained from commercial sources. Storage instructions for the active study drug are as follows (NARCAN Nasal Spray label, dated 08/06/2020):

- Store NARCAN Nasal Spray in the blister and cartons provided. Store below 77°F (25°C). Excursions permitted up to 104°F (40°C). Do not freeze or expose to excessive heat above 104°F (40°C). Protect from light.
- NARCAN Nasal Spray freezes at temperatures below 5°F (-15°C). If this happens, the device will not spray. However, NARCAN Nasal Spray may be thawed by allowing it to sit at room temperature for 15 minutes, and it may still be used if it has been thawed after being previously frozen.

4.8.5.2 Study Drug Accountability

Good clinical documentation practices will be employed to record the receipt, storage conditions, accountability, and use or return of the study drug. The study drug will be stored in a secure location with access to the study personnel who will be managing the storage, dispensing, and accountability of the study drug.

Upon completion or termination of the study, final accountability review by the study monitor, and written authorization from the sponsor, all unused and/or partially used study drug should be returned or destroyed at the study clinic. It is the investigator's responsibility to ensure that the sponsor has provided written authorization for study drug disposal, the disposal process follows the study clinic's standard operating procedures, and appropriate records of the disposal are documented and maintained. No unused study drug may be disposed until fully accounted for by the study monitor (or designee). Documentation of unused study drug should include subject number, medication identity (medication #, period #), date, and quantity of study drug used.

4.8.6 Blinding

The study will be unblinded as the product is administered IN and different arms will have a different number of administrations at different times.

4.8.7 Treatment Compliance

At Screening, as part of the inclusion criteria, it will be confirmed that subjects are able to comply with the protocol-defined procedures including administration of an intranasal study

drug. All doses of the study drug will be administered in the study clinic by clinic personnel and recorded in the eCRF. If a subject sneezes after dosing, the event will be documented. The decision to replace any subject who sneezes after dosing will be made as described in Section 4.5.1.

4.8.8 Prior and Concomitant Medications

Subjects are prohibited from using any prescription or nonprescription drugs (including aspirin or non-steroidal anti-inflammatory drugs [NSAIDs] and excluding oral contraceptives and acetaminophen) within 14 days or 5 half-lives (whichever is longer), or complementary and alternative medicines within 28 days before the first dose of study drug. Subjects will be asked if they have used any of these substances and their responses will be recorded on the eCRF.

Subjects are also prohibited from currently participating in another clinical study of an investigational drug and may not have been treated with any investigational drug within 30 days or 5 half-lives (whichever is longer) of the compound.

Subjects must be instructed not to take any medications, including over-the-counter products, without first consulting with the investigator.

4.8.9 Subject Restrictions

Subjects are not allowed to use nicotine-containing products (e.g., cigarettes, cigars, chewing tobacco, snuff) within 6 weeks before screening. In addition, subjects are not allowed to ingest alcohol, xanthine-containing products (e.g., tea, coffee, chocolate, cola), caffeine, grapefruit, or grapefruit juice for 48 h before dosing and throughout the study.

Subjects must be able to tolerate a controlled, quiet study conduct environment, including avoidance of music, television, movies, games, and activities that may cause excitement, emotional tension, or arousal during prespecified times (e.g., before and during ECG extraction windows) throughout the duration of the study.

Subject must be able to take the drug intranasally and should not have a deviated septum or any previous nasal surgeries that would impact administration of the intranasal drug. Additionally, subjects must refrain from using any other nasal spray product beginning 48 h before dosing until completion of the study.

Subjects must be willing to comply with study rules; attempting to void at specified times; remaining quiet, awake, undistracted, motionless, and supine during specified times; and avoiding vigorous exercise as directed throughout the duration of the study.

Standardized meals will be served at consistent times relative to dosing, and no food or fluids will be served containing caffeine. Subjects must only eat meals and snacks that are provided during periods of their stay in the study clinic, and must consume all of each meal that is served

at a reasonable pace (within 25 minutes). Outside of meal times, the subjects will only be allowed to intake water, which will be available ad libitum.

Due to current precautions being taken for COVID-19, the following restrictions will be in place:

- Subjects must always wear masks except when in their beds with a curtain drawn between subjects or for a limited time for a study procedure (e.g. study drug administration or eating) when instructed by staff.
- Subjects must practice social distancing, which will include having 2 subjects per room for overnight stays and having common areas closed. Subjects will eat meals socially distanced in the hallway. Subjects will spend most of their time in their rooms except for specified times for walking in the halls (with masks).
- Subjects must practice regular handwashing with soap and water, scrubbing hands for at least 20 seconds or with approved hand sanitizer as supplied by study staff.

Designated isolation rooms will be set up to segregate any participant(s) that develop any symptoms of concern while housed in the unit and COVID-19 testing will be done when deemed necessary by the Investigator. If new information becomes available, there could be other precautions that lead to additional restrictions that will be documented in the COVID-19 Business Continuity Plan and the study specific COVID-19 Procedure Plan.

4.9 Statistical Methods

4.9.1 Sample Size

Up to 24 healthy subjects will be enrolled (including 4 potential replacement subjects). The number of subjects was determined to have greater than 80% power for comparisons between treatment arms across prespecified consecutive timepoints (different timepoints for each comparison). These power calculations are based on 18 subjects with each comparison, allowing for potential dropouts. These analyses adjust for multiplicity using Pocock boundaries for assessing the same endpoint at multiple timepoints. Coefficient of variations and effects sizes for different treatment scenarios were determined using modeling and simulation from previous naloxone studies.

4.9.1 Analysis Populations

The PK population will include all subjects who receive study drug and have at least one estimable PK parameter after dosing.

The safety population will include all subjects who receive at least one dose of the study drug.

The analysis population will include all subjects in the PK population with sufficient data to calculate PK parameters. Only subjects with results from two or more periods will be included in comparisons.

The details of the PK analysis will be included in the Statistical Analysis Plan (SAP). In any situation where the SAP and protocol differ regarding planned analyses, the language in the SAP supercedes descriptive text in the protocol.

4.9.2 General Statistical Considerations

All data will be presented in data listings. Data from subjects excluded from an analysis population will be presented in the data listings, but not included in the calculation of summary statistics. Demographic and baseline characteristics will be summarized overall and by treatment for all subjects.

4.9.3 Subject Disposition

The number of subjects who enroll in the study and the number and percentage of subjects who complete each assessment will be presented. The frequency and percentage of subjects who withdraw or discontinue from the study and the reason for withdrawal or discontinuation will be summarized.

4.9.4 Demographics and Baseline Characteristics

Descriptive statistics will be used to summarize demographic and baseline subject characteristics. For continuous variables, the mean, median, standard deviation (SD), minimum, and maximum values will be reported. For categorical (nominal) variables, the number and percentage of subjects (or observations) will be reported.

4.9.5 Pharmacokinetic Analysis

Pharmacokinetics: Concentrations will be determined at specified times. The primary endpoint is the first timepoint when there is a higher naloxone plasma concentration in the 4 naloxone dose arms compared to the 2 naloxone dose arm. The comparisons will consider multiple, pre-specified timepoints and adjust for multiplicity using Pocock boundaries. Separate comparisons will be performed looking at the 4 naloxone dose arm A (test) versus the 2 naloxone dose arm C (reference) and the 4 naloxone dose arm B (test) verus the 2 naloxone dose arm C (reference). Different timepoints will be selected for each comparison and timepoints will be evaluated from earliest to latest time.

Secondary endpoints will include determination of the first timepoint when there is a higher naloxone plasma concentration in the 4 naloxone dose arm B (2 doses every 2.5 minutes) as test compared to the 4 naloxone dose arm A (1 dose every 2.5 minutes) as reference. The same approach as outlined for the primary endpoint analysis above will be used for this comparison.

Additional PK measures (i.e., C_{max}, $AUC_{0\text{-}inf}$, $AUC_{0\text{-}t}$, pAUC, T_{max}, half-life, elimination rate) will be exploratory endpoints and determined for each subject using non-compartmental

methods. All parameters will be calculated using SAS or R software and will be reported with standard descriptive statistics including the mean and coefficient of variation. Calculation of PK parameters will be performed using actual sampling times. Additional details of the PK analysis will be included in the SAP.

Dose Proportionality: Dose proportionality based on C_{max}, AUC_{0-t}, and AUC_{0-inf} will be compared between each of the three naloxone treatments as secondary endpoints. The 4 naloxone doses arms (separate comparisons for 1 dose every 2.5 minutes and 2 doses every 2.5 minutes) will be compared with the 2 naloxone dose arm (1 dose every 2.5 minutes). An additional comparison will be performed between the 4 naloxone arm B (2 doses every 2.5 minutes) as test and 4 naloxone dose arm A (1 dose every 2.5 minutes) as reference.

4.9.6 PK/PD Modeling

PK data from this study will be used to update the naloxone PK model in a previously developed PK/PD model for opioid-induced respiratory depression. The updated model will be used to predict the mean time to rescue a patient from simulated opioid induced respiratory depression from fentanyl and carfentanil following a range of overdose scenarios (e.g. low, medium and high). Full details of the analysis will be described in a separate Modeling Analysis Plan (MAP).

4.9.7 Safety Analyses

4.9.7.1 Adverse Events

All AEs will be coded using the latest version of the Medical Dictionary for Regulatory Activities. The incidence of TEAEs, organized by system organ class and frequency, will be summarized by seriousness, severity, relationship to treatment, and by treatment at onset of the TEAE. A detailed listing of serious AEs and TEAEs leading to withdrawal will also be provided.

4.9.7.2 Clinical Laboratory Tests

Clinical laboratory results will be reviewed by the investigator or designee together with data in the eCRF. Any values outside the reference range will be evaluated for clinical significance. If a value is determined to be clinically significant, the subject will be instructed to follow-up with his or her personal physician. The investigator or designee may repeat the clinical laboratory tests if deemed appropriate. The investigator will maintain a copy of the laboratory accreditation and the reference ranges for the laboratory used.

Clinical laboratory results (hematology, serum chemistry, and urinalysis) will be summarized using descriptive statistics (number of subjects, mean, SD, minimum, median, and maximum). Clinical laboratory results will be classified as normal or abnormal, according to the reference ranges of the individual parameter. The number and percentage of subjects with abnormal

laboratory results will be provided. No statistical testing will be performed on clinical laboratory data.

4.9.7.3 Vital Sign Measurements

Vital sign measurements and changes from baseline will be summarized using descriptive statistics (number of subjects, mean, SD, minimum, median, and maximum) by treatment and time point.

4.9.7.4 Safety 12-lead Electrocardiograms

12-lead ECGs will be obtained with the subjects in the supine position for a minimum of 5 minutes before recording. ECGs will be overread by a physician. If an abnormality is observed, the subject will be instructed to follow-up with his or her personal physician.

4.9.7.5 Physical Examinations

Physical examination findings will be presented in a data listing, and abnormal physical examination findings will be recorded as AEs.

4.9.7.6 Nasal Irritation Score Analyses

Nasal Irritation Score is an examination of the nasal passage conducted by a trained observer to evaluate evidence of irritation to the nasal mucosa. This measures will provide an assessment of how much nasal irritation the subject is caused by each treatment during the study. These results will be summarized using descriptive statistics for all treatment groups.

4.9.7.7 Other Safety Data

All concomitant medication usage and medications that changed in daily dose, frequency, or both since the subject provided informed consent will be summarized for each subject.

4.9.8 Missing Data

Missing data will not be imputed. Data that are excluded from the descriptive or inferential analyses will be included in the subject data listings. This will include data from subjects not in the particular analysis population, measurements from unscheduled visits, or extra measurements that may arise from 2 or more analyses of the biofluid sample at the same time point. Details on the handling of missing data will be further described in the SAP.

4.10 Data Quality Assurance

Completed eCRFs are required for each subject randomly assigned to treatment. Electronic data entry will be accomplished through the ClinSpark® remote electronic data capture system, which allows for on-site data entry and data management. This system provides immediate, direct data

transfer to the database, as well as immediate detection of discrepancies, enabling site coordinators to resolve and manage discrepancies in a timely manner. Each person involved with the study will have an individual identification code and password that allows for record traceability. Thus, the system, and subsequently any investigative reviews, can identify coordinators, investigators, and individuals who have entered or modified records.

Furthermore, the investigator retains full responsibility for the accuracy and authenticity of all data entered into the electronic data capture system. The completed dataset and their associated files are the sole property of the sponsor and should not be made available in any form to third parties, except for appropriate governmental health or regulatory authorities, without written permission of the sponsor.

4.11 Data Sharing

De-identified subject-level data may be released to other researchers (including through a data warehouse or as a part of a publication) to enable secondary research. Additional secondary research may also be performed by the sponsor.

5. ETHICAL CONSIDERATIONS

5.1 Ethical Conduct of the Study

This study will be performed in accordance with the recommendations guiding physicians in biomedical research involving human subjects adopted by the 18th World Medical Association General Assembly, Helsinki, Finland, 1964 and later revisions, as well as, United States Title 45 Code of Federal Regulations (CFR) Part 46 GCP, and International Council for Harmonisation (ICH) guidelines describing technical requirements for registration of pharmaceuticals for human use.

5.2 Institutional Review Board (IRB)

The FDA Project Lead or investigator will provide the designated IRB with all required documents, including the study protocol and informed consent form. The study will not be initiated until appropriate IRB approval is obtained from the designated IRB. The investigator will provide the FDA Project Lead with copies of the approval documents for the protocol, informed consent form, and all recruiting materials. The designated IRB will also receive copies of any original or amended information sheets or pamphlets given to the study subject in support of the informed consent process and any advertisements or other recruitment material. Such materials will not be employed in the study before approval by the designated IRB.

Subjects will be informed that they have the right to contact the IRB or Office for Human Research Protections if they have any questions, concerns, complaints, or believe they have been

harmed by the participation in this research study as a result of investigator negligence. Subjects will be given the address and phone number of the IRB.

6. ADMINISTRATIVE PROCEDURES

6.1 Responsibilities of the Investigator

The following administrative items are meant to guide the investigator in the conduct of the study but may be subject to change based on industry and government standard operating procedures, working practice documents, or guidelines. Changes may be reported to the IRB but will not result in protocol amendments.

6.1.1 Form FDA 1572

The investigator will complete and sign the Form FDA 1572.

6.1.2 Adherence to Protocol

The investigator agrees to conduct the study as outlined in this protocol in accordance with the ICH E6(R2) and all applicable guidelines and regulations.

6.1.3 Reporting Requirements

By participating in this study, the investigator agrees to submit reports of SAEs according to the time line and method outlined in the protocol (Section 4.7.4.1.2). In addition, the investigator agrees to submit reports to the IRB as appropriate. The investigator also agrees to provide the sponsor with an adequate report shortly after completion of the investigator's participation in the study.

6.1.4 Source Documentation

By participating in this study, the investigator agrees to maintain adequate case histories for the subjects treated as part of the research under this protocol. The investigator agrees to maintain accurate eCRFs and source documentation as part of the case histories.

6.1.5 Retention of Records

The investigator agrees to keep the records stipulated in this protocol and those documents that include (but are not limited to) the study-specific documents, identification log of all participating subjects, medical records, source worksheets, all original signed and dated informed consent forms, subject authorization forms regarding the use of personal health information (if separate from the informed consent form), copies of all eCRFs, query responses, and detailed records of drug disposition, to enable evaluations or audits from regulatory authorities, the sponsor, or its designees.

Furthermore, ICH 4.9.5 requires the investigator to retain essential documents specified in Section 8 of ICH E6 (R2) until at least 2 years after the last approval of a marketing application for a specified drug indication being investigated or, if an application is not approved, until at least 2 years after the investigation is discontinued and regulatory authorities are notified. In addition, ICH 4.9.5 states that the study records should be retained until an amount of time specified by applicable regulatory requirements or for a time specified in the clinical study site agreement between the investigator and sponsor.

Refer to the clinical study site agreement for the sponsor's requirements on record retention. The investigator should contact and receive written approval from the sponsor before disposing of any such documents.

6.1.6 Financial Disclosure and Obligations

The investigator is required to provide financial disclosure information to allow the sponsor to submit the complete and accurate certification or disclosure statements required under 45 CFR 46. In addition, the investigator must provide to the sponsor a commitment to update this information promptly if any relevant changes occur during the course of the investigation and for 1 year after the completion of the study.

Neither the sponsor nor the study clinic is financially responsible for further testing or treatment of any medical condition that may be detected during the screening process.

6.2 Confidentiality and Disclosure of Data

All subjects will sign a HIPAA-compliant authorization form containing the mandated core elements and requirements before participation in this clinical study. The sponsor and designees affirm and uphold the principle of the subject's right to protection against invasion of privacy. Throughout this study, a subject's source data will only be linked to the sponsor's electronic data capture system database or documentation via a unique identification number. As permitted by all applicable laws and regulations, limited subject attributes such as gender, age or date of birth, and subject initials may be used to verify the subject and accuracy of the subject's unique identification number.

To comply with ICH Guidelines for GCP and to verify compliance with this protocol, the sponsor requires that the investigator allow review of the subject's original medical records (source data or documents) by the study monitor, representatives from any regulatory authority (e.g., FDA), the sponsor's designated auditors, and the appropriate IRB. These medical records will include, but will not be limited to, clinical laboratory test result reports, ECG reports, admission and discharge summaries for hospital admissions occurring during a subject's study participation, and autopsy reports. Access to a subject's original medical records requires the specific authorization of the subject as part of the informed consent process.

Copies of any subject source documents that are provided to the sponsor must have certain personally identifiable information removed (i.e., subject name, address, and other identifier fields not collected in the subject's eCRF).

Data will be maintained and backed up in the electronic data capture system. All access to the data is protected by username and password, and each staff member and all sponsor staff will have separate access that requires a separate username and password. Access is only given to site staff and requested sponsor staff who have completed the appropriate training.

6.3 Subject Consent

Written informed consent in compliance with 45 CFR 46 will be obtained from each subject before entering the study or performing any unusual or nonroutine procedure that involves risk to the subject. An informed consent template may be provided by the sponsor to the study clinic. If any institution-specific modifications to study-related procedures are proposed or made by the study clinic, the consent should be reviewed by the sponsor or its designee or both before IRB submission. Once reviewed, the consent will be submitted by the investigator to the IRB for review and approval before the start of the study. If the informed consent form is revised during the course of the study, all active participating subjects must sign the revised form.

Before enrollment, each prospective subject will be given a full explanation of the study and be allowed to read the approved informed consent form. The informed consent process will be performed by a clinical research nurse in a private room. The subject will be given unlimited time to ask questions regarding study participation, and each subject will be questioned to ensure their understanding. Once the investigator is assured that the subject understands the implications of participating in the study, the subject will be asked to give consent to participate in the study by signing the informed consent form.

The investigator will provide a copy of the signed informed consent form to the subject. The original form will be maintained in the subject's medical records at the site.

6.4 Data Collection

Full details of procedures for data collection and handling will be documented in the data management plan, which is initiated with the final protocol receipt. The data management plan is a changing document that evolves over the course of the study and is finalized by database lock.

6.5 Publications

No information related to or generated by this study will be released to the public until it has been reviewed by the sponsor. The sponsor shall own intellectual rights for the data and analysis resulting from this study. Authorship on publications will be determined by standard journal requirements.

7. STUDY MANAGEMENT

7.1 Medical Monitor

As outlined in ICH E6(R2) Section 5.3, the sponsor's designated medical monitor will be readily available to advise on trial-related medical questions or problems.

7.2 Study Monitoring

The sponsor or its designee will monitor the study to ensure that it is being conducted according to the protocol, GCP standards, and applicable region-specific requirements, and to ensure that study initiation, conduct, and closure are adequate. The investigators and the study clinic staff will be expected to cooperate fully with the study monitors and personnel or agents of the sponsor and be available during monitoring visits to answer questions sufficiently and to provide any missing information. The investigators and their institutions will permit direct access to source data/documents for study-related monitoring activities, audits, IRB reviews, and regulatory inspections.

During any on-site visits, the study monitor will:

- Check and assess the progress of the study
- Review all informed consent forms
- Review study data collected
- Conduct source document verification
- Identify any issues and address their resolution
- Verify that the facility remains acceptable
- Conduct study drug accountability

These monitoring activities will be done in order to verify that the:

- Data are authentic, accurate, and complete.
- The safety and rights of the subjects are being protected.
- The study is being conducted in accordance with the currently approved protocol (including any amendments), GCP, and all applicable regulatory requirements.

In addition, the sponsor, designated auditors, and government inspectors must be allowed access to eCRFs, source documents, and other study files that may be required to evaluate the conduct of the study.

7.3 Management of Protocol Amendments and Deviations

7.3.1 Modification of the Protocol

Any changes in this research activity, except those necessary to remove an apparent immediate hazard to the subject, must be submitted to the sponsor or designee and reviewed and approved by the local IRB before implementation. Amendments to the protocol must be submitted in writing to the investigator's IRB for approval before subjects are enrolled into an amended protocol.

7.3.2 Protocol Violations and Deviations

Any significant protocol deviations that the investigator or study clinic staff believes are of major importance (e.g., incorrect randomizations, subject enrolled but not eligible) should be reported to the sponsor and the investigator's IRB as soon as possible. Significant protocol deviations may include the following:

- Deviations from the inclusion/exclusion criteria that may affect subject safety
- Deviations (omission or delay) of safety monitoring procedures
- Deviations in the administration of the study drug
- Deviations in obtaining informed consent

All subjects who are enrolled and receive the study drug, regardless of whether they have a major protocol violation, must continue to be followed for safety for all follow-up study visits.

8. REFERENCE LIST

1. NARCAN Nasal Spray Label, dated 08/06/2020. Obtained from https://www.accessdata.fda.gov/drugsatfda_docs/label/2020/208411Orig1s004lbl.pdf.
2. Naloxone Hydrochloride Injection Generic Label, dated 03/18/2020. Obtained from https://dailymed.nlm.nih.gov/dailymed/drugInfo.cfm?setid=b1303f15-c48b-a44d-b28a-72f370094e02
3. Tylleskar I, Skulberg AK, Nilsen T, Skarra S, Jansook P, Dale O. Pharmacokinetics of a new, nasal formulation of naloxone. *Eur J Clin Pharmacol*. 2017;73(5):555-562. doi: 10.1007/s00228-016-2191-1
4. McDonald R, Lorch U, Woodward J, et al. Pharmacokinetics of concentrated naloxone nasal spray for opioid overdose reversal: Phase I healthy volunteer study. *Addiction*. 2018;113(3):484-493. doi:10.1111/add.14033

9. APPENDICES

9.1 Appendix A – Schedule of Events

Table 9-1: Schedule of Events

Pharmacokinetics of Multiple Repeated Doses of Intranasal Naloxone										
	Screening	Check-In and Baseline	Treatment Period							Check-out
Study Day	-28 to -2	-1	1	2	3	4	5	6	7	8
Hour										
Visit	1	2	2	2	2	2	2	2	2	2
Recruitment/Screening - healthy	X									
Screening Failures	X									
In House Residency		X	X	X	X	X	X	X	X	
Meals		X	X	X	X	X	X	X	X	
Informed Consent	X									
Medical History	X	X								
Comprehensive Physical Exam	X	X								X
Vital signs[1]	X	X	X	X	X	X	X	X	X	X
Height	X									
Weight	X	X								X
ECG - single[2]	1	1	3			3			3	1
Chemistry/hematology[3]	X	X								X
Urinalysis	X	X								X
Urine drug screen + alcohol screen	X	X								X
Pregnancy test[3] (females only)	X	X								X
FSH (females only)	X									
HIV test	X									
Hepatitis test	X									
SARS CoV-2 Molecular Test		X								
Study Agent preparation & administration - IN			X			X			X	
Plasma PK draw (See protocol for time points)			16			16			16	
Nasal Irritation Assessment (See protocol for time points)			6			6			6	
Con Med/AE Assessment	X	X	X	X	X	X	X	X	X	X
eSource Management		X								
			Dosing Day	Wash-out Day	Wash-out Day	Dosing Day	Wash-out Day	Wash-out Day	Dosing Day	

1 Vital signs will be taken at 0 (pre-dose), 0.5h, 1h and 6h timepoints on dosing days
2 Clinically notable results are repeated; ECG will be performed at 0 (pre-dose), 1h and 6h timepoints on dosing days
3 Serum test

9.2 Appendix B – List of Abbreviations

LIST OF ABBREVIATIONS

Abbreviation	Definition
AE	adverse event
$AUC_{0\text{-}inf}$	area under the concentration-time curve from 0 to infinity
$AUC_{0\text{-}t}$	area under the concentration-time curve from 0 to last quantifiable concentration
CFR	Code of Federal Regulations
C_{max}	maximum observed concentration
COVID-19	coronavirus disease of 2019
CDER	Center for Drug Evaluation and Research
DARS	Division of Applied Regulatory Science
ECG	Electrocardiogram
eCRF	electronic case report form
FDA	Food and Drug Administration
GCP	Good Clinical Practice
HBsAg	hepatitis B surface antigen
HepC	hepatitis C
HIPAA	Health Insurance Portability and Accountability Act
HIV	human immunodeficiency virus
ICH	International Council for Harmonisation
IRB	Institutional Review Board
IN	Intranasal
Kg	kilogram
MAP	modeling analysis plan
MedDRA	Medical Dictionary for Regulatory Activities
Mg	milligram
NSAID	nonsteroidal anti-inflammatory drug
pAUC	partial area under the curve
PD	Pharmacodynamic
PK	Pharmacokinetic
SAE	serious adverse event
SAP	statistical analysis plan
SARS-CoV-2	severe acute respiratory syndrome coronavirus 2
SD	standard deviation
$t_{1/2}$	terminal half-life
TEAE	treatment-emergent adverse event
t_{max}	time of C_{max}

9.3 Appendix C – Protocol Revision History

**PROTOCOL REVISION HISTORY**			
**Protocol Number**	**Version**	**Effective Date**	**Summary of Changes**
SCR-011	1.0	15 Feb 2021	Developed initial protocol

Statistical Analysis Plan

SCR-011: Clinical Study to Investigate the Pharmacokinetics of Multiple Repeated Doses of Intranasal Naloxone

Sponsor: U.S. Food and Drug Administration
White Oak Building #64, Room 2072
10903 New Hampshire Avenue
Silver Spring, MD 20993

Sponsor Study Lead: David Strauss, MD, PhD
Director, Division of Applied Regulatory Science
U.S. Food and Drug Administration
Telephone: 301-796-6323
Email: david.strauss@fda.hhs.gov

Sponsor Medical Monitor: Keith Burkhart, MD
U.S. Food and Drug Administration
Telephone: 301-796-2226
Email: keith.burkhart@fda.hhs.gov

Project Manager: Jeffry Florian, PhD
U.S. Food and Drug Administration
Telephone: 301-796-4847
Email: jeffry.florian@fda.hhs.gov

Study Monitor: Jill Brown
FDA IRB Project Manager
U.S. Food and Drug Administration

Version of SAP: 1.0

Date of SAP: 10 Mar 2021

CONFIDENTIAL

The concepts and information contained in this document or generated during the study are considered proprietary and may not be disclosed in whole or in part without the expressed written consent of the U.S. Food and Drug Administration.

Sponsor Signatures Page

Prepared by

Victoria Gershuny -S (Affiliate)

Digitally signed by Victoria Gershuny -S (Affiliate)
DN: c=US, o=U.S. Government, ou=HHS, ou=FDA, ou=People, 0.9.2342.19200300.100.1.1=2002874999, cn=Victoria Gershuny -S (Affiliate)
Date: 2021.03.10 18:34:25 -05'00'

Victoria Gershuny, PhD
Biologist, Division of Applied Regulatory Science
U.S. Food and Drug Administration

Date

Prepared by

Jeffry Florian -S

Digitally signed by Jeffry Florian -S
DN: c=US, o=U.S. Government, ou=HHS, ou=FDA, ou=People, cn=Jeffry Florian -S, 0.9.2342.19200300.100.1.1=2000498765
Date: 2021.03.10 18:37:42 -05'00'

Jeffry Florian, PhD
General Health Scientist, Division of Applied Regulatory Science
U.S. Food and Drug Administration

Date

Reviewed by

Robbert Zusterzeel -S

Digitally signed by Robbert Zusterzeel -S
DN: c=US, o=U.S. Government, ou=HHS, ou=FDA, ou=People, 0.9.2342.19200300.100.1.1=2001114905, cn=Robbert Zusterzeel -S
Date: 2021.03.10 18:46:29 -05'00'

Robbert Zusterzeel, MD, PhD
Contractor, Division of Applied Regulatory Science
U.S. Food and Drug Administration

Date

Approved by

David Strauss -S

Digitally signed by David Strauss -S
DN: c=US, o=U.S. Government, ou=HHS, ou=FDA, ou=People, cn=David Strauss -S, 0.9.2342.19200300.100.1.1=2000507494
Date: 2021.03.10 18:49:05 -05'00'

David Strauss, MD, PhD
Director, Division of Applied Regulatory Science
U.S. Food and Drug Administration

Date

Contents

Sponsor Signatures Page 2
Contents 3
Abbreviations and definitions 5
Change Log 6
1. Introduction 7
2. Study Objectives 7
3. Study Overview 7
3.1 Study Design 7
4. Study Endpoints 8
4.1 Primary Endpoint 8
4.2 Secondary Endpoints 8
4.3 Exploratory Endpoint 9
4.4 Sample Size 9
5. Analysis Populations 10
6. Data Screening and Acceptance 11
6.1 Handling of Missing and Incomplete Data 11
7. General Statistical Considerations 11
7.1 Subject Disposition 11
7.2 Demographic and Baseline Characteristics 11
7.3 Pharmacokinetic Analyses 12
7.3.1 Naloxone Concentration Comparisons 12
7.3.2 Dose Proportionality 13
7.3.3 Plasma Pharmacokinetics 13
7.4 Additional Secondary Analyses 14
7.4.1 PK/PD Modeling of Time to Reversal of Opioid Induced Respiratory Depression ... 14
7.5 Safety Analyses 15
7.5.1 Adverse Events 15
7.5.2 Clinical Laboratory Tests 15
7.5.3 Vital Sign Measurements 15

7.5.4 Safety 12-lead Electrocardiograms 15
7.5.5 Physical Examinations 15
7.5.6 Nasal Irritation Score Analyses 15
7.5.7 Other Safety Data 16
8. Data Quality Assurance 16
9. Appendices 17
9.1 Randomization schedule 17
10. References 18

Abbreviations and definitions

AE	Adverse event
AUC0-inf	Area under the plasma concentration time curve from time 0 extrapolated to infinity
$AUC_{0\text{-}t}$	Area under the concentration-time curve from 0 to last quantifiable concentration
C_{last}	Last quantifiable concentration
Cmax	Maximum observed plasma concentration
CV	Coefficient of variation
ECG	Electrocardiogram
eCFRs	Electronic case report forms
FDA	Food and Drug Administration
IN	Intranasal
inf	Infinity
K_{el}	Elimination rate constant
pAUC	partial area under the curve
PD	Pharmacodynamic
PK	Pharmacokinetic
SAP	Statistical analysis plan
SD	Standard deviation
$t_{1/2}$	Terminal half-life
TEAE	Treatment-emergent adverse event
Tmax	Time of maximum concentration (C_{max})

Change Log

Version / Date	Section	Summary of Changes
1.0 /<br>10 Mar 2021	General	Developed initial statistical analysis plan

1. Introduction

This document outlines the proposed statistical methods for data analysis of data collected from Protocol 'SCR-011: Clinical study to investigate the pharmacokinetics of multiple repeated doses of intranasal naloxone'.

The following statistical analysis plan (SAP) provides the framework for the summarization of the data from this study. The SAP may change due to unforeseen circumstances. Any changes made to the SAP will be documented in a change log within the SAP along with a rationale for the changes.

2. Study Objectives

The study objectives are:

1. To determine and compare the pharmacokinetics of intranasal (IN) naloxone between the 4-dose naloxone arms and the 2-dose naloxone arm.
2. To use the pharmacokinetic data from each of the 3 naloxone dosing schedules/doses to predict the time to reverse opioid-induced respiratory depression following different overdose scenarios based on pharmacokinetic/pharmacodynamic (PK/PD) models

3. Study Overview

3.1 Study Design

This study will be a randomized, unblinded, three-way crossover study to determine naloxone plasma concentration after administration of multiple doses of intranasal (IN) naloxone.

Table 3-1: Study Schedule

Day -1	Day 1	Days 2-3	Day 4	Days 5-6	Day 7	Day 8
Check-in	Treatment Period 1	Washout	Treatment Period 2	Washout	Treatment Period 3	Check-out

The following 3 treatments will be evaluated in a randomized order over the 3 treatment periods.

Table 3-2: Study Treatments

Treatment	Description
A	Four 4 mg IN naloxone doses (1 dose every 2.5 min; L at 0 min, R at 2.5 min, L at 5 min, R at 7.5 min)
B	Four 4 mg IN naloxone doses (2 doses every 2.5 min; L and R at 0* min, L and R at 2.5* min)
C	Two 4 mg IN naloxone doses (L at 0 min, R at 2.5 min)

*L = Left Nostril, R = Right Nostril, * doses will be ~5 seconds apart (one after the other)*

Healthy subjects will be randomized to one of six treatment sequences (i.e., ABC, ACB, BAC, BCA, CAB, CBA). FDA will prepare the randomization schedule. Subjects will report to the study site for screening from Days -28 to -2 and then will return to the site on Day -1 for baseline assessments and check-in. After check-in (Day -1), subjects will receive dosing for the 3 respective treatment periods on Days 1, 4 and 7. There will be two days of washout between each treatment period. Participants will be confined in the study clinic from Day -1 until the morning of Day 8. On dosing days, dosing will occur as per the treatment description and PK assessments will occur at the following time points:

- PK assessment: 0 (pre-dose), 2, 4.5, 7, 10, 12.5, 15, 20, 30, 45, 60, 120, 180, 240, 360, and 720 minutes

The subject should remain fully supine for approximately one-hour post-dose. Subjects should be instructed not to breathe through the nose during administration of the nasal spray into the nose. Subjects will be instructed to leave their masks off for twenty minutes after drug administration.

A summary of all assessments prior to and following study drug administration on Day 1, is described in the Protocol, Schedule of Events.

Subjects will be discharged from the study after completion of all study procedures. If a subject discontinues from the study prematurely, all procedures scheduled for the end of the study will be performed.

4. Study Endpoints

4.1 Primary Endpoint

First time point when there is a higher naloxone plasma concentration in the 4-dose naloxone arms compared to the 2-dose naloxone arm

4.2 Secondary Endpoints

1. First time point when there is a higher naloxone plasma concentration in the 4-dose naloxone arm B (2 doses every 2.5 minutes) compared to the 4-dose naloxone arm A (1 dose every 2.5 minutes)

2. Dose-proportionality of the 4-dose naloxone arms in reference to the 2 -dose naloxone arm based on Cmax, AUC_{0-inf}, and AUC_{0-t}

3. Predicted time to rescue a patient from simulated opioid-induced respiratory depression from fentanyl and carfentanil following medium and high overdose scenarios

4.3 Exploratory Endpoint

Naloxone C_{max}, AUC_{0-inf}, AUC_{0-t}, t_{max}, and partial AUC [pAUC] within the first 30 minutes of dosing

4.4 Sample Size

Twenty healthy participants are planned for enrollment with up to 4 replacements. Naloxone plasma concentration profiles and variabilities were calculated based on summary pharmacokinetic data from the IN naloxone product label. Predicted naloxone concentrations were determined for each of the proposed dosing treatments based on principles of superposition (i.e., for linear pharmacokinetics the total concentration of a drug in circulation is the sum of remaining concentration from each administered dose prior to the current measurement time) with a focus on comparing plasma concentrations at early timepoints. Power calculations were based on the ratio between concentrations for each of the two primary comparisons (Treatment A vs Treatment C and Treatment B vs Treatment C). Of the comparisons, the power for the 4-dose naloxone arm A (test) versus the 2-dose naloxone arm C (reference) was expected to be lower than the 4-dose naloxone arm B (test) versus the 2-dose naloxone arm C (reference). For the different proposed treatments, the ratio in concentrations and the coefficient of variation (CV) at each timepoint based on principles of superposition are shown in Table 4-1.

Table 4-1: Predicted concentration ratio and CV between treatments at different timepoints and power calculation at a 0.0221 significance level

Comparison	Time (min)	Concentration Ratio	CV	Power (n=18) at $p = 0.0221$
**Primary Endpoint (comparisons of 4-dose treatment arms vs. 2-dose treatment arm reference)**				
Treatment A vs Treatment C	**10**	1.36	68	49
	**12.5**	1.57	58	91
	**15**	1.78	48	100
Treatment B vs Treatment C	**4.5**	2	111	86
	**7**	2	90	94
	**10**	2	68	99
**Secondary Endpoint (comparison between the 4-dose treatment arms)**				
Treatment B vs Treatment A	**4.5**	2	111	86
	**7**	1.85	90	88
	**10**	1.47	68	68

As the intent of administering naloxone is rapid reversal of an overdose, the planned primary analysis is a comparison of naloxone concentrations between treatment arms across pre-specified consecutive time points. Each of the paired comparisons was assumed to include three pre-specified timepoints (timepoints correspond to those listed in Table 4-1). The time points will be evaluated sequentially with adjustments for multiplicity using Pocock boundaries (Pocock, 1977). For three pre-specified assessments, this would correspond to assessments at a 0.0221 significance level for comparisons at individual time points to maintain an overall 0.05 significance level.

Based on these considerations and the concentration ratios and CVs in Table 4-1, a sample size was determined so that at least one of the planned time points would have greater than 90% power at a 0.0221 significance level for a one sided, log-transformed paired comparison (for the primary endpoint). For concluding an increase in naloxone concentration for Treatment A compared to Treatment C at 15 minutes, at least 12 subjects would be necessary assuming a 1.78-fold increase in exposure and 48% CV for the treatments. For concluding an increase in naloxone concentration for Treatment B compared to Treatment C at 10 minutes, at least 12 subjects would have the desired power assuming a 2-fold increase in exposure and 68% CV for the treatments. The sample size was further increased to 20 subjects to account for discontinuations and situations where the CV values in Table 4-1 may be underestimated for repeat naloxone dosing.

5. Analysis Populations

The PK population will include all subjects who receive study drug and have at least one estimable PK parameter after dosing. The safety population will include all subjects who receive

at least 1 dose of the study drug. The analysis population will include all subjects in the PK population with sufficient data to calculate PK parameters. Only subjects with results from two or more periods will be included in comparisons. Treatment in all analysis populations will be assigned based upon the treatment which the subjects actually received.

6. Data Screening and Acceptance

6.1 Handling of Missing and Incomplete Data

The following approaches will be used for the handling of missing and incomplete data:

- Missing PK data (e.g., skipped plasma sample collected) will not be imputed.
- Missed dose, dose administered outside the window, or other issues with dose administration (e.g., product delivery fails) will result in PK data from that period for that subject being excluded from all treatment arm comparisons and summary statistics from that time point forward. Other situations, such as a subject sneezing in close proximity to dosing will be carefully documented by study staff and handling of these cases will be determined and documented before any concentration data is available.
- PK samples collected outside the protocol-defined sampling window (i.e., more than ± 0.5 minute from the scheduled time for the first 15 minutes of collections) will be excluded from analyses comparing naloxone concentrations at the specific time point that is outside the window.
- PK measurements below the lower limit of quantification will be set to zero for all analyses.

7. General Statistical Considerations

All data will be presented in data listings. Data from subjects excluded from an analysis population will be presented in the data listings, but not included in the calculation of summary statistics. Demographic and baseline characteristics will be summarized overall and by treatment for all subjects.

7.1 Subject Disposition

The number of subjects who enroll in the study and the number and percentage of subjects who complete each assessment will be presented. The frequency and percentage of subjects who withdraw or discontinue from the study and the reason for withdrawal or discontinuation will be summarized.

7.2 Demographic and Baseline Characteristics

Continuous demographic and baseline characteristic variables (age, height, weight, and body mass index) will be summarized overall and by treatment using descriptive statistics (number of subjects, mean, standard deviation [SD], median, minimum, and maximum). The number and percentage of subjects in each class of categorical demographic and baseline characteristic variables will also be summarized.

7.3 Pharmacokinetic Analyses

7.3.1 Naloxone Concentration Comparisons

The primary endpoint is the first timepoint when there is a higher naloxone plasma concentration in the 4-dose naloxone arms compared to the 2-dose naloxone arm. Separate comparisons will be performed with the 4-dose naloxone arm A (test) versus the 2-dose naloxone arm C (reference) and the 4-dose naloxone arm B (test) versus the 2-dose naloxone arm C (reference). No adjustment for multiplicity is planned for these separate comparisons.

Secondary endpoints will include determination of the first time point when there is a higher naloxone plasma concentration in the 4-dose naloxone arm B (2 doses every 2.5 minutes) as test compared to the 4-dose naloxone arm A (1 dose every 2.5 minutes) as reference. The same approach outlined for the primary endpoint analysis above will be used for this secondary endpoint comparison. The secondary endpoint analysis will be conducted regardless of the primary endpoint results.

Table 7-1: Pre-specified Timepoints and *p*-value Threshold for Comparing Naloxone Concentrations Across Treatments (Maintain Overall *p*-value Per Comparison at 0.05) (Pocock, 1977)

Comparison	Time (min)	Interim Analysis	*p*-value Threshold
	**10**	1	0.0221
	**12.5**	2	0.0221
Treatment A vs Treatment C (primary)	**15**	3 (final)	0.0221
	**4.5**	1	0.0221
	**7**	2	0.0221
Treatment B vs Treatment C (primary)	**10**	3 (final)	0.0221
	**4.5**	1	0.0221
	**7**	2	0.0221
Treatment B vs Treatment A (secondary)	**10**	3 (final)	0.0221

For both the primary and secondary analyses, up to three different time points have been pre-specified for each comparison. Time points will be evaluated from earliest to latest time (Table 7-1). Testing will be conducted sequentially along planned interim analyses (i.e., start at the initial time and progress forward, stopping if success is achieved) at a 0.0221 significance level. Testing will proceed through each time point until either one passes (reported as the earliest time where a difference in concentration is observed) or all pre-specified time points fail (no difference in naloxone concentrations between treatments for the selected time points).

For comparing treatment A to treatment C, the analysis will use naloxone concentration data collected at 10, 12.5, and 15 min. For both the treatment B to treatment C comparison and the treatment B to treatment A comparison, the pre-specified time points are 4.5, 7, and 10 min. All

subjects contributing naloxone concentration measures from both treatments at the pre-specified time points will be included in the analysis. At each of these timepoints, subject-level naloxone concentration will be log-transformed. Naloxone concentration values that are below the lower limit of quantification (i.e., zero) will result in that time point from that subject being removed for comparisons involving that treatment arm.

A paired Student's t-test will be used for comparing naloxone concentrations between treatments. To demonstrate an increase in naloxone concentration with the test compared to reference, it is necessary that the lower bound of the one-sided 97.79% interval for the geometric mean ratio excludes 1. The results will be transformed back to the original scale by exponentiation to provide treatment geometric means.

Normality assumption will be verified using the Shapiro-Wilke test for normality and homogeneity of variances will be verified using Levene's test. If either assumption is not valid, a Mann-Whitney-U test will be used for the comparisons rather than a t-test.

7.3.2 Dose Proportionality

Dose proportionality based on C_{max}, AUC_{0-t}, and AUC_{0-inf} will be compared between each of the three naloxone treatments as secondary endpoints. The 4-dose naloxone arms (separate comparisons for 1 dose every 2.5 minutes and 2 doses every 2.5 minutes) will be compared with the 2-dose naloxone arm (1 dose every 2.5 minutes). An additional comparison will be performed between the 4-dose naloxone arm B (2 doses every 2.5 minutes) as test and 4-dose naloxone arm A (1 dose every 2.5 minutes) as reference.

Dose-adjusted values for C_{max}, AUC_{0-t}, and AUC_{0-inf} will be calculated based on noncompartmental PK parameter results, normalized to per mg of naloxone administered. Using linear mixed-effects modeling, comparisons of log-transformed dose-adjusted PK parameters will be performed. Treatment will be a fixed effect. Subject will be included as a random effect on the intercept. Treatment differences on the log-scale will be estimated for the PK parameters. Geometric mean ratio and 90% CIs will be obtained by exponentiation of the treatment effect and 90% CIs based on the log-transformed scale. Dose-proportionality will be concluded if the confidence interval includes 1.

7.3.3 Plasma Pharmacokinetics

The following PK parameters will be determined for naloxone for each subject across all treatments:

- **AUC from time 0 to the sampling time corresponding to the last quantifiable concentration (C_{last}) (AUC_{0-t})**

  AUC_{0-t} will be calculated according to the linear trapezoidal rule from time 0 minutes to the last quantifiable concentration.

- **AUC from time 0 extrapolated to infinity (AUC_{0-inf})**

AUC_{0-t} will be extrapolated to infinity from the AUC from time 0 to the sampling time corresponding to the last quantifiable concentration plus the last quantifiable concentration divided by K_{el}, as follows:

$AUC_{0-inf} = AUC_{0-t} + C_{last}/K_{el}$

- **Partial AUCs (pAUC) over time intervals of 0 to 7 ($pAUC_{0-7}$), 0-10 ($pAUC_{0-10}$), 0-12.5 ($pAUC_{0-12.5}$), 0-15 ($pAUC_{0-15}$), 0-20 ($pAUC_{0-20}$), and 0-30 ($pAUC_{0-30}$) minutes**

  pAUC will be calculated according to the linear trapezoidal rule from time 0 minutes to 7, 10, 12.5, 15, 20, and 30 minutes after time from first dose

- **Maximum concentration (observed peak drug concentration) (C_{max})**

  C_{max} will be read directly from the observed concentrations.

- **Time at which C_{max} occurs (T_{max})**

  T_{max} will be read directly from the observed concentrations as the blood sampling time corresponding to C_{max}. The earlier time will be reported in cases where the same C_{max} value is observed for a subject at multiple time points

- **Terminal half-life ($t_{1/2}$)**

  The terminal half-life will then be calculated using the elimination rate constant as:

  $t_{1/2}=\ln(2)/K_{el}$

- **K_{el}**

  The terminal elimination rate constant, which will be determined from the terminal slope of the log-concentration curve using linear regression

The PK parameters will be analyzed using noncompartmental methods based on actual sampling times. Plasma concentrations below the limits of quantification will be set to zero for the purpose of this analysis (see Section 6). AUC_{0-inf}, $t_{1/2}$, and K_{el} for naloxone by treatment and subject will only be included if the subject has 3 or more concentration values on the terminal portion of the pharmacokinetic curve and with an adjusted coefficient of determination (R^2) greater than 0.80.

These PK parameters will be summarized using descriptive statistics (number of subjects, mean, SD, CV, median, minimum, and maximum) for naloxone.

All parameters will be calculated using R software. Mean and individual concentration-time profiles will be presented in graphs.

7.4 Additional Secondary Analyses

7.4.1 PK/PD Modeling of Time to Reversal of Opioid Induced Respiratory Depression

PK data from this study will be used to update the naloxone PK model in a previously developed PK/PD model for opioid-induced respiratory depression. The updated model will be used to predict the mean time to rescue a patient from simulated opioid induced respiratory depression from fentanyl and carfentanil following a range of overdose scenarios (e.g. low, medium and high). Full details of the analysis will be described in a separate Modeling Analysis Plan (MAP).

7.5 Safety Analyses

7.5.1 Adverse Events

All adverse events (AEs) will be coded using the latest version of the Medical Dictionary for Regulatory Activities (MedDRA). The incidence of treatment-emergent adverse event (TEAEs), organized by system organ class and frequency, will be summarized by seriousness, severity, relationship to treatment, and by treatment at onset of the TEAE. A detailed listing of serious AEs and TEAEs leading to withdrawal will also be provided.

7.5.2 Clinical Laboratory Tests

Clinical laboratory results (hematology, serum chemistry, and urinalysis) will be summarized using descriptive statistics (number of subjects, mean, SD, minimum, median, and maximum). Clinical laboratory results will be classified as normal or abnormal, according to the reference ranges of the individual parameter. The number and percentage of subjects with abnormal laboratory results will be provided. No statistical testing will be performed on clinical laboratory data.

7.5.3 Vital Sign Measurements

Vital sign measurements and changes from baseline will be summarized using descriptive statistics (number of subjects, mean, SD, minimum, median, and maximum) by treatment and timepoint.

7.5.4 Safety 12-lead Electrocardiograms

Abnormal 12-lead ECG findings will be recorded as AEs (not planned for each treatment period) in a data listing.

7.5.5 Physical Examinations

Abnormal physical examination findings will be recorded as AEs (not planned for each treatment period).

7.5.6 Nasal Irritation Score Analyses

Nasal Irritation Score is an examination of the nasal passage conducted by a trained observer to evaluate evidence of irritation to the nasal mucosa. These results will be summarized using descriptive statistics for all treatment groups.

7.5.7 Other Safety Data

All concomitant medication usage and medications that changed in daily dose, frequency, or both since the subject provided informed consent will be summarized for each subject.

8. Data Quality Assurance

Completed eCRFs are required for each subject randomly assigned to treatment. Electronic data entry will be accomplished through the ClinSpark® remote electronic data capture system, which allows for on-site data entry and data management. This system provides immediate, direct data transfer to the database, as well as immediate detection of discrepancies, enabling site coordinators to resolve and manage discrepancies in a timely manner. Each person involved with the study will have an individual identification code and password that allows for record traceability. Thus, the system, and subsequently any investigative reviews, can identify coordinators, investigators, and individuals who have entered or modified records.

Furthermore, the investigator retains full responsibility for the accuracy and authenticity of all data entered into the electronic data capture system. The completed dataset and their associated files are the sole property of the sponsor and should not be made available in any form to third parties, except for appropriate governmental health or regulatory authorities, without written permission of the sponsor.

9. Appendices

9.1 Randomization schedule

This is a 3-period, unblinded, crossover study where approximately 20 subjects will be enrolled to receive either two or four administrations of 4 mg intranasal naloxone following different administration schedules. Healthy subjects will be randomized to one of six treatment sequences (i.e., ABC, ACB, BAC, BCA, CAB, CBA), where treatment codes are summarized in the table below. The first 18 subjects enrolled will be randomized in blocks of 6. The remaining two subjects will be randomly placed in 2 of the 6 treatment sequences. The study plans for 1:1 enrollment of males and females, but the randomization will not account for sex and treatment sequence interactions.

Table 9-1: Treatment Codes and Treatment Names

Treatment Code	Treatment Name
A	Four 4 mg IN naloxone doses (1 dose every 2.5 min; L at 0 min, R at 2.5 min, L at 5 min, R at 7.5 min)
B	Four 4 mg IN naloxone doses (2 doses every 2.5 min; L and R at 0* min, L and R at 2.5* min)
C	Two 4 mg IN naloxone doses (L at 0 min, R at 2.5 min)

10.References

NARCAN Nasal Spray Label, dated 08/06/2020. Obtained from https://www.accessdata.fda.gov/drugsatfda_docs/label/2020/208411Orig1s004lbl.pdf

Pocock, S. (1977). Group Sequential Methods in the Design and Analysis of Clinical Trials. *Biometrika, 64*(2), 191-199.

Model Analysis Plan

SCR-011: Clinical Study to Investigate the Pharmacokinetics of Multiple Repeated Doses of Intranasal Naloxone

Sponsor: U.S. Food and Drug Administration
White Oak Building #64, Room 2072
10903 New Hampshire Avenue
Silver Spring, MD 20993

Sponsor Study Lead: David Strauss, MD, PhD
Director, Division of Applied Regulatory Science
U.S. Food and Drug Administration
Telephone: 301-796-6323
Email: david.strauss@fda.hhs.gov

Sponsor Medical Monitor: Keith Burkhart, MD
U.S. Food and Drug Administration
Telephone: 301-796-2226
Email: keith.burkhart@fda.hhs.gov

Project Manager: Jeffry Florian, PhD
U.S. Food and Drug Administration
Telephone: 301-796-4847
Email: jeffry.florian@fda.hhs.gov

Study Monitor: Jill Brown
FDA IRB Project Manager
U.S. Food and Drug Administration

Version of MAP: 1.0

Date of MAP: 25th October 2021

CONFIDENTIAL

The concepts and information contained in this document or generated during the study are considered proprietary and may not be disclosed in whole or in part without the expressed written consent of the U.S. Food and Drug Administration.

Sponsor Signatures Page

Prepared by

Zhihua Li -S

Digitally signed by Zhihua Li -S
DN: c=US, o=U.S. Government, ou=HHS, ou=FDA, ou=People, cn=Zhihua Li -S, 0.9.2342.19200300.100.1.1=2000931442
Date: 2021.10.25 09:11:20 -04'00'

Zhihua Li, PhD
Biologist, Division of Applied Regulatory Science
U.S. Food and Drug Administration

Date

Reviewed by

Jeffry Florian -S

Digitally signed by Jeffry Florian -S
DN: c=US, o=U.S. Government, ou=HHS, ou=FDA, ou=People, cn=Jeffry Florian -S, 0.9.2342.19200300.100.1.1=2000498765
Date: 2021.10.24 20:55:54 -04'00'

Jeffry Florian, PhD
Associate Director, Division of Applied Regulatory Science
U.S. Food and Drug Administration

Date

Approved by

David Strauss -S

Digitally signed by David Strauss -S
DN: c=US, o=U.S. Government, ou=HHS, ou=FDA, ou=People, cn=David Strauss -S, 0.9.2342.19200300.100.1.1=2000507494
Date: 2021.10.25 10:49:33 -04'00'

David Strauss, MD, PhD
Director, Division of Applied Regulatory Science
U.S. Food and Drug Administration

Date

Contents

Sponsor Signatures Page 2
Contents 3
Abbreviations and definitions 4
Change Log 5
1. Introduction 6
2. Background 6
3. Modeling Plan 7
3.1 Model to be used 7
3.1.1 Receptor binding model 7
3.1.2 Pharmacokinetic model 7
3.1.3 Physiological model 8
3.1.4 Pharmacodynamic model 8
3.2 Model simulation of naloxone reversal of opioid overdose in a community setting 8
3.2.1 Estimation of dose levels of community opioid overdose cases 8
3.2.2 Estimation of the time to give the first dose of naloxone in a community setting 9
3.2.3 Estimation of rescue time and percent of patients rescued 10
4. References 12

Abbreviations and definitions

CO_2	Carbon dioxide
FDA	Food and Drug Administration
HHS	Department of Health and Human Services
IN	Intranasal
IV	Intravenous
inf	Infinity
K_{on}	Association constant
K_{off}	Dissociation constant
MAP	Model analysis plan
O_2	Oxygen
PD	Pharmacodynamic
PK	Pharmacokinetic
SAP	Statistical analysis plan

Change Log

Version / Date	Section	Summary of Changes
1.0 / Oct 25, 2021	General	Developed initial modeling analysis plan

1. Introduction

This document outlines the proposed model analysis methods to be applied to data from the study 'SCR-011: Clinical study to investigate the pharmacokinetics of multiple repeated doses of intranasal naloxone' and is a supplement to the statistical analysis plan (SAP).

The following model analysis plan (MAP) provides the framework for addressing study objective #2 as outlined in the SAP:

- To use the pharmacokinetic data from each of the 3 naloxone dosing schedules/doses to predict the time to reverse opioid-induced respiratory depression following different overdose scenarios based on pharmacokinetic/pharmacodynamic (PK/PD) models

Outputs from this MAP will fulfill Secondary Endpoint #3 as defined in the SAP:

- Predicted time to rescue a patient from simulated opioid-induced respiratory depression from fentanyl and carfentanil following medium and high overdose scenarios

The MAP may change due to unforeseen circumstances. Any changes made to the MAP will be documented in a change log within the MAP along with a rationale for the changes.

2. Background

The United States is going through a severe opioid epidemic which has been declared as a public health emergency by HHS in 2017. The number of deaths from opioid overdose has been steadily increasing over the years and was 49,860 in 2019 [1].

The opioid antagonist naloxone can reverse the respiratory depression effects of opioids and is being increasingly used in the prehospital setting, including by community members or bystanders without medical training, to rescue patients from opioid overdoses [2]. However, there is no universal acceptance of recommended dosing of naloxone in the community setting for suspected opioid overdose. In a joint meeting between the Anesthetic and Analgesic Drug Products Advisory Committee and the Drug Safety and Risk Management Advisory Committee, held by FDA in 2016, the issue of increasing the current pharmacokinetic (PK) standard for approval of naloxone products for community use was discussed. While a slight majority voted to increase the minimum dose, a consensus was not reached, and the reason cited was the absence of proper scientific evidence to inform accurate dosing [3].

The prediction of required naloxone dosing for rescue from opioid induced respiratory depression is difficult because of a multitude of factors including that general community dosing recommendations are made without knowing the specifics of an individual overdose, including details such as the opioid used and its potency, the route of opioid administration and the opioid dose. Moreover, even opioids agonists of similar opioid receptor binding potency, administered in the same quantity through the same route can have different depressing effects on respiration (i.e., due to different pharmacokinetic profiles and/or receptor binding kinetics). In addition, naloxone administration (both dose and administration route) and the binding kinetics of naloxone vs. the opioid agonist at the μ-receptor affect the reversal of opioid effects. Due to the plethora of synthetic

opioids available in the market, with potencies differing by multiple-log fold, it is unrealistic to conduct clinical studies in the indicated population to evaluate naloxone dosing strategies for all opioids. This is even more so for opioids that are not approved for human use.

3. Modeling Plan

3.1 Model to be used

An integrated model will be used to predict the naloxone-induced rescue from respiratory depression due to opioid overdose. The model will consist of different mechanistic sub-models:

1. A receptor binding model describing kinetic binding of opioids and opioid antagonists (naloxone) to the opioid μ-receptor

2. Pharmacokinetic models describing the plasma concentration of different opioids and the opioid antagonist, naloxone, for different doses, formulations and administration schedules

3. A physiological model describing gas (O_2 and CO_2) storage and exchange, ventilatory control, and blood flow control:

    a) Gas (O_2 and CO_2) storage and exchange incorporates three tissue compartments: the lungs, brain tissue and other body tissues combined together

    b) Ventilatory control incorporates the action of the central and peripheral chemoreceptor, and the effect of gases on respiratory neuron activity

    c) Blood flow control incorporates local mechanisms that regulate blood flow to organs with higher metabolic requirements, as well as systemic mechanisms to trigger cardiovascular collapse and subsequently cardiac arrest due to severe, prolonged hypoxia

4. A pharmacodynamic model describing the relationship between the fraction of opioid agonist-bound μ-receptor (due to the competitive binding between opioid agonists and antagonists) and human ventilatory response

3.1.1 Receptor binding model

For the receptor binding model, in vitro association, and dissociation experiments between ligands (opioids such as fentanyl and carfentanil, as well as the opioid antagonist naloxone) and human opioid μ-receptor were conducted and used to estimate drug-specific binding parameters K_{on}, K_{off}, and n (Hill coefficient). The estimated binding parameters and equations were validated using dose-dependent dislocation data of individual opioids from the μ-receptor when combined with naloxone.

3.1.2 Pharmacokinetic model

For the fentanyl PK model, a published PK model for fentanyl [4] was used. Because there is no reliable human pharmacokinetic data available for carfentanil, the fentanyl PK model was used as

a template, and limited human carfentanil plasma data [5] was used to adjust the carfentanil PK model.

For the intranasal naloxone PK model, we previously developed a PK model based on plasma concentration data following one 4 mg dose in one nostril and one 4 mg dose in each nostril administered at approximately the same time [6]. The new clinical data from this study will be used to update the PK model, to take into consideration how intranasal pharmacokinetics may be impacted with repeated dosing to the same nostril, including at different timings after the first dose.

3.1.3 Physiological model

The physiological model (combining gas O_2 and CO_2 storage and exchange, ventilatory control, and blood flow control) was implemented based on the work of Magosso, Ursino and colleagues [7, 8, 9] with some modifications. Their model has been shown to reproduce a wide range of clinical data covering different combinations of CO_2 and O_2 changes and their impact on human ventilation with and without opioid administration.

3.1.4 Pharmacodynamic model

A pharmacodynamic component was used to link the receptor binding model to the physiology model, allowing the prediction of human ventilation due to different levels of μ-receptor binding (by opioids). The pharmacodynamic component describes the relationship between receptor occupancy and human ventilation response, which was estimated based on fentanyl data [4, 9], and then validated by comparing model prediction to other clinical data involving different fentanyl derivatives.

3.2 Model simulation of naloxone reversal of opioid overdose in a community setting

3.2.1 Estimation of dose levels of community opioid overdose cases

Doses for the naloxone reversal of opioid overdose simulations were calculated based on postmortem distribution of opioid concentration data in fatal overdose cases. For fentanyl, data from approximately 500 cases were used [10]. For carfentanil, where large scale postmortem opioid distribution data were not readily available, the overdose calculations were based on the minimum simulated fentanyl and carfentanil doses that would result in cardiovascular collapse without an intervention. The doses proposed for the model simulations of naloxone reversal for both fentanyl and carfentanil are described in more detail below.

According to an analysis of approximately 500 unintentional fentanyl overdoses in New Hampshire [10], the postmortem plasma fentanyl concentration had a mean of 9.96 ng/mL, standard deviation of 9.27 ng/ml, and range of 0.75-113.00 ng/mL. Based on the mean and standard deviation of the postmortem fentanyl distribution, the 84.1th percentile concentration, which is one standard deviation above the mean, is estimated to be 19.2 ng/ml. In simulations, the mean will be considered a "medium" overdose scenario ($Dose_{fent,med}$) and the 84.1th percentile a "high" overdose scenario ($Dose_{fent,high}$). Based on the postmortem fentanyl plasma concentration, the initial IV dose corresponding to the postmortem concentrations was calculated using our PK model. This was done with the assumption that death occurs and that there is no significant postmortem redistribution of fentanyl [11]. These doses are shown below in Table 3-1.

For carfentanil, for which large scale postmortem plasma distribution data are not readily available, the IV doses were calculated based on the minimum simulated fentanyl ($Dose_{fent,CA}$) and carfentanil ($Dose_{car,CA}$) dose predicted to result in cardiovascular arrest without administering an opioid antagonist. The ratio of these two doses ($R_{CA} = Dose_{car,CA} / Dose_{fent,CA}$) was used to calculate the carfentanil dose for the overdose scenarios. The carfentanil "medium" overdose scenario was calculated as R_{CA}*($Dose_{fent,med}$) and the "high" overdose scenario was R_{CA}*($Dose_{fent,high}$). Carfentanil doses are listed in Table 1.

Table 3-1: Fentanyl and Carfentanil Doses in Naloxone Reversal Simulations

Scenario	Fentanyl Dose (mg)[a]	Carfentanil Dose (mg)[b]
Medium	1.625	0.012
High	2.965	0.02187

[a]Fentanyl doses were calculated based on distributions of post-mortem data [10]
[b]Carfentanil doses were calculated based on simulated doses predicted to result in cardiovascular collapse without an intervention and scaled based on the fentanyl post-mortem medium and high dose scenarios

Additional dosing scenarios with fentanyl and carfentanil or dosing scenarios with other fentanyl derivatives may be considered to better understand opioid reversal with the integrated model.

3.2.2 Estimation of the time to give the first dose of naloxone in a community setting

In the overdose simulations, naloxone will be administered according to guidance from naloxone training materials in a community setting. It is suggested that naloxone be administered when the respiratory rate becomes less than 8 breaths per minute [12]. This is approximately equal to a 50% reduction in respiratory rate, which has a range of 12-20 breaths per minute [13]. At the same time, animal studies [14] have suggested that the decrease in tidal volume under the influence of fentanyl is about half of the decrease in respiratory rate. As minute ventilation = respiratory rate * tidal volume, we calculated the minute ventilation to be ((100-50)/100)*((100-25)/100)*100 ≈ 40% of the baseline ventilation, when naloxone is administered according to the training material. Other researchers have also referred to this as an opioid-induced 'unsafe' level of ventilation [15].

To account for practical delays in administering naloxone by first responders, naloxone will be administered in our simulations 1 minute after minute ventilation drops below 40% of the baseline. At the calculated naloxone administration time, naloxone will be administered intranasally into the simulation following different dosing regimens studied in the new clinical trial (see Treatments A, B and C in Table 3-2 below).

Table 3-2: Study Treatments

Treatment	Description
A	Four 4 mg IN naloxone doses (1 dose every 2.5 min; L at 0 min, R at 2.5 min, L at 5 min, R at 7.5 min)
B	Four 4 mg IN naloxone doses (2 doses every 2.5 min; L and R at 0* min, L and R at 2.5* min)
C	Two 4 mg IN naloxone doses (L at 0 min, R at 2.5 min)

*L = Left Nostril, R = Right Nostril, * doses will be ~5 seconds apart (one after the other)*

Additional naloxone dosing schedules beyond those listed above may be evaluated with simulations to better understand opioid reversal with the integrated model.

3.2.3 Estimation of rescue time and percent of patients rescued

In reviewing the literature [16, 17, 18, 19, 20] and from pilot simulations conducted with our model, we observed that the following physiological variables are potentially important markers of different stages of recovery:

- Brain tissue oxygen tension ≥ 20 mm Hg
  - Approximate upper range of brain hypoxia threshold from the literature
- Arterial pO_2 > 30 mm Hg
  - Above the approximate upper range of arterial pO_2 at the time of cardiac arrest in animal models of asphyxia
- Arterial oxygen saturation ≥ 90%
  - Above this the hemoglobin saturation curve flattens such that further increases in pO_2 have less effect on oxygen saturation
- Arterial pCO_2 ≤ 45 mm Hg
  - Upper range of normal for arterial pCO_2

The time when each of the above physiological variables first crosses the threshold and then recovers back to the critical threshold will be reported. For the purpose of reporting the secondary endpoint as specified in the clinical trial statistical analysis plan, rescue times will be reported based on the time brain tissue oxygen tension is below the threshold (i.e., the time between two time points: time point 1 is when brain tissue oxygen tension first drops below 20 mm Hg, and time point 2 is when brain tissue oxygen tension first recovers back above 20 mm Hg). If cardiovascular collapse and subsequent cardiac arrest occurs, the rescue time will be reported as infinity (i.e., the patient died).

To account for the uncertainty in parameter estimation, the overdose simulations will be performed for a virtual population consisting of 2000 patients. The 2000 patients in the virtual population will be allowed to have different fractions of opioid-bound μ-receptor by allowing patients to have different PK and binding parameters for the opioid and naloxone. The PK parameters will be

randomly sampled based on the mean and standard deviation of the population opioid and naloxone PK parameters. This will be combined with distributions of opioid- and naloxone-specific receptor binding parameters estimated from the in vitro data.

For all times when physiological thresholds are crossed and for the calculated rescue times for each opioid and naloxone dosing scenario, the values for the single ‘typical’ or ‘average’ virtual patient will be reported along with the median and interquartile range (25th and 75th percentiles) for the virtual population. In addition, the percentage of patients in the virtual population predicted to experience cardiac arrest will be calculated for each opioid and naloxone dosing scenario.

For the purpose of reporting the secondary endpoint on predicted time to rescue a patient as specified in the clinical trial statistical analysis plan, the median and interquartile range for the virtual population will be reported.

4. References

[1] L. J. Tanz, K. Quinn, Kariisa, M. Kariisa, P. Patel and N. L. Davis, "Trends and Geographic Patterns in Drug and Synthetic Opioid Overdose Deaths — United States, 2013–2019," *MMWR Morb Mortal Wkly Rep,* p. 70:202–207, 2021.

[2] L. R. Rzasa and J. L. Galinkin, "Naloxone dosage for opioid reversal: current evidence and clinical implications," *Therapeutic Advances in Drug Safety.,* pp. 9(1):63-88, 2018.

[3] FDA, *Summary Minutes of the Joint Meeting of the Anesthetic and Analgesic Drug Products Advisory Committee and the Drug Safety and Risk Management Advisory Committee,* 2016.

[4] M. H. Algera, E. Olofsen, L. Moss, R. L. Dobbins, M. Niesters, M. van Velzen, G. J. Groeneveld, J. Heuberger, C. M. Laffont and A. Dahan, "Tolerance to Opioid-Induced Respiratory Depression in Chronic High-Dose Opioid Users: A Model-Based Comparison With Opioid-Naïve Individuals," *Clinical Pharmacology & Therapeutics,* vol. 109, pp. 637-645, 2021.

[5] C. P. Minkowski, D. Epstein, J. J. Frost and D. A. Gorelick, "Differential Response to IV Carfentanil in Chronic Cocaine Users and Healthy Controls," *Addict Biol.,* vol. 17, no. 1, pp. 149-155, 2012.

[6] FDA, "NARCAN Nasal Spray Label," 2015.

[7] M. Ursino, E. Magosso and . G. Avanzolini, "An integrated model of the human ventilatory control system: the response to hypercapnia," *Clinical Physiology,* pp. 21(4):447-64, JulY 2001.

[8] M. Ursino, E. Magosso and G. Avanzolini, "An integrated model of the human ventilatory control system: the response to hypoxia," *Clinical Physiology,* vol. 21, no. 4, pp. 465-477, 2001.

[9] E. Magosso, M. Ursino and J. H. van Oostrom, "Opioid-Induced Respiratory Depression: A Mathematical Model for Fentanyl," *IEEE Transactions on Biomedical Engineering,* vol. 51, no. 7, pp. 1115-1128, 2004.

[10] NDEWS, "Unintentional Fentanyl Overdoses in New Hampshire: An NDEWS HotSpot Analysis.," 2017.

[11] L. Brockbals, S. N. Staeheli, D. Gascho, L. C. Ebert, T. Kraemer and A. E. Steuer, "Time-Dependent Postmortem Redistribution of Opioids in Blood and Alternative Matrices," *Journal of Analytical Toxicology,* pp. 42(6): 365-374., 2018.

[12] NOSORH, "Manual for Nurse Practitioners: Using a Computer-Based Naloxone Training Module".

[13] K. E. Philip, E. Pack, V. Cambiano, H. Rollmann, S. Weil and J. O'Beirne, "The accuracy of respiratory rate assessment by doctors in a London teaching hospital: a cross-sectional study," *J Clin Monit Comput.,* pp. 29(4): 455-60., 2015.

[14] R. Hill, R. Santhakumar, W. Dewey, E. Kelly and G. Henderson, "Fentanyl depression of respiration: Comparison with heroin and morphine," *Br J Pharmacol.,* pp. 177(2): 254-266, 2020.

[15] C. J. Voscopoulos, C. M. MacNabb, J. Freeman, S. M. Galvagno Jr., D. Ladd and E. George, "Continuous noninvasive respiratory volume monitoring for the identification of patients at risk for opioid-induced respiratory depression and obstructive breathing patterns," *The Journal of Trauma and Acute Care Surgery,* pp. 77(3 Suppl 2):S208-15, 2014.

[16] E. Ortiz-Prado, J. F. Dunn, J. Vasconez, D. Castillo and G. Viscor, "Partial pressure of oxygen in the human body: a general review," *American journal of blood research,* pp. 9(1), 1–14, 2019.

[17] S. P. Gopinath, A. B. Valadka, M. Uzura and C. Robertson, "Comparison of jugular venous oxygen saturation and brain tissue PO2 as monitors of cerebral ischemia after head injury," *Critical Care Medicine,* vol. 27, no. 11, pp. 2337-2345, 1999.

[18] L. Longhi, F. Pagan, V. Valeriani, S. Magnoni, E. R. Zanier, V. Conte, V. Branca and N. Stocchetti, "Longhi L, Pagan F, Valeriani V, MagnoniMonitoring brain tissue oxygen tension in brain-injured patients reveals hypoxic episodes in normal-appearing and in peri-focal tissue," *Intensive Care Med,* vol. 33, no. 12, pp. 2136-42, 2007.

[19] J. Nortje and A. K. Gupta, "The role of tissue oxygen monitoring in patients with acute brain injury," *British Journal of Anaesthesia,* vol. 97, no. 1, pp. 95-106, 2006.

[20] R. Imberti, G. Bellinzona and M. Langer, "Cerebral tissue PO2 and SjvO2 changes during moderate hyperventilation in patients with severe traumatic brain injury," *J. Neurosurg.,* vol. 96, pp. 97-102, 2002.
