# Supplementary material for: Intranasal Naloxone Repeat Dosing Strategies and Fentanyl Overdose: A Simulation-Based Randomized Clinical Trial
Source: JAMA Netw Open. 2024 Jan 23;7(1):e2351839. doi: 10.1001/jamanetworkopen.2023.51839 (PMC10807299; doi:10.1001/jamanetworkopen.2023.51839)
Supplement: Supplement 2. — eMethods 1. Bioanalytical Method Conditions for Naloxone in Human Plasma eMethods 2. Modeling eFigure 1. Model-Predicted Outcomes of Fentanyl and Carfentanil Overdoses With and Without Naloxone eFigure 2. Model-Predicted Cardiac Arrest Outcomes With Carfentanil Overdoses and Intranasal or Intravenous Naloxone eFigure 3. Effect of Changing the Delay Between Ventilatory Depression and the First Naloxone Dose eTable 1. Exploratory Outcomes eTable 2. Study Participant Demographics eTable 3. Number of Participant Samples Included in Analyses eTable 4. Primary Pharmacokinetic Outcomes eTable 5. Secondary Pharmacokinetic Outcomes eTable 6. Exploratory Pharmacokinetic Measures by Naloxone Treatment Group eTable 7. Incidence and Number of Adverse Events by Treatment Group eTable 8. Model-Predicted Rescue Times Based on Different Dosing Scenarios and Respiratory Measures eAppendix. Data Dictionary for Naloxone Pharmacokinetic Data Set eReferences [file jamanetwopen-e2351839-s002.pdf]

Supplementary Online Content

Strauss DG, Li Z, Chaturbedi A, et al. Intranasal naloxone repeat dosing strategies and fentanyl overdose: a simulation-based randomized clinical trial. *JAMA Netw Open*. 2024;7(1):e2351839. doi:10.1001/jamanetworkopen.2023.51839

eMethods 1. Bioanalytical Method Conditions for Naloxone in Human Plasma

eMethods 2. Modeling

eFigure 1. Model-Predicted Outcomes of Fentanyl and Carfentanil Overdoses With and Without Naloxone

eFigure 2. Model-Predicted Cardiac Arrest Outcomes With Carfentanil Overdoses and Intranasal or Intravenous Naloxone

eFigure 3. Effect of Changing the Delay Between Ventilatory Depression and the First Naloxone Dose

eTable 1. Exploratory Outcomes

eTable 2. Study Participant Demographics

eTable 3. Number of Participant Samples Included in Analyses

eTable 4. Primary Pharmacokinetic Outcomes

eTable 5. Secondary Pharmacokinetic Outcomes

eTable 6. Exploratory Pharmacokinetic Measures by Naloxone Treatment Group

eTable 7. Incidence and Number of Adverse Events by Treatment Group

eTable 8. Model-Predicted Rescue Times Based on Different Dosing Scenarios and Respiratory Measures

eAppendix. Data Dictionary for Naloxone Pharmacokinetic Data Set

eReferences

This supplementary material has been provided by the authors to give readers additional information about their work

eMethods 1. Bioanalytical Method Conditions for Naloxone in Human Plasma

A liquid chromatography tandem mass spectrometric method was developed and validated for the determination of Naloxone in K_2EDTA human plasma to support the analysis of clinical study samples. Analytical method was developed with the aim of high sensitivity, selectivity, and reliable quantitation. While optimizing the lower limit of quantitation (LLOQ), various parameters such as signal to noise, accuracy, and precision were evaluated. The upper limit of quantitation (ULOQ) was selected based on the concentration range at which acceptable linearity was obtained (i.e., concentration vs linear detector response). Excellent peak shape was obtained using the optimized chromatographic conditions. Sample extraction procedure was optimized to ensure high and consistent analyte recovery area. Summary of validation results is described below.

© 2024 Strauss DG et al. *JAMA Network Open.*

Liquid chromatography tandem mass spectrometric method summary:
Instrument: AB Sciex 6500 mass spectrometer coupled to liquid chromatographic system
Columns: Zorbax SB- C_{18} (50 x 2.1 mm) 5µ at RT
Mobile Phase A:5 mM Ammonium Bicarbonate, pH 7.5
Mobile Phase B: Acetonitrile

Validation Summary Table:

**Description**	**Naloxone**
Short description of method	Liquid-liquid extraction after derivatization with propionic anhydride<br>Reverse-phase HPLC with tandem mass spectrometry
Analyte/Internal Standard	Naloxone/ Naloxone-d_5
Linearity Range	20.0 pg/mL to 20000.0 pg/mL
Selectivity	No significant interference (>20% of LLOQ) was observed in 6 different Sources of K_2EDTA human plasma
Inter-assay accuracy (bias)	2.5% (LLOQ)<br>-1.3% to 0.2% (above LLOQ)
Inter-assay precision	7.3% (LLOQ)<br>2.7% to 3.5% (above LLOQ)
Intra-assay accuracy (bias)	-0.5% to 6.0% (LLOQ)<br>-4.0% to 1.8% (above LLOQ)
Intra-assay precision	4.6% to 10.7% (LLOQ)<br>1.1% to 4.4% (above LLOQ)
Matrix Effect	LQC: 2.3 (CV%)<br>HQC: 1.8 (CV%)
Hemolytic and lipemic evaluation	Were acceptable (% bias and % CV at each level were within ± 15.0%)
Dilution integrity	Concentration: 15000.0 pg/mL<br>Dilution: 2-fold<br>Accuracy -3.3%, Precision 2.0%<br><br>Concentration: 80000.0 ng/mL<br>Dilution: 5-fold<br>Accuracy -5.0%, Precision 1.3%
Recovery of analyte	82.2%
Recovery of IS	58.9%
Refrigerator storage stability	Confirmed up to 5 days 21 hours
Freeze thaw stability	Confirmed up to four cycles at -20°C and -70°C
Bench top stability	Stable up to 16 hours at room temp
Injector Carryover	No significant (>20% of LLOQ) carryover was observed
Whole Blood	No instability observed in ice-water bath or at room temperature for up to 1.5 hour in polyethylene terephthalate containers.
Long term Stability in Plasma	Confirmed up to 64 days in polypropylene tubes at<br>-20°C and -70°C nominal
Abbreviations – HPLC: High performance liquid chromatography; LLOQ: Lower limit of quantification, IS: Internal standard; LQC: Low quality control; HQC: High quality control; CV: Coefficient of variance	

© 2024 Strauss DG et al. *JAMA Network Open.*

eMethods 2. Modeling

The Clinical Study Protocol and Statistical Analysis Plan (available in Supplement 1) specified the following Objective and Secondary Endpoint for this clinical trial and indicated that full details of the analysis would be described in the Model Analysis Plan (available in Supplement 1):

- Objective #2: To use the pharmacokinetic data from each of the 3 naloxone dosing schedules/doses to predict the time to reverse opioid-induced respiratory depression following different overdose scenarios based on pharmacokinetic/pharmacodynamic (PK/PD) models
- Secondary Endpoint #3: Predicted time to rescue a patient from simulated opioid-induced respiratory depression from fentanyl and carfentanil following medium and high overdose scenarios

The following summarizes key details of the model and its development and validation.[1]

Model

The model contains multiple mechanistic sub-models:

1. Pharmacokinetic models describing the plasma concentration of different opioids and the opioid antagonist, naloxone, for different doses, formulations, and administration schedules.
2. A receptor binding model describing kinetic binding of opioids and opioid antagonists (naloxone) to the opioid μ-receptor.
3. A physiological model describing gas (oxygen and carbon dioxide) storage and exchange, and blood flow control:
   a) Gas (oxygen and carbon dioxide) storage and exchange incorporates three tissue compartments: the lungs, brain tissue and other body tissues combined together.
   b) Blood flow control incorporates local mechanisms that regulate blood flow to organs with higher metabolic requirements, as well as systemic mechanisms to trigger cardiovascular collapse and subsequently cardiac arrest due to severe, prolonged hypoxia.
   c) Ventilatory control incorporates the action of the central and peripheral chemoreceptor, and the effect of gases on respiratory neuron activity.
4. A pharmacodynamic model describing the relationship between the fraction of opioid agonist-bound μ-receptor (due to the competitive binding between opioid agonists and antagonists) and human ventilatory response.

Pharmacokinetic model

For the fentanyl PK model, a published PK model for fentanyl[2] was used. Because there was no reliable human pharmacokinetic data available for carfentanil, the fentanyl PK model was used as a template, and limited human carfentanil plasma data[3] was used to adjust the carfentanil PK model.

For the intranasal naloxone PK model, the PK model clinical data from this study were used to develop the PK model, to take into consideration how intranasal pharmacokinetics may be impacted with repeated dosing to the same nostril, including at different timings after the first dose.

For the intravenous naloxone PK model, a previously published model was used.[4]

© 2024 Strauss DG et al. *JAMA Network Open.*

Table – Summary of Clinical PK Data Used to Develop the Model

<table>
<tr><th></th><th>Model simulation in comparison to clinical data</th></tr>
<tr><td>Fentanyl IV</td><td>Previously published model based on 22 adults with escalating doses of IV fentanyl.[2]</td></tr>
<tr><td>Naloxone IN</td><td>Model reproduced the time courses of plasma naloxone concentration in the 3 treatment arms in the present study and prior data from the sponsor development program.[5]

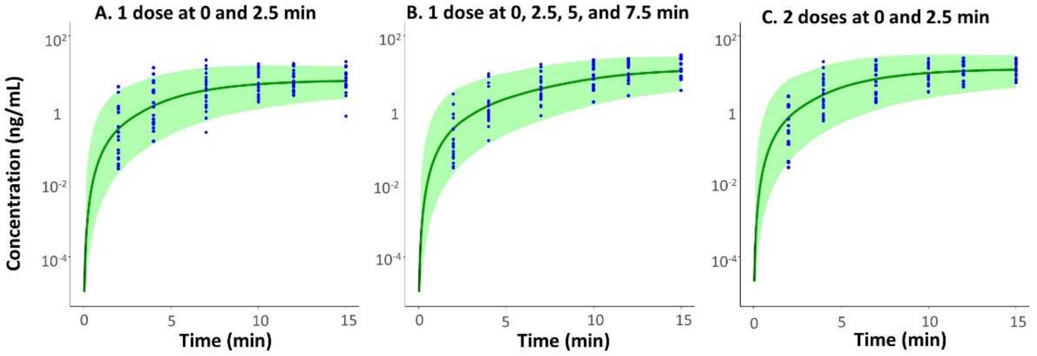

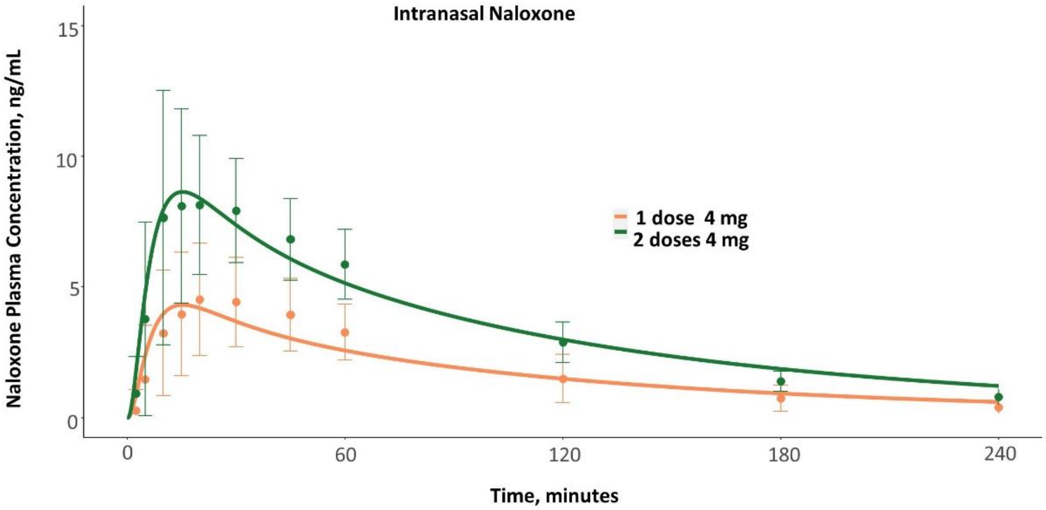
</td></tr>
<tr><td>Naloxone IV</td><td>Previously published model[4] based on 8 subjects receiving a three-stage stepwise high-dose IV naloxone infusion. Model independently reproduced 2 mg naloxone IV bolus injection data.[6]

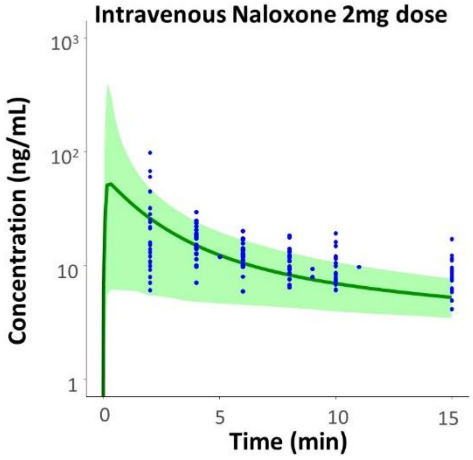
</td></tr>
</table>

© 2024 Strauss DG et al. *JAMA Network Open.*

Receptor binding model

For the receptor binding model, in vitro association, and dissociation experiments between ligands (i.e., fentanyl, carfentanil, and naloxone) and human opioid μ-receptor were used to estimate drug-specific binding parameters K_{on}, K_{off}, and n (Hill coefficient). The estimated binding parameters and equations were validated using dose-dependent dislocation data of individual opioids from the μ-receptor when combined with naloxone.

Table – Summary of in vitro receptor binding data used to develop the model

**Experiment**	**Model simulation in comparison to experiment**
Kinetics of association of opioids or naloxone with opioid receptors	Model reproduced time-dependent increase of the fraction of opioid receptors bound by opioids or naloxone (Mann et al. Figure S1).[1]
Kinetics of dissociation of opioids or naloxone from opioid receptors	Model reproduced time-dependent decrease of the fraction of opioid receptors bound by opioids (Mann et al. Figure S2).[1]
Displacement of opioids from opioid receptors by naloxone	Model reproduced the decrease of the fraction of opioid receptors bound by opioid agonists with increasing concentration of naloxone (Mann et al. Figure 5).[1]

Physiologic model

The physiological model (combining oxygen and carbon dioxide storage and exchange, ventilatory control, and blood flow control) was implemented based on the work of Magosso, Ursino and colleagues[7-9] with some modifications. Their model has been shown to reproduce a wide range of clinical data covering different combinations of carbon dioxide and oxygen changes and their impact on human ventilation with and without opioid administration.

Table – Summary of Physiologic Data used to Develop the Model

**Physiologic Response**	**Comparison of Model to Studies in Literature**
Ventilation Response to Isooxic Hypercapnia	Model reproduced the minute ventilation change in response to a hypercapnic stimulus (end-tidal partial pressure of carbon dioxide elevated to ~48 mm Hg) for constant hyperoxia, normoxia, and hypoxia (end-tidal partial pressure of oxygen fixed at 200, 100, and 53 mm Hg), respectively.[7]
Ventilation Under Hyperoxia and Normocapnia	Model reproduced the phenomenon of maintained minute ventilation under hyperoxia with normocapnia (e.g., end-tidal partial pressure of oxygen fixed at 200 mm Hg while end-tidal partial pressure of carbon dioxide at 40 mm Hg) (Mann et al. Figure S4).[1]
Ventilation Response to Isocapnic Hypoxia	Model reproduced the minute ventilation change in response to a hypoxic stimulus (end-tidal partial pressure of oxygen decreased to ~50 mm Hg) in the background of constant normocapnia and hypercapnia (end-tidal partial pressure of carbon dioxide fixed at 39 and 46 mm Hg), respectively (Mann et al. Figure S5).[1]
Relationship between arterial oxygen partial pressure and minute ventilation volume	Model reproduced the decrease of arterial oxygen partial pressure and increase of minute ventilation when end-tidal partial pressure of oxygen in the inspired air was gradually decreased (from 149 to 40 mm Hg) while end-tidal partial pressure of carbon dioxide was fixed at 40, 45, and 56 mm Hg, respectively (Mann et al. Figure S6).
Ventilation Response to	Model reproduced the changes in minute ventilation, end-tidal partial pressure of carbon dioxide, and arterial oxygen saturation in response to a hypoxic stimulus (end-

© 2024 Strauss DG et al. *JAMA Network Open.*

Poikilocapnic Hypoxia	tidal partial pressure of oxygen decreased to ~85 mm Hg) when the alveolar carbon dioxide partial pressure was allowed to vary naturally (Mann et al. Figure S7).[1]
Cerebral Blood Flow Changes	Model reproduced cerebral brain flow changes in response to carbon dioxide changes and simultaneous oxygen and carbon dioxide changes (Mann et al. Figure 2).[1]
Changes in Arterial Oxygen and Carbon Dioxide Partial Pressures During Sleep and Anesthesia	Model reproduced the decreased minute ventilation volume, arterial oxygen partial pressure, and increased arterial carbon dioxide partial pressure during sleep and the rise of arterial carbon dioxide and changes of arterial oxygen for preoxygenated and anesthetized patients when the ventilator was briefly disconnected and airway briefly blocked (Mann et al. Figure S8).[1]
Cardiovascular Compensation, Collapse, and Cardiac Arrest Due to Prolonged Hypoxia	Model reproduced the time course of cardiovascular compensation (increase in cardiac output), collapse and cardiac arrest in dogs breathing low levels of inspired oxygen and in pigs under asphyxia, as well as plasma oxygen and carbon dioxide changes during asphyxia-induced cardiac arrest in a horse study (Mann et al. Figure 3).[1]
Change in Volume of Distribution	Model reproduced the up to 2-fold reduction in the volume of distribution of opioids during the compensation period.[1]

Pharmacodynamic model

A pharmacodynamic component was used to link the receptor binding model to the physiology model, allowing the prediction of human ventilation due to different levels of μ-receptor binding (by opioids). The pharmacodynamic component describes the relationship between receptor occupancy and human ventilation response, which was estimated based on fentanyl data,[2,9] and then validated by comparing model prediction to other clinical data involving different fentanyl derivatives.

Table – Summary of Pharmacodynamic Data Used to Develop the Model.

**Clinical assessment**	**Model simulation in comparison to clinical data**
Impact of fentanyl bolus injection on ventilation while breathing room air	Model reproduced the changes in minute ventilation, arterial oxygen and carbon dioxide levels as well as the reduction and recovery of the ventilatory response to hypercapnia in a study where healthy opioid naïve participants were given a bolus intravenous injection of fentanyl while breathing room air (Mann et al. Figure 3).[1]
Impact of fentanyl on ventilation in healthy participants and chronic opioid users under isohypercapnia conditions	Model reproduced the changes in minute ventilation in a study where healthy participants and chronic opioid user participants were given intravenous fentanyl over 90 seconds in escalating amounts while end-tidal partial pressure of carbon dioxide was fixed at ~50 mm Hg (Mann et al. Figure 4).[1]
Impact of fentanyl infusion on ventilation while breathing room air	Model reproduced the changes in minute ventilation, arterial oxygen and carbon dioxide levels in a study with computer-driven continuous infusion of fentanyl while subjects were breathing room air (Mann et al. Figure 6).[1]
Impact of alfentanil infusion on ventilation while breathing room air	Model reproduced the changes in minute ventilation, arterial oxygen and carbon dioxide levels in a study with computer-driven continuous infusion of alfentanil while subjects were breathing room air (Mann et al. Figure 6).[1]
Impact of remifentanil infusion on ventilation under hyperoxia	Model reproduced the changes in minute ventilation in a study with remifentanil infusion while subjects were breathing 100% oxygen (Mann et al. Figure 6).[1]

© 2024 Strauss DG et al. *JAMA Network Open.*

eFigure 1. Model-predicted Outcomes of Fentanyl and Carfentanil Overdoses with and without Naloxone.

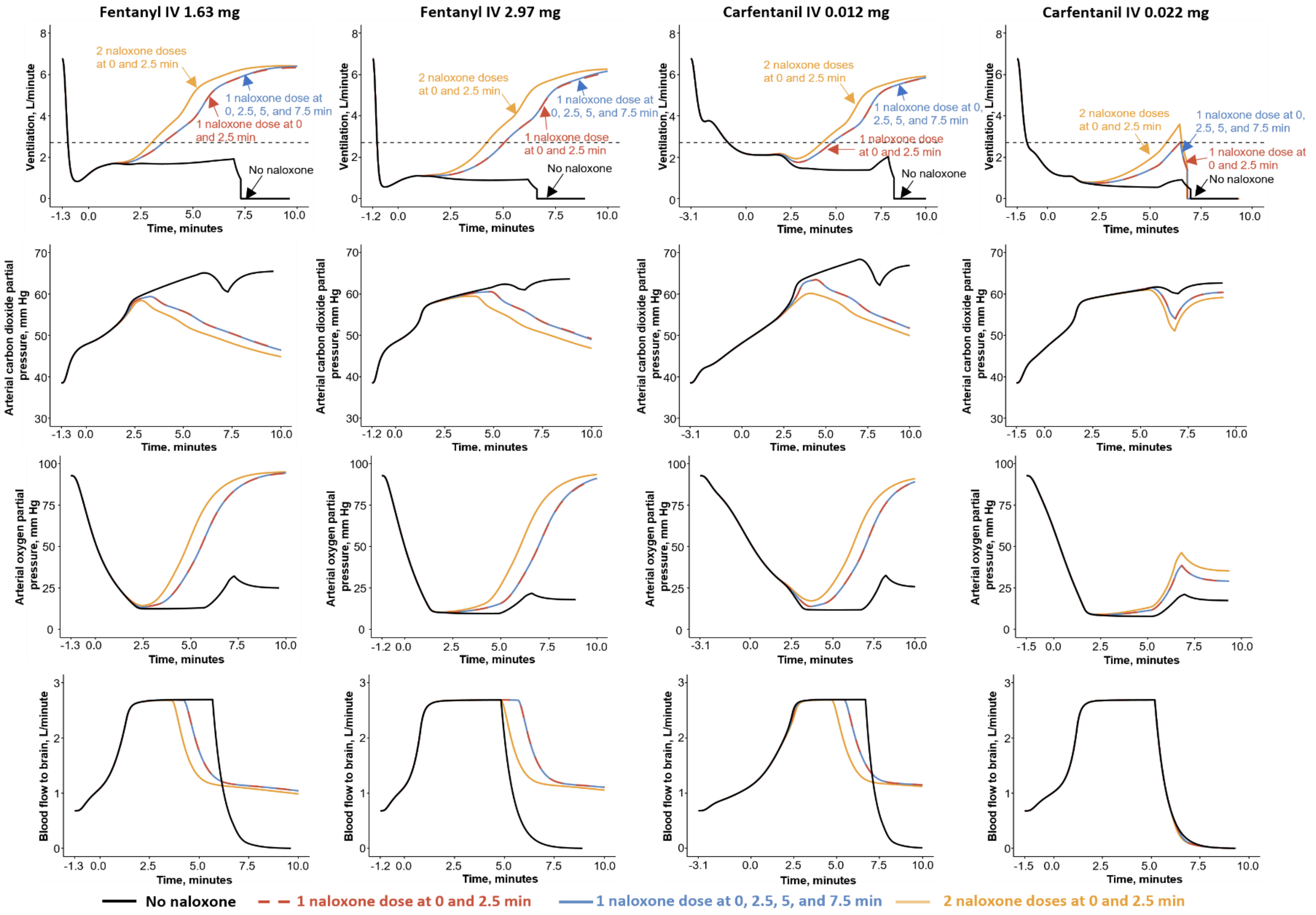

Each graph begins with the time of fentanyl or carfentanil administration. The first dose of intranasal naloxone 4 mg was administered 1 minute after ventilation decreased below 40% of baseline (i.e., first naloxone dose at 0.0 minutes in each graph). The dotted black line is 40% of baseline ventilation. See Figure 3 for other outcomes.

© 2024 Strauss DG et al. *JAMA Network Open.*

eFigure 2. Model-predicted Cardiac Arrest Outcomes with Carfentanil Overdoses and Intranasal or Intravenous Naloxone.

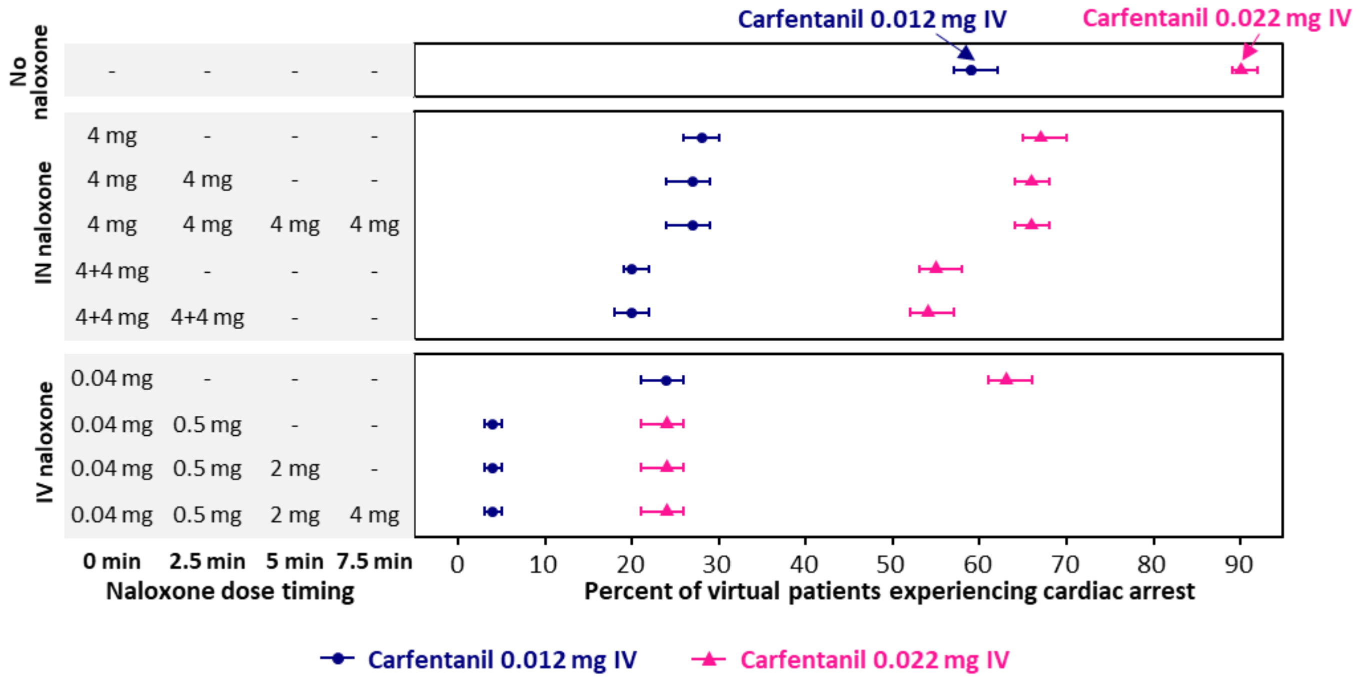

Model-predicted percentage of simulated patients experiencing cardiac arrest following carfentanil overdoses with different intranasal (IN) and intravenous (IV) naloxone dosing. The first naloxone dose was administered 1 minute after ventilation decreased below 40% of baseline. The intravenous naloxone model is from Papathanasiou et al.[4] The intravenous naloxone escalating dosing protocol is as described by Boyer[10] and is provided for comparative purposes. The points and error bars represent the median and interquartile range of cardiac arrest percentage.

© 2024 Strauss DG et al. *JAMA Network Open.*

eFigure 3. Effect of Changing the Delay between Ventilatory Depression and the First Naloxone Dose.

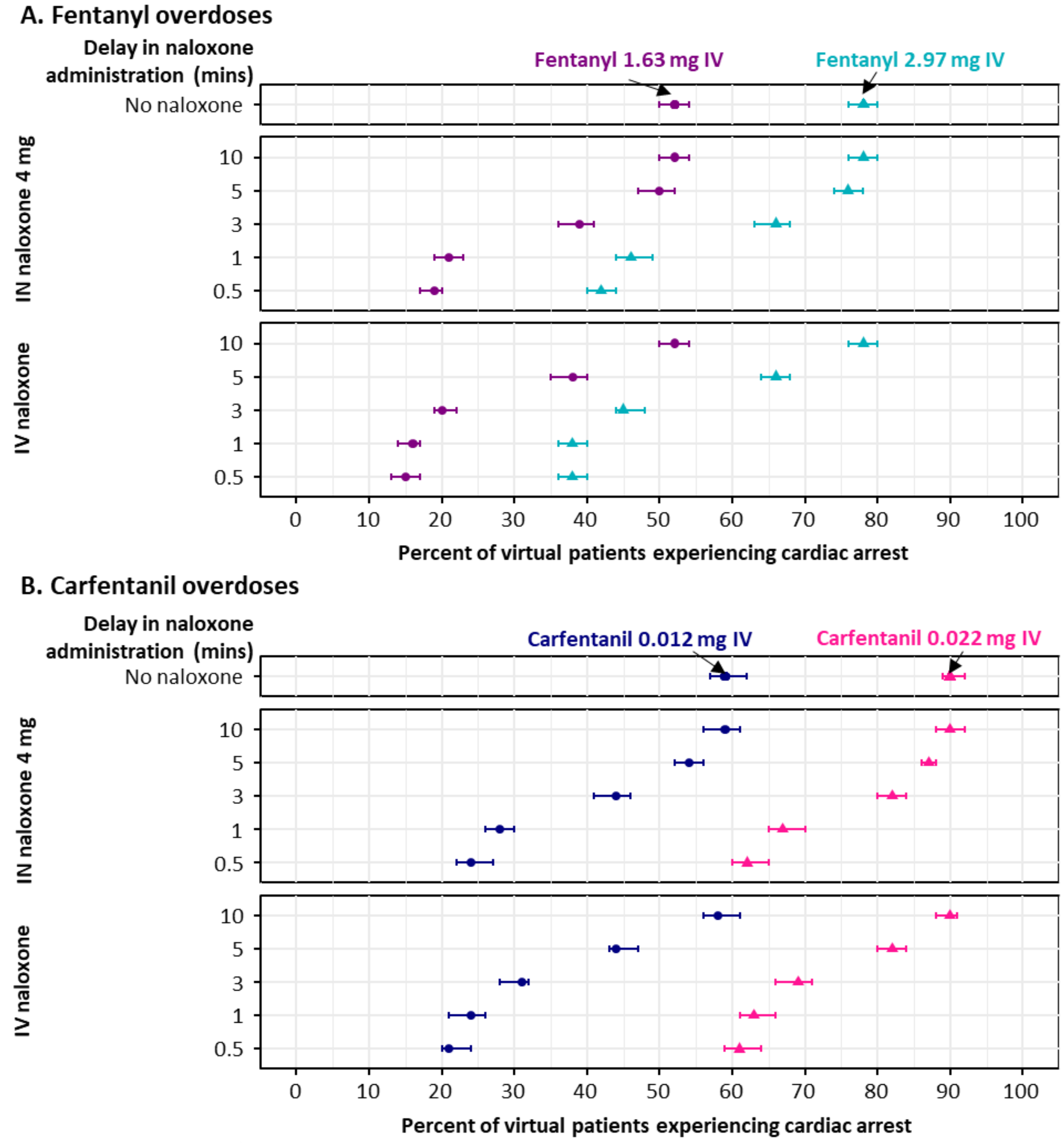

Model-predicted percentage of simulated patients experiencing cardiac arrest following fentanyl (Panel A) and carfentanil (Panel B) overdoses with different delays from the time ventilation decreased below 40% of baseline to administration of a single intranasal 4 mg/0.1 ml naloxone dose or intravenous 0.04 mg naloxone dose. The points and error bars represent the median and interquartile range of cardiac arrest percentage.

© 2024 Strauss DG et al. *JAMA Network Open.*

eTable 1. Exploratory Outcomes

1) Pharmacokinetic Outcomes	
**Outcomes**<br><br>• Maximum concentration (C_{max})<br><br>• Area under the curve from time 0 extrapolated to infinity (AUC_{0-inf})<br><br>• Area under the curve from time 0 to the sampling time corresponding to the last quantifiable concentration (C_{last}) (AUC_{0-t})<br><br>• Time at which C_{max} occurs (T_{max})<br><br>• Partial area under the curve (pAUC)	**Comments**<br><br>• AUC_{0-inf} was extrapolated to infinity from the AUC from time 0 to the sampling time corresponding to the last quantifiable concentration plus the last quantifiable concentration divided by the terminal elimination rate constant<br><br>• AUC_{0-t} was calculated according to the linear trapezoidal rule from time 0 minutes to the last quantifiable concentration.<br><br>• pAUC was calculated according to the linear trapezoidal rule from time 0 minutes to 7, 10, 12.5, 15, 20, and 30 minutes after time from first dose
**2) Physiologic Pharmacokinetic-Pharmacodynamic Model Outcomes**	
**Outcomes**<br><br>• Percentage of simulated patients predicted to experience cardiac arrest<br><br>• Model-predicted rescue times for different physiological thresholds<br>○ Arterial partial pressure of oxygen > 30 mm Hg<br>○ Arterial oxygen saturation ≥ 90%<br>○ Arterial partial pressure of carbon dioxide ≤ 45 mm Hg	**Comments**<br><br>Rationale for different physiological thresholds is as follows:<br>• Arterial partial pressure of oxygen > 30 mm Hg<br>○ Above the approximate upper range of arterial oxygen partial pressure at the time of cardiac arrest in animal models of asphyxia<br>• Arterial oxygen saturation ≥ 90%<br>○ Above this the hemoglobin saturation curve flattens such that further increases in oxygen partial pressure have less effect on oxygen saturation<br>• Arterial partial pressure of carbon dioxide ≤ 45 mm Hg<br>○ Upper range of normal for arterial partial pressure of carbon dioxide

© 2024 Strauss DG et al. *JAMA Network Open.*

eTable 2. Study Participant Demographics

Demographic	Population Total (N=21)
Age, median (IQR), y	34 (27 to 50)
Sex	
Male, No. (%)	11 (52)
Female, No. (%)	10 (48)
Race[a]	
Black or African American, No. (%)	9 (43)
White, No. (%)	11 (52)
No Race Reported, No. (%)	1 (5)
Hispanic or Latino Ethnicity[a], No. (%)	4 (19)
Body weight, median (IQR), kg	79.6 (67.6 to 85.1)
Body mass index, median (IQR), kg/m^2	28.4 (24.8 to 30.6)

Abbreviations: IQR, Interquartile range; No., number.
[a]Self-identified race and ethnicity were collected in an open-ended format by clinical staff.

© 2024 Strauss DG et al. *JAMA Network Open.*

eTable 3. Number of participant samples included in analyses

Time, min	Number of Participant Samples		
	1 dose at 0 and 2.5 min	1 dose at 0, 2.5, 5, and 7.5 min	2 doses at 0 and 2.5 min
0 (predose)	18	18	18
2	16	18	17
4.5	16	18	18
7	16	17	18
10	16	18	18
12.5	17	18	17
15	17	17	18
20	17	17	17
30	18	17	17
45	18	18	17
60	18	18	17
120	18	18	18
180	18	18	17
240	18	18	17
360	18	17	18
720	15	15	15

Not including the predose time, 3 plasma samples were below the lower limit of quantification (all at 2 minutes). Thirty plasma samples were outside of the protocol-specified collection time (other time points with n<18).

© 2024 Strauss DG et al. *JAMA Network Open.*

eTable 4. Primary Pharmacokinetic Outcomes

Time, min	Geometric Mean Naloxone Concentration, ng/mL (CV%)[a]			Geometric Mean Ratio (1-sided 97.8% CI) at First Time with Higher Concentration	
	1 dose at 0 and 2.5 min (N=18)	1 dose at 0, 2.5, 5, and 7.5 min (N=18)	2 doses at 0 and 2.5 min (N=18)	1 dose at 0, 2.5, 5, and 7.5 min vs. 1 dose at 0 and 2.5 min	2 doses at 0 and 2.5 min vs. 1 dose at 0 and 2.5 min
2	0.17 (375%)	0.15 (224%)	0.20 (246%)	-	-
4.5	1.23 (250%)	1.33 (124%)	2.24 (134%)	-	1.98 (1.03 to ∞)
7	2.95 (142%)	4.40 (99%)	5.29 (111%)	-	-
10	4.42 (159%)	7.95 (72%)	8.49 (70%)	1.95 (1.28 to ∞)	-
12.5	6.31 (82%)	12.01 (64%)	12.27 (60%)	-	-
15	6.56 (98%)	14.73 (58%)	13.70 (54%)	-	-
20	8.57 (52%)	14.20 (42%)	12.17 (44%)	-	-
30	7.47 (36%)	11.84 (27%)	10.13 (33%)	-	-
45	6.54 (27%)	9.76 (25%)	9.28 (30%)	-	-
60	5.23 (24%)	8.33 (28%)	7.22 (27%)	-	-
120	2.68 (28%)	4.48 (38%)	3.95 (30%)	-	-
180	1.53 (30%)	2.57 (41%)	2.19 (30%)	-	-
240	0.88 (31%)	1.52 (43%)	1.40 (44%)	-	-
360	0.31 (28%)	0.51 (50%)	0.49 (51%)	-	-
720	0.05 (27%)	0.10 (44%)	0.10 (60%)	-	-

[a]The pre-specified times for comparison of 1 dose at 0, 2.5, 5, and 7.5 minutes vs. 1 dose at 0 and 2.5 minutes were 10, 12.5 and 15 minutes; the pre-specified times for comparison of 2 doses at 0 and 2.5 minutes vs. 1 dose at 0 and 2.5 minutes were 4.5, 7, and 10 minutes. eTable 3 contains the number of participant samples included at each time for each dosing group. Abbreviations: CI, confidence interval; CV, coefficient of variation; min, minute

© 2024 Strauss DG et al. *JAMA Network Open.*

eTable 5. Secondary Pharmacokinetic Outcomes

Outcome	N	Geometric Mean Plasma Concentration, ng/mL (CV%)		Geometric Mean Ratio (1-sided 97.8% CI)
**Secondary outcome**				
First Time with Higher Plasma Concentration		2 doses at 0 and 2.5 minutes	1 dose at 0, 2.5, 5, and 7.5 minutes	
4.5 min	18	2.24 (134%)	1.33 (124%)	1.69 (1.06 to ∞)
**Secondary outcomes - dose-normalized plasma concentration comparisons**				
**Plasma Concentration Parameter**		**AUC, ng/mL * min (CV%) or C_{max}, ng/mL (CV%)**		**Dose-normalized Geometric Mean Ratio (2-sided 90% CI)**
		1 dose at 0, 2.5, 5, and 7.5 minutes	1 dose at 0 and 2.5 minutes	
AUC	18	1606 (25%)	978 (20%)	0.82 (0.75 to 0.89)
C_{max}	18	17.6 (36%)	10.0 (48%)	0.89 (0.78 to 1.00)
		2 doses at 0 and 2.5 minutes	1 dose at 0 and 2.5 minutes	
AUC	18	1455 (26%)	978 (20%)	0.74 (0.69 to 0.80)
C_{max}	18	15.4 (44%)	10.0 (48%)	0.77 (0.65 to 0.92)

Abbreviations: AUC, area under the plasma concentration-time curve; CI, confidence interval; C_{max}, maximum plasma concentration; CV, coefficient of variation

© 2024 Strauss DG et al. *JAMA Network Open.*

eTable 6. Exploratory Pharmacokinetic Measures by Naloxone Treatment Group

	Naloxone Treatment Group		
	**1 dose at 0 and 2.5 minutes (n=18)**	**1 dose at 0, 2.5, 5, and 7.5 minutes (n=18)**	**2 doses at 0 and 2.5 minutes (n=18)**
Maximum concentration, ng/mL (CV%)	10.0 (48%)	17.6 (36%)	15.4 (44%)
Median time to maximum concentration, min (IQR)	20 (16.3 to 30)	15 (15 to 20)	15 (15 to 20)
AUC_{inf}, ng/mL * min (CV%)	978 (20%)	1606 (25%)	1455 (26%)
AUC_{0-t}, ng/mL * min (CV%)	970 (20%)	1587 (25%)	1437 (26%)
AUC_{0-7}, ng/mL * min (CV%)	6.0 (275%)	9.2 (108%)	12.9 (121%)
AUC_{0-10}, ng/mL * min (CV%)	17.2 (176%)	28.0 (87%)	34.6 (93%)
$AUC_{0-12.5}$, ng/mL * min (CV%)	28.8 (176%)	54.1 (74%)	58.6 (87%)
AUC_{0-15}, ng/mL * min (CV%)	44.9 (137%)	86.3 (68%)	93.9 (70%)
AUC_{0-20}, ng/mL * min (CV%)	84.0 (97%)	162.7 (54%)	161.4 (57%)
AUC_{0-30}, ng/mL * min (CV%)	167.7 (37%)	298.7 (40%)	279.5 (46%)

Abbreviations: CV, coefficient of variation; IQR, interquartile range, AUC_{inf}, area under the curve from 0 minutes to infinity, AUC_{0-t}, area under the curve from 0 minutes to the last sample (typically 720 minutes); AUC_{0-7}, area under the curve from 0 to 7 minutes; AUC_{0-10}, area under the curve from 0 to 10 minutes; $AUC_{0-12.5}$, area under the curve from 0 to 12.5 minutes; AUC_{0-15}, area under the curve from 0 to 15 minutes; AUC_{0-20}, area under the curve from 0 to 20 minutes; AUC_{0-30}, area under the curve from 0 to 30 minutes

© 2024 Strauss DG et al. *JAMA Network Open.*

eTable 7. Incidence and Number of Adverse Events by Treatment Group

Adverse Event[a]	Incidence (number of events)		
	**1 dose at 0 and 2.5 minutes (n=21)**	**1 dose at 0, 2.5, 5, and 7.5 minutes (n=21)**	**2 doses at 0 and 2.5 minutes (n=21)**
Abdominal Pain	1 (1)	0 (0)	0 (0)
Asthenia	1 (1)	0 (0)	0 (0)
Dizziness	1 (1)	0 (0)	0 (0)
Headache	1 (1)	1 (1)	0 (0)
Nasal Discomfort	5 (8)	8 (9)	5 (6)
Nausea	0 (0)	1 (1)	0 (0)
Oropharyngeal Pain	0 (0)	1 (1)	0 (0)
Pollakiuria	0 (0)	1 (1)	0 (0)
Restless Leg Syndrome	1 (1)	0 (0)	0 (0)
Retching	0 (0)	1 (1)	0 (0)
Somnolence	1 (1)	0 (0)	0 (0)
Syncope	1 (1)	0 (0)	0 (0)
Vessel Puncture Site Pain	2 (2)	0 (0)	0 (0)
Vomiting	1 (1)	1 (1)	0 (0)

[a]This table reports all Medical Dictionary for Regulatory Activities (MedDRA v.23.0)-defined adverse events during the study.

© 2024 Strauss DG et al. *JAMA Network Open.*

eTable 8. Model-Predicted Rescue Times Based on Different Dosing Scenarios and Respiratory Measures

Opioid and Intranasal Naloxone (4 mg/0.1 mL) Dosing	Time (minutes)[a]		
	Arterial Oxygen Partial Pressure ≤30 mm Hg, Median (IQR)	Arterial Oxygen Saturation ≤90%, Median (IQR)	Arterial Carbon Dioxide Partial Pressure ≥45 mm Hg, Median (IQR)
Fentanyl, 1.63			
No naloxone	∞ (0 to ∞)	∞ (8.9 to ∞)	∞ (58.3 to ∞)
1 dose at 0 minutes	3.3 (0 to 5.5)	6.4 (4.6 to 8.8)	13.9 (10.8 to 20.4)
1 dose at 0 and 2.5 minutes	3.2 (0 to 5.3)	6.2 (4.5 to 8.3)	12.5 (9.9 to 17.3)
1 dose at 0, 2.5, 5, and 7.5 minutes	3.2 (0 to 5.3)	6.2 (4.5 to 8.3)	12.3 (9.8 to 16.5)
2 doses at 0 minutes	2.6 (0 to 4.5)	5.5 (4.1 to 7.3)	11.5 (8.8 to 15)
2 doses at 0 and 2.5 minutes	2.6 (0 to 4.5)	5.4 (4.1 to 7.1)	10.9 (8.5 to 13.7)
Fentanyl, 2.97			
No naloxone	∞ (∞ to ∞)	∞ (∞ to ∞)	∞ (∞ to ∞)
1 dose at 0 minutes	5.4 (3.1 to ∞)	8.5 (6.1 to ∞)	19.4 (13.2 to ∞)
1 dose at 0 and 2.5 minutes	5.2 (3.1 to ∞)	8.1 (6 to ∞)	16.4 (11.8 to ∞)
1 dose at 0, 2.5, 5, and 7.5 minutes	5.2 (3.1 to ∞)	8.1 (6 to ∞)	15.6 (11.6 to ∞)
2 doses at 0 minutes	4.4 (2.4 to ∞)	7 (5.2 to ∞)	13.9 (10.7 to ∞)
2 doses at 0 and 2.5 minutes	4.3 (2.4 to ∞)	6.9 (5.2 to ∞)	12.9 (10.1 to ∞)
Carfentanil, 0.012			
No naloxone	∞ (0 to ∞)	∞ (9.7 to ∞)	∞ (>60 to ∞)
1 dose at 0 minutes	3.6 (0 to ∞)	8.7 (6.5 to ∞)	>60 (21 to ∞)
1 dose at 0 and 2.5 minutes	3.5 (0 to ∞)	8.5 (6.5 to ∞)	>60 (18.6 to ∞)
1 dose at 0, 2.5, 5, and 7.5 minutes	3.5 (0 to ∞)	8.5 (6.5 to ∞)	>60 (18.1 to ∞)
2 doses at 0 minutes	2.7 (0 to 5.4)	7.7 (5.9 to 11)	>60 (17.4 to >60)
2 doses at 0 and 2.5 minutes	2.7 (0 to 5.3)	7.6 (5.9 to 10.7)	>60 (16.6 to >60)
Carfentanil, 0.022			
No naloxone	∞ (∞ to ∞)	∞ (∞ to ∞)	∞ (∞ to ∞)
1 dose at 0 minutes	∞ (5.2 to ∞)	∞ (9.4 to ∞)	∞ (>60 to ∞)
1 dose at 0 and 2.5 minutes	∞ (5.1 to ∞)	∞ (8.9 to ∞)	∞ (22.3 to ∞)
1 dose at 0, 2.5, 5, and 7.5 minutes	∞ (5.1 to ∞)	∞ (8.9 to ∞)	∞ (20.9 to ∞)
2 doses at 0 minutes	∞ (4.3 to ∞)	∞ (7.8 to ∞)	∞ (18.6 to ∞)
2 doses at 0 and 2.5 minutes	∞ (4.2 to ∞)	∞ (7.6 to ∞)	∞ (17.3 to ∞)

Abbreviations: IQR, interquartile range

[a]For cases where the recovery of a specific simulated physiological measurement above prespecified levels as outlined in the table was not observed within the simulated time, the rescue time is reported as >60 minutes. The rationale for the arterial oxygen and carbon dioxide thresholds is outlined in the Model Analysis Plan (available in Supplementary 1).

eAppendix. Data Dictionary for Naloxone Pharmacokinetic Data Set

A full listing of participant data, including demographics and concentrations for each treatment group, day, and time point are included with the Supplementary Material. Column names and definitions are provided below:

Pharmacokinetic data set

Variable	Definition
Participant	Participant number
Period	One of 1, 2, or 3, corresponding to study period when data was collected
Time	Nominal sampling time (unit = hour)
Concentration_ngmL	Reported concentration with values below lower limit of quantification set to zero. (units = ng/mL)
BLQ	Concentration values below the limit of quantification (0.020 ng/mL) (1 for below the limit of quantification and 0 for above the limit of quantification)
Age_Decade	Decade age range of the participant (unit = ’21-30’, ’31-40’, etc.)
Sex	Sex of the participant (one of 'Female' or 'Male')
Weight	Weight of the participant (unit = kg)
BMI	Body mass index (unit = kg/m^2)
Treatment_Group	Treatment group (one of ‘1 dose at 0 and 2.5 min’, ‘1 dose at 0, 2.5, 5, and 7.5 min’, or ‘2 doses at 0 and 2.5 min’)

© 2024 Strauss DG et al. *JAMA Network Open.*

eReferences

1. Mann J, Samieegohar M, Chaturbedi A, et al. Development of a Translational Model to Assess the Impact of Opioid Overdose and Naloxone Dosing on Respiratory Depression and Cardiac Arrest. *Clin Pharmacol Ther*. Nov 2022;112(5):1020-1032. doi:10.1002/cpt.2696
2. Algera MH, Olofsen E, Moss L, et al. Tolerance to Opioid-Induced Respiratory Depression in Chronic High-Dose Opioid Users: A Model-Based Comparison With Opioid-Naive Individuals. *Clin Pharmacol Ther*. Mar 2021;109(3):637-645. doi:10.1002/cpt.2027
3. Minkowski CP, Epstein D, Frost JJ, Gorelick DA. Differential response to IV carfentanil in chronic cocaine users and healthy controls. *Addict Biol*. Jan 2012;17(1):149-55. doi:10.1111/j.1369-1600.2010.00256.x
4. Papathanasiou T, Springborg AD, Kongstad KT, et al. High-dose naloxone, an experimental tool uncovering latent sensitisation: pharmacokinetics in humans. *Br J Anaesth*. Aug 2019;123(2):e204-e214. doi:10.1016/j.bja.2018.12.007
5. Clinical Pharmacology and Biopharmaceutics Review for NDA 208411 (Narcan). US Food and Drug Administration. Accessed July 12, 2023. https://www.accessdata.fda.gov/drugsatfda_docs/nda/2015/208411Orig1s000ClinPharmR.pdf
6. Clinical Pharmacology and Biopharmaceutics Review for NDA 212045 (Kloxxado). US Food and Drug Administration. Accessed July 12, 2023. https://www.accessdata.fda.gov/drugsatfda_docs/nda/2021/212045Orig1s000ClinPharmR.pdf
7. Ursino M, Magosso E, Avanzolini G. An integrated model of the human ventilatory control system: the response to hypercapnia. *Clin Physiol*. Jul 2001;21(4):447-64. doi:10.1046/j.1365-2281.2001.00349.x
8. Ursino M, Magosso E, Avanzolini G. An integrated model of the human ventilatory control system: the response to hypoxia. *Clin Physiol*. Jul 2001;21(4):465-77. doi:10.1046/j.1365-2281.2001.00350.x
9. Magosso E, Ursino M, van Oostrom JH. Opioid-induced respiratory depression: a mathematical model for fentanyl. *IEEE Trans Biomed Eng*. Jul 2004;51(7):1115-28. doi:10.1109/TBME.2004.827344
10. Boyer EW. Management of opioid analgesic overdose. *N Engl J Med*. Jul 12 2012;367(2):146-55. doi:10.1056/NEJMra1202561

© 2024 Strauss DG et al. *JAMA Network Open.*
