# Supplementary material for: Intranasal Naloxone Repeat Dosing Strategies and Fentanyl Overdose: A Simulation-Based Randomized Clinical Trial
Source: JAMA Netw Open. 2024 Jan 23;7(1):e2351839. doi: 10.1001/jamanetworkopen.2023.51839 (PMC10807299; doi:10.1001/jamanetworkopen.2023.51839)
Supplement: Supplement 4. — Data Sharing Statement [file jamanetwopen-e2351839-s004.pdf]

Data Sharing Statement

Strauss. Intranasal Naloxone Repeat Dosing Strategies and Fentanyl Overdose. *JAMA Netw Open*. Published January 19, 2024. doi:10.1001/jamanetworkopen.2023.51839

Data
Data available: Yes
Data types: Deidentified participant data, Data dictionary
How to access data: Online supplement to the published article on the journal website
When available: With publication

Supporting Documents
Document types: Statistical/analytic code
How to access documents: https://github.com/FDA/Mechanistic-PK-PD-Model-to-Rescue-Opioid-Overdose
When available: With publication

Additional Information
Who can access the data: Anyone requesting the data
Types of analyses: For any purpose
Mechanisms of data availability: Downloadable without restriction
